# A formally exact theory to construct nonreactive forcefields using linear regression to optimize bonded parameters†

**DOI:** 10.1039/d4ra01861c

**Published:** 2024-10-22

**Authors:** Thomas A. Manz

**Affiliations:** a Chemical & Materials Engineering, New Mexico State University Las Cruces NM 88001 USA tmanz@nmsu.edu

## Abstract

This article derives theoretical foundations of force field functional theory (FFFT). FFFT studies topics related to the functional representation of nonreactive forcefields to achieve various desirable properties such as: (a) formal exactness of the forcefield's energy functional under certain conditions, (b) a formally exact ansatz separating the bonded potential energy from the nonbonded potential energy within a bonded cluster in a way that enables bonded parameters to be optimized using linear regression instead of requiring nonlinear regression, (c) the potential energy's continuous differentiability to various orders with respect to energetically accessible internal coordinate displacements within a subdomain defined by one electronic ground state, (d) forcefield design that guarantees the reference ground-state geometry is exactly reproduced as an equilibrium structure on the forcefield's potential energy landscape, (e) reasonably accurate and broadly applicable frugal model potentials, (f) computationally efficient embedded feature selection that identifies and removes unimportant forcefield terms, (g) well-designed methods to parameterize the forcefield from quantum-mechanically-computed and (optionally) experimental reference data, and (h) forcefields that approximately reproduce experimentally-measured properties. This article also introduces: (1) an angle-bending model potential that more accurately describes physical dynamics and is continuously differentiable to all orders with respect to internal coordinate displacements even when the bond angle is linear (*i.e.*, *θ* = π (180°)) and (2) a first-principles-derived stretch potential that accurately describes short-range Pauli repulsion and the long-range bond dissociation energy. This new angle-bending potential gave good agreement to CCSD quantum-chemistry calculations for CaH_2_, CO_2_, H_2_O, HNO, Li_2_O, NO_2_, NS_2_, SF_2_, SiH_2_, and SO_2_ molecules. This new bond-stretch potential reproduced the first 12+ and 30+ vibrational energy levels of H_2_ and O_2_ molecules, respectively, within a few percent of experimental values. Studying the C–F bond stretch in C_6_F_6_ as an example, the new ansatz (item (b) above) reduced sensitivity of the optimized force constant's value to choice of nonbonded interaction parameters by an order of magnitude compared to the old ansatz. Normal mode analysis of optimized flexibility models for CO_2_, H_2_O, HNO, and SO_2_ molecules yielded vibrational transition frequencies within a few percent of experimental values. These results demonstrate advantages of this new approach.

## Abstract 2

This article derives and tests: (1) a formally exact forcefield functional theory to construct non-reactive forcefields using linear regression for bonded parameters and (2) better bond-stretch and angle-bending model potentials.

## Graphical abstract

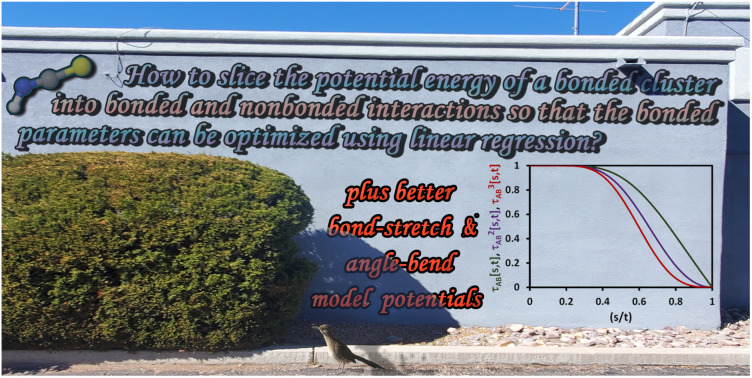

## 1. Introduction

In a nonreactive forcefield, the potential energy is often represented as the sum of bonded interactions and nonbonded interactions:^1–4^1
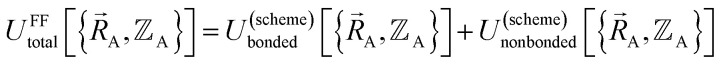


The independent variables, 
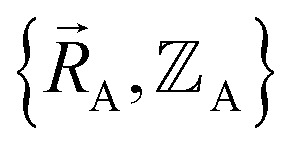
, define the material's chemical geometry. 
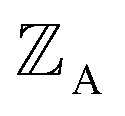
 is the element number (aka ‘atomic number’) of atom A. The position of atom A's nucleus is2
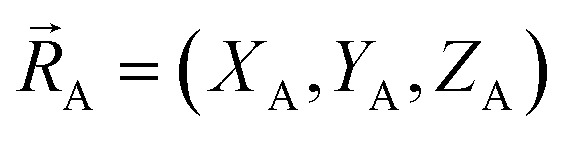


The bonded interactions include flexibility terms such as bond stretches, angle bends, dihedral torsions, Urey–Bradley terms, cross terms, out-of-plane distances, *etc.* between first, second, third, and/or more distant bonded neighbors.^1,5–7^ The nonbonded interactions account for interactions between atoms that are not directly bonded to each other. Nonbonded interactions include: (a) electrostatic interactions modeled by charges, dipoles and other multipoles, and/or polarizabilities, *etc.*, (b) short-range repulsion, (c) long-range dispersion interactions caused by fluctuating multipoles, *etc.*^8–14^ The superscript ‘(scheme)’ in eqn (1) reminds us that the partition of 
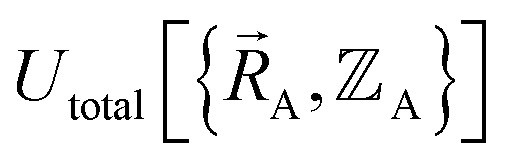
 into bonded and nonbonded interactions depends on the particular scheme chosen to define such a partition.

At the material's equilibrium (aka optimized) ground-state geometry, the net force acting on atom A3
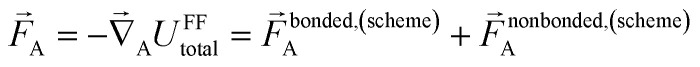
is zero4
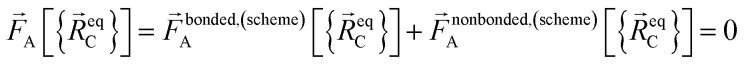
where5
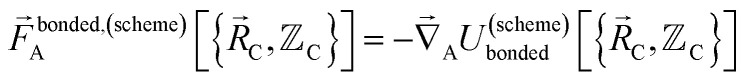
6
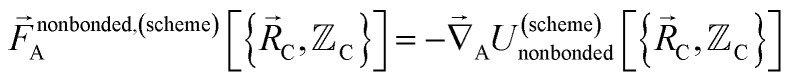
The distance (*d*_AB_) between atoms A and B is7
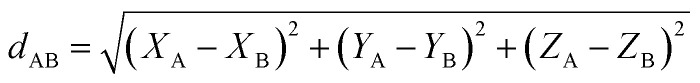
and has the equilibrium value *d*^eq^_AB_ in the material's optimized ground-state geometry.

A popular strategy (aka the ‘old’ scheme) is to define the nonbonded potential as a sum of pairwise nonbonded potentials plus optional multibody^15^ corrections:8
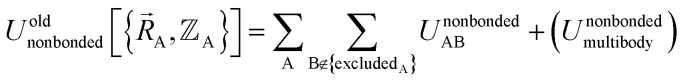
where {excluded_A_} is the set of atoms that are separated from atom A by 0 (*i.e.*, atom A itself), 1, 2, or (optionally) 3 bonds; that is, the set of atom A's zeroth (*i.e.*, atom A itself), first, second, and (optionally) third bonded neighbors.^16,17^ As an example, consider a nonbonded potential between two atoms A and B having a simple form involving atomic charges and Lennard-Jones^18^ parameters:9
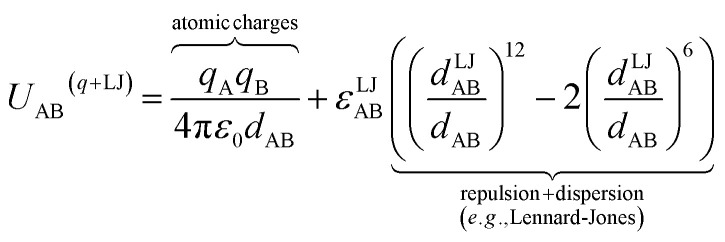
*ε*^LJ^_AB_ is the Lennard-Jones well-depth. *d*^LJ^_AB_ is the distance at which the well-depth is reached. More sophisticated examples of *U*^nonbonded^_AB_ may involve terms containing atomic dipoles or quadrupoles, atomic polarizabilities, and/or non-Lennard-Jones van der Waals parameters, *etc.*^14,19–21^

Nonbonded energy schemes defined by eqn (8) and (9) or similar equations encounter a major disadvantage when optimizing the forcefield's bonded parameter values. When using such a scheme, the nonbonded interactions between atoms may exhibit a nonzero force on atom A even in the material's optimized ground-state geometry. This is evident, because atom A is acted upon by electrostatic forces (*e.g.*, Coulomb interactions between atomic charges) and van der Waals (*e.g.*, Lennard-Jones) forces exerted by atoms outside {excluded_A_} such as atoms in the same bonded cluster that are separated from atom A by ≥4 bonds but still inside the nonbonded interaction cutoff distance, *d*^nonbonded^_cutoff_. By eqn (4), this means the bonded interactions must exhibit a net force of equal magnitude in the opposing direction so as to make the total bonded plus nonbonded force acting on each atom zero in the optimized ground-state geometry. Unfortunately, this means the forcefield's flexibility terms such as the harmonic bond stretching potential

10 *U*^harmonic_stretch^_AB_ = ½*k*_AB_(*d*_AB_ − *d*^ref^_AB_)^2^

have ‘resting’ values that are not necessarily equal to the equilibrium bond length

11 *d*^ref^_AB_ = *d*^resting^_AB_ ≠ *d*^eq^_AB_

This has been pointed out in the prior literature by several authors who devised schemes to approximately estimate these resting values.^22–27^ Because the resting values of bond lengths, angles, dihedrals, *etc.* enter the forcefield in a nonlinear fashion, this gives rise to a nonlinear optimization problem that may have several local minima.^26^

For the harmonic stretch, it is possible to rewrite eqn (10) as the linear model

12 *U*^harmonic_stretch^_AB_ = *p*_1_(*d*_AB_)^2^ + *p*_2_*d*_AB_ + *p*_3_

where the parameters *p*_1_ = ½*k*_AB_, *p*_2_ = −*k*_AB_*d*^ref^_AB_, and *p*_3_ = ½*k*_AB_(*d*^ref^_AB_)^2^. This allows the nonlinear optimization problem to be rewritten as a linear optimization problem. When using the old scheme, this kind of transformation from a nonlinear optimization problem into a linear optimization problem is not always feasible or practical, because it overly restricts the types of flexibility terms that could be included. For example, the old scheme could not be transformed into a linear optimization problem when the potential model includes the MM3 bond stretch^1,28^ term or when using *θ*^resting^_ABC_ ≠ *θ*^eq^_ABC_ as the reference value in some angle-bending potentials (*e.g.*, when using *θ*^resting^_ABC_ in place of *θ*^eq^_ABC_ in eqn (165)). Consequently, the old scheme is typically a nonlinear optimization problem and only reduces to a linear optimization problem for a restricted set of special cases.

The distinction between linear optimization problems (also called linear regression) and nonlinear optimization problems (also called nonlinear regression) is as follows. In linear regression, all adjustable parameter values to be optimized enter the model linearly as coefficients multiplied by functions of the independent variables.^29^ In nonreactive forcefields, the independent variables are the material's internal coordinates (*e.g.*, bond lengths, angles, dihedrals, *etc.*) that describe the material's geometry. These functions of the independent variables may contain fixed (*i.e.*, non-adjustable) parameters. For example, the equilibrium value of the bond angle between atoms A, B, and C (*θ*^eq^_ABC_) in the material's optimized ground-state geometry as determined by a quantum chemistry calculation may be treated as a fixed (*i.e.*, non-adjustable) parameter in the model forcefield, because the value of this parameter can be directly computed without requiring regression.

All linear optimization problems are convex. Technically, this means the Lagrangian (*i.e.*, the loss function including Lagrange multiplier terms to enforce constraints (such as bounds) on the optimized parameters) has only a single minimum value not that the optimized parameter values are unique, because some of the material's internal coordinates (and hence model forcefield's bonded terms) may be redundant (*i.e.*, multicollinear, not linearly independent). The resulting degeneracy of optimized parameter values can be suppressed *via* techniques that minimize a norm of the optimized parameters vector.^29–31^ Specifically, the least absolute shrinkage and selection operator (LASSO^32,33^) method minimizes the *L*_1_ norm (*i.e.*, the sum of absolute values) of the optimized parameters, while the Moore–Penrose pseudo-inverse^31,34,35^ and ridge regression^36^ methods minimize the squared *L*_2_ norm (*i.e.*, the sum of squares) of the optimized parameters.

In general, nonlinear optimization problems are more difficult to solve than linear optimization problems.^37,38^ Nonlinear optimization problems have more complicated landscapes that may in some cases be nonconvex with multiple local minima.^37,38^ As a consequence of this nonlinearity, it may be difficult to determine if the true global optimum of a model forcefield's bonded parameter values have been computed or if the optimizer only reached a local but not global optimum of the model forcefield's bonded parameter values.^38^ Some nonlinear optimization problems are provably convex with a single minimum. However, whether a particular nonlinear optimization problem is provably convex must be derived on a case-by-case basis, which requires detailed theoretical analysis.^39^ Sometimes it is not easily apparent whether a particular nonlinear optimization problem is convex or not. Furthermore, imposing bounds (or other constraints) on the regressed parameters (*e.g.*, constraining each bond stretch force constant to be non-negative) is generally more challenging for non-linear optimization problems than for linear optimization problems.^39,40^ Moreover, multicollinearity arising from internal coordinate redundancy is more difficult to treat during nonlinear regression compared to linear regression. Algorithms for solving nonlinear optimization problems include deterministic methods (*e.g.*, conjugate gradient and steepest descent) to find a local minimum, deterministic global optimizers to find a global minimum, and stochastic methods (*e.g.*, genetic and particle swarm) to find a global minimum.^38–41^

In this article, I introduce a new theoretical framework that transforms the task of optimizing values for bonded parameters (aka flexibility parameters) in nonreactive forcefields from a nonlinear optimization problem into a linear optimization problem. As described in Section 2 below, this is accomplished by introducing a new ansatz for separating the forcefield's potential energy into bonded and nonbonded potential energy terms. My scheme formally decouples the bonded interactions from the nonbonded interactions in a way that zero-, first-, and second-order derivatives of an isolated bonded cluster's potential energy function at its optimized ground-state geometry depend only on the bonded interactions with no dependence on nonbonded interactions. This allows the bonded interaction terms to use equilibrium values directly from the material's quantum-mechanically-computed optimized ground-state geometry instead of separate ‘resting values’ that would require nonlinear regression. Moreover, this reduces sensitivity of the optimized force constants values appearing in the bonded interaction terms to the particular choice of nonbonded interaction model. Fortunately, this is done in a formally exact way under certain conditions that does not restrict the forcefield from exactly reconstructing the material's true potential energy function.

In practice, a finite cutoff distance for the nonbonded interactions is sometimes used to enhance computational efficiency.^42,43^ When using a nonbonded interaction cutoff distance, my approach yields continuous zero-, first-, and second-order derivatives of the potential energy even at the cutoff distance. In contrast, most prior approaches yielded either discontinuous forces or discontinuous second-order derivatives at the cutoff distance.^42–44^

In most practical applications, approximations are introduced by using model forcefields containing a small finite number of terms to maximize computational efficiency at the expense of sacrificing exactness. As shown in Section 3 below, angle-bending model potentials described in prior literature have either derivative discontinuities or incorrect dynamics when the bond angle reaches a value of π radians (180°). In this article, I introduce a new angle-bending model potential that solves this problem and has continuous derivatives of all orders within the physically accessible region of bond angle values. This reduces model uncertainty and more accurately captures physical dynamics while still requiring relatively few force constants to be linearly optimized.

This article is part of a group of articles on the foundations of force field functional theory (FFFT). A companion article introduced the new SAVESTEPS protocol to optimize forcefield bonded parameters (aka ‘flexibility parameters’) and applied it to a materials dataset containing 116 metal–organic frameworks (MOFs).^45^ That automated protocol used my new ansatz for separating bonded from nonbonded interactions and my new angle-bending model potential.^45^

Note: this article adopts the convention that function arguments are enclosed in square brackets, while parentheses denote multiplication. For example, *y*[*x* + 2] means ‘*y* as a function of (*x* + 2)’ while *y*(*x* + 2) means ‘*y* times (*x* + 2)’.

## 2. Formally exact ansatz separating nonbonded from bonded interactions in a nonreactive forcefield

### 2.1 Foundations

Because an electron's mass is much smaller than an atomic nuclei's mass, electrons typically move much faster than atomic nuclei, and this gives rise to the Born–Oppenheimer approximation in which the electronic motions are approximately equilibrated (approximation # 1) for each geometric arrangement of the material's atomic nuclei.^46^ Within the Born–Oppenheimer approximation, a chemical system's total energy *E*^Born–Oppenheimer^_total_ can be represented as the sum of nuclear kinetic energy and electronic energy, where the ground-state electronic energy *E*^0^_electronic_ is a functional of the chemical geometry:13
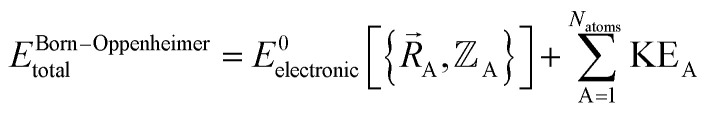
where KE_A_ is the nuclear kinetic energy of atom A. Eqn (13) applies whether or not relativistic corrections are included. Eqn (13) also assumes the electrons occupy the electronic ground state for the chemical geometry defined by 
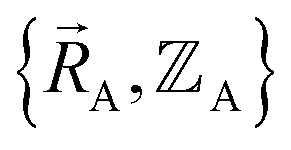
 (approximation # 2). However, eqn (13) allows the atoms to occupy excited and/or ground-state vibrational, rotational, translational states; that is, {KE_A_} can be either ground-state and/or excited-state kinetic energies of the atoms.


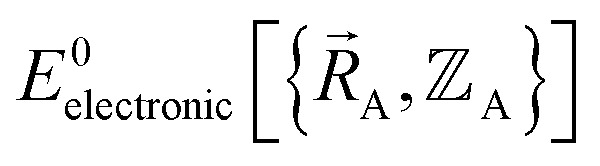
 is the electronic ground-state energy output from an actual quantum chemistry calculation. The exact electronic ground-state energy, 
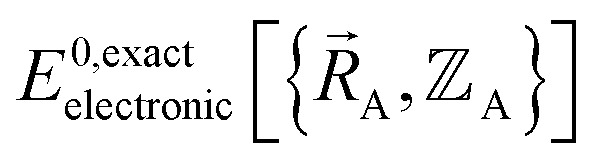
, could conceivably be computed using a full configuration interaction calculation in the complete basis set limit; however, such a calculation may be too computationally expensive in practice.

Within the Born–Oppenheimer approximation, the ground-state electronic energy becomes the potential energy acting on the atomic nuclei.^46^ Accordingly, 
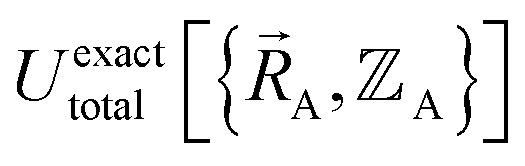
 is the (hypothetical) potential energy functional that would formally reproduce the exact electronic energy exactly:14
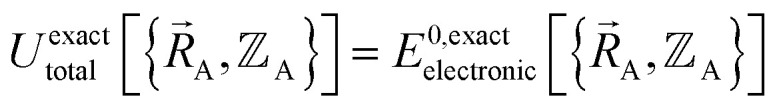
Our goal is to choose a forcefield model that has moderate computational costs and approximately reproduces the exact potential energy functional:15
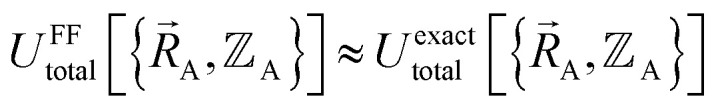
Almost all forcefield models are approximations. A formally exact forcefield corresponds to the (hypothetical) case in which the forcefield's potential energy model is 
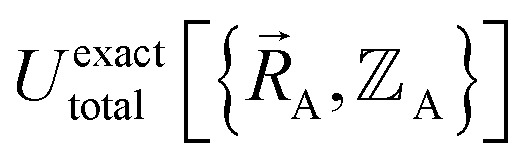
.

The total energy in an atomistic simulation parallels that of eqn (13):16
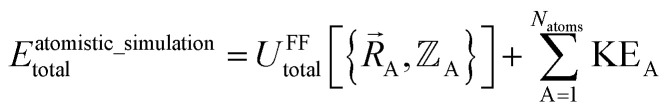


A non-reactive forcefield is limited to describing processes in which no existing chemical bonds are broken and no new chemical bonds are formed (approximation # 3), except that some non-reactive potentials (*e.g.*, Morse potential, QMDFF, *etc.*) can reproduce the energy of bond disassociation as the bond length is infinitely stretched.^47,48^

Although not an approximation of the forcefield itself, nonreactive forcefields are most commonly (but not always) used in simulations employing classical Newtonian mechanics. However, it is also possible to use these same forcefields in simulations involving relativistic mechanics (*e.g.*, special relativity) and/or quantum mechanics (*e.g.*, to describe the tunneling of hydrogen atoms during chemical reactions). For example, such forcefields can be used in Feynman path integral simulations (*i.e.*, path integral molecular dynamics, path integral Monte Carlo).^49,50^

Within a specific individual electronic ground state, continuity of the first derivatives of 
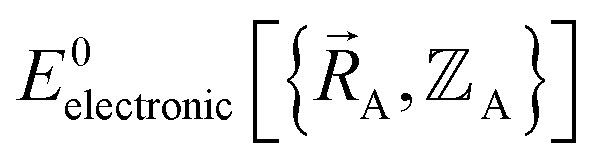
 with respect to changes in the atomic positions 
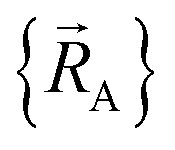
 follows from the Hellmann–Feynman theorem, which states the atom-in-material forces can be computed from the forces the electron cloud exerts on the atomic nuclei.^51^ Although I cannot yet provide a rigorous proof that 
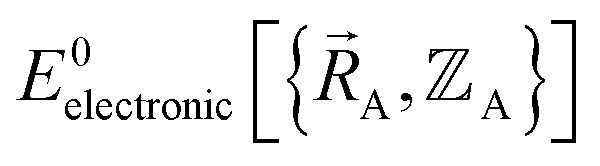
 is continuously differentiable to all orders (*i.e.*, ‘infinitely differentiable’) with respect to changes in the atomic positions 
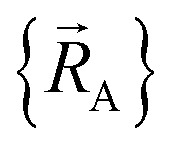
 within a specific individual electronic ground state, this appears to be true if the system Hamiltonian is sufficiently well-behaved. Discontinuities in first (and/or high-order) derivatives of 
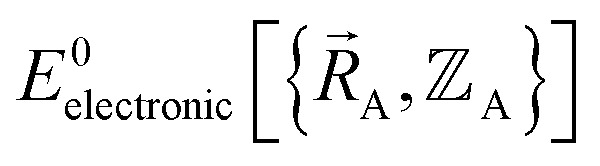
 with respect to changes in the atomic positions 
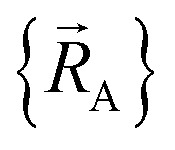
 can arise where the ground state switches from one electronic ground state to another. Example ground state crossovers include singlet-to-triplet ground-state transitions, conducting to semi-conducting transitions, transitions from one magnetic ground state to another, charge-ordering transitions, transitions that change the crystal symmetry, and so forth.^52–55^ Accordingly, 
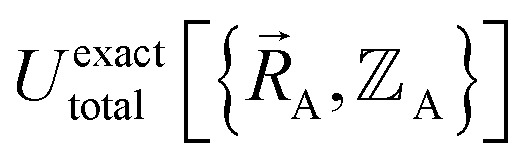
 is conjectured to be continuously differentiable to all orders with respect to changes in the atomic positions 
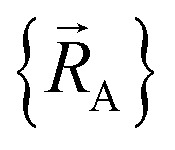
 within each subdomain of 
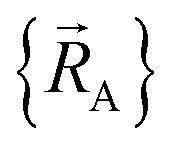
 space that shares the same specific individual electronic ground state, but may exhibit derivative discontinuities at the boundaries where two or more such subdomains intersect. The value of 
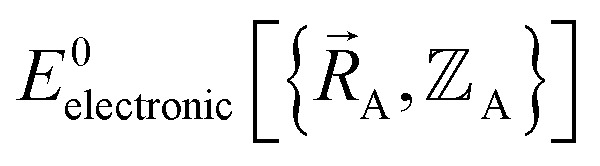
 and hence also of 
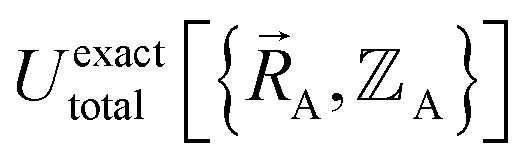
 varies continuously even at boundaries where the ground state switches from one electronic state to another, because the energy is equal for both electronic phases at the ground-state crossover. Therefore, a formally exact theory for 
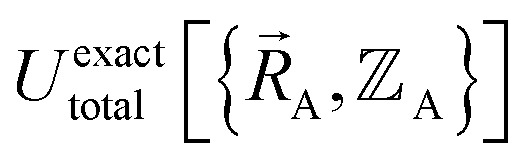
 must be general enough to accommodate such behaviors.

To identify the individual bonded clusters in a simulation, we first construct the bond connectivity graph using atom typing radii.^10^ Two atoms are classified as directly bonded to each other iff the distance between them is no greater than the sum of their atom typing radii.^10^ Two atoms belong to the same bonded cluster iff a connected path of bonds exists between them (for example, if atom A is bonded to B, B is bonded to C, and C is bonded to D, then it follows atoms A and D belong to the same bonded cluster).

Without loss of generality, 
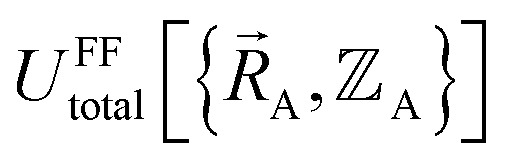
 can be expressed as the sum of interactions occurring solely within each bonded cluster (*i.e.*, intracluster) and those involving any interactions between two or more bonded clusters (*i.e.*, intercluster):17
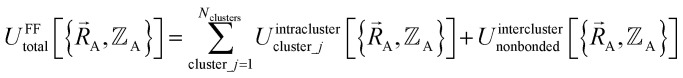
For example, if a 3-body interaction involves two atoms from the same cluster and a third atom from a different cluster, then it is classified as an intercluster rather than an intracluster interaction.

By definition, ‘bonded interactions’ can occur only between atoms in the same bonded cluster. In contrast, ‘nonbonded interactions’ may occur between some atoms in the same bonded cluster and/or between atoms in different clusters. Accordingly, without loss of generality, the bonded and nonbonded interactions are expressed as:18
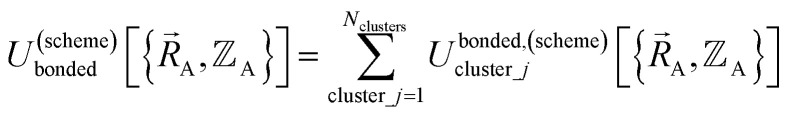
19
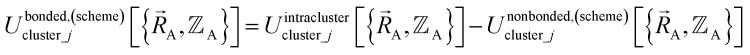
20
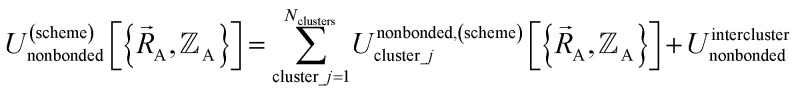


Eqn (19) allows for some flexibility in how we choose to define *U*^nonbonded,(scheme)^_cluster_*j*_ so long as *U*^nonbonded,(scheme)^_cluster_*j*_ and *U*^bonded,(scheme)^_cluster_*j*_ sum to *U*^intracluster^_cluster_*j*_. As derived below, choosing a specific ansatz to define *U*^nonbonded,(scheme)^_cluster_*j*_ is not arbitrary, because some definitions (*e.g.*, the old scheme, eqn (8) and (9)) require nonlinear regression to optimize the forcefield's bonded parameters while the new scheme defined below is strongly preferred because it allows the forcefield's bonded parameters to be optimized using linear regression. Eqn (18)–(20) apply to both the old scheme and the new scheme; however, the definitions for the individual terms appearing in these equations depend on which scheme is chosen.

The exact intracluster force is given by21
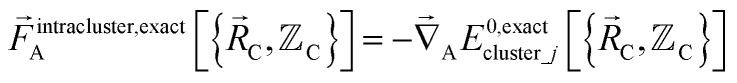
Without loss of generality, the force acting on atom A in the system according to the forcefield model can be expanded as:22
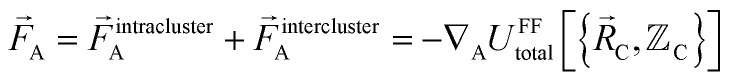
23
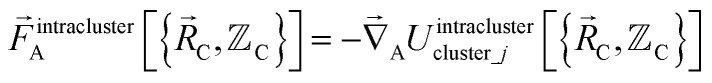
24
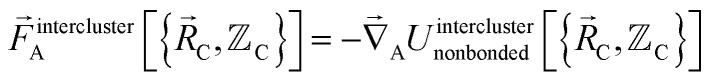
25
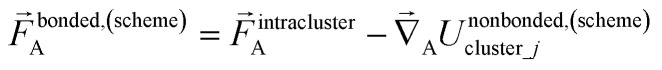
where atom_A ∈ cluster_*j*.

### 2.2 A new ansatz for separating bonded interactions from nonbonded interactions

In the new scheme, the intercluster and intracluster nonbonded interactions are expanded using effective multibody pairwise potentials:26
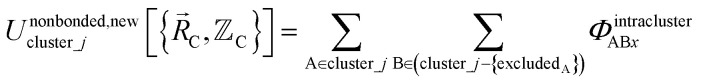
27
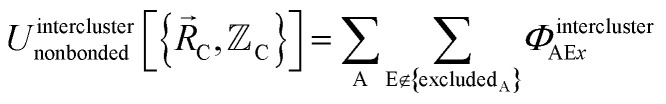
In eqn (26), the summation over B includes all atoms in cluster_*j* except those in {excluded_A_}. In eqn (27), the summation over E includes all atoms in the entire system (whether in cluster_*j* or any other cluster) except those in {excluded_A_}.

Here, the subscript AB*x* refers to the nonbonded potential energy assigned to the atom pair AB. The lowercase *x* indicates this includes the 2-body AB interaction plus the portion of multibody interactions (*i.e.*, *n*-body interactions for *n* ≥ 3) assigned to the AB atom pair. For example, the Axilrod-Teller 3-body dispersion energy^56^ could be divided into equal thirds assigned to the pairs AB*x*, AC*x*, and BC*x*. If atoms A, B, and C belong to the same bonded cluster, then the 3-body ABC interaction is partitioned into contributions that go towards *Φ*^intracluster^_AB*x*_, *Φ*^intracluster^_AC*x*_, and *Φ*^intracluster^_BC*x*_. If atoms A, B, and/or C belong to different bonded clusters, then the 3-body ABC interaction is partitioned into contributions that go towards *Φ*^intercluster^_AB*x*_, *Φ*^intercluster^_AC*x*_, and *Φ*^intercluster^_BC*x*_.

We define 
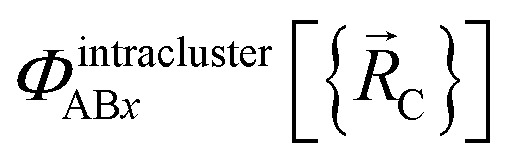
 as the two-body nonbonded interaction energy between atoms A and B belonging to the same bonded cluster plus the portion of intracluster multibody nonbonded interaction energy assigned to the AB pair. If atoms A and D belong to different bonded clusters, then 
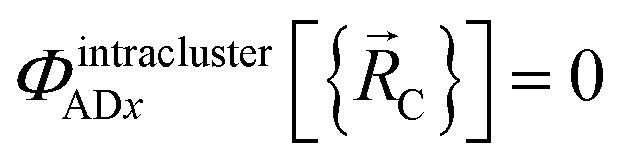
.

In eqn (27), atom E may belong either to the same or a different bonded cluster as atom A. We define 
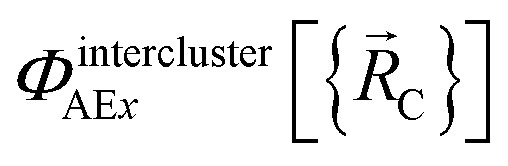
 as the portion of the intercluster nonbonded energy, 
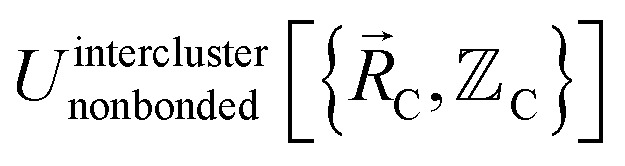
, that is assigned to the AE pair. 
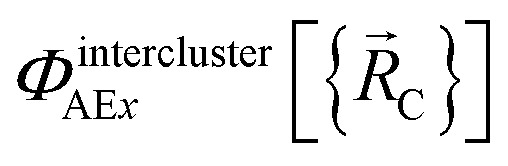
 includes intercluster interaction energies of all orders *n* ≥ 2. For atoms A and B belonging to the same bonded cluster, the two-body (*i.e.*, *n* = 2) AB interaction counts exclusively towards 
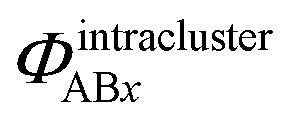
 and not towards *Φ*^intercluster^_AB*x*_. If atoms A and D belong to different bonded clusters, then the two-body (*i.e.*, *n* = 2) AD interaction counts towards *Φ*^intercluster^_AD*x*_.

For example, a system containing three water molecules, one carbon dioxide molecule, plus one MOF has five bonded clusters. In this case, cluster_1 is the first water molecule, cluster_2 is the second water molecule, cluster_3 is the third water molecule, cluster_4 is the carbon dioxide molecule, and cluster_5 is the MOF. In this case, 
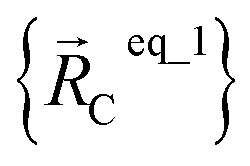
, 
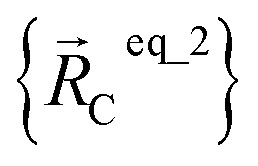
, and 
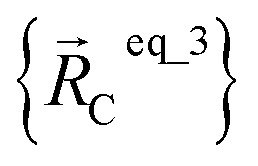
 are the optimized geometry of an isolated water molecule; 
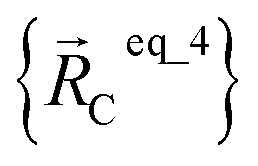
 is the optimized geometry of an isolated carbon dioxide molecule; and 
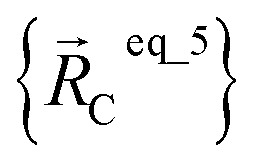
 is the optimized geometry of the bare MOF (*i.e.*, the MOF containing no adsorbate molecules). For crystals, either experimentally-measured or theoretically-computed lattice constants (*i.e.*, unit cell lengths *a*, *b*, *c* and unit cell angles *α*, *β*, *γ*) can be used to define the unit cell's shape and volume when computing 
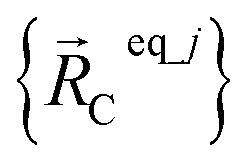
; normally, we use whichever data source is more accurate.

The new scheme is designed so that the zero-, first-, and second-order derivatives of the intracluster nonbonded potential energy are zero by construction within the equilibrium (‘optimized’) ground-state geometry of each isolated bonded cluster *j*:28
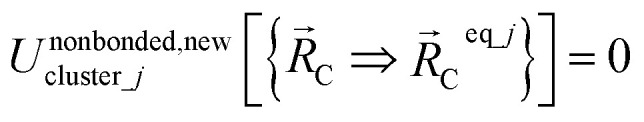
29
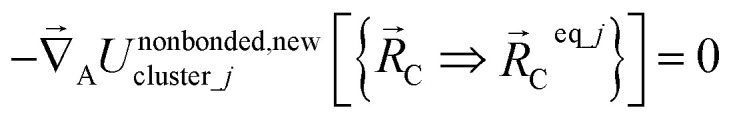
30
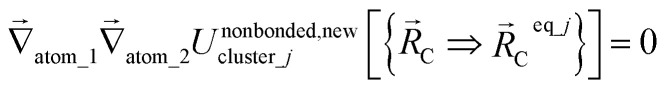
where 
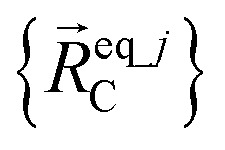
 are the optimized atom-in-material coordinates in the isolated bonded cluster *j*. Here, the notation 
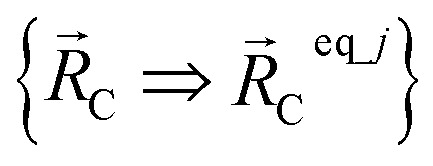
 means the subset of atoms from the full system which are contained within cluster_*j* are positioned where they would be located within the lowest energy configuration of the isolated bonded cluster *j*. 
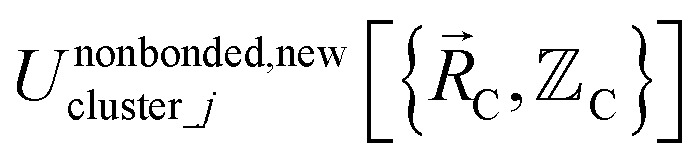
 makes no contribution to (*i.e.*, does not affect) the energy (eqn (28)), atom-in-material forces (eqn (29)), and Hessian matrix (*i.e.*, matrix of second derivatives with respect to atomic displacements, eqn (30)) at the optimized geometry of isolated cluster_*j*.

Many classical molecular dynamics and Monte Carlo software packages have the option to use truncated and shifted nonbonded potentials that go to zero whenever the distance between two atoms A and B is greater than or equal to a nonbonded interaction cutoff distance, *d*^nonbonded^_cutoff_.^42,43^ In general, such a ‘bare’ shifted nonbonded potential has discontinuous forces at the cutoff distance.^43^ To avoid this discontinuity, both the forces and potential can be truncated and shifted at the cutoff distance; however, this still yields discontinuous second derivatives at the cutoff distance.^43^

The new scheme is designed so that the zero-, first-, and second-order derivatives of the potential energy are continuous even at the nonbonded interaction cutoff distance, and this also ensures that the atom-in-material forces and their first-order derivatives are continuous. Specifically, we will design the new scheme so that the following constraints hold for all systems31
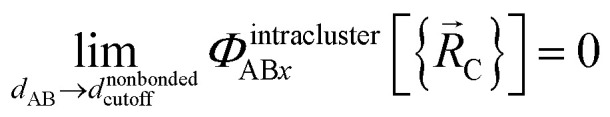
32
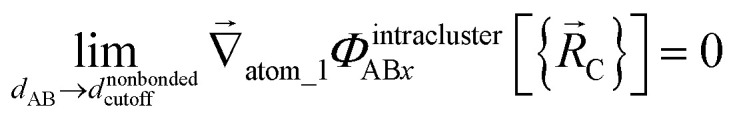
33
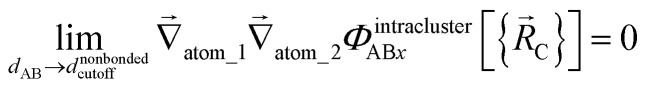
34
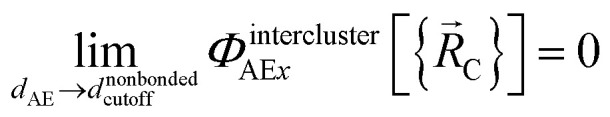
35
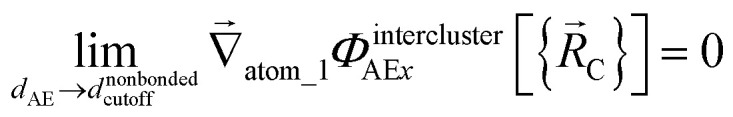
36
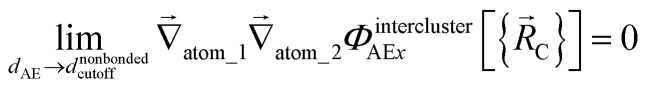
In eqn (32)–(36), atom_1 and atom_2 can be either atom A, B, E, or any other atom. Moreover, atom_1 and atom_2 can be either the same or different atoms.

To accomplish this, we first define a simple parameter-free smooth transition function *τ*_AB_[*s*, *t*] that satisfies the following conditions:

(1) *τ*_AB_[*s*, *t*] should satisfy the bound

37 −1 ≤ *τ*_AB_[*s*, *t*] ≤ 1

(2) *τ*_AB_[*s*, *t*] should smoothly turn on when *s* largely differs from *t*, while remaining mostly turned off when *s* ≈ *t*. Specifically,38
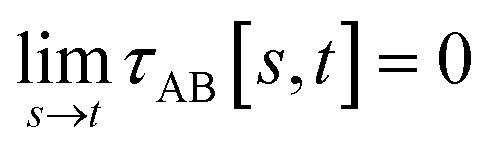
39
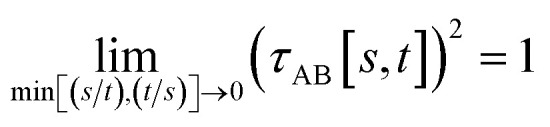


(3) *τ*_AB_[*s*, *t*] should be independent of the choice of measurement units, because its value is a function of the dimensionless ratio *t*/*s*.

(4) *τ*_AB_[*s*, *t*] should increase monotonically as the ratio *t*/*s* increases:40
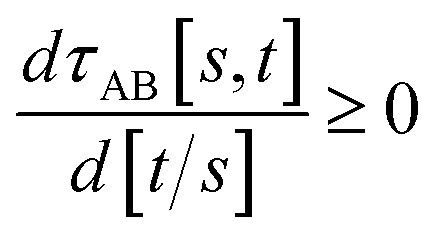


(5) By using various powers of *τ*_AB_[*s*, *t*], the higher-order derivatives expand as follows41
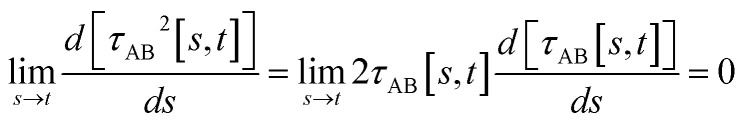
42
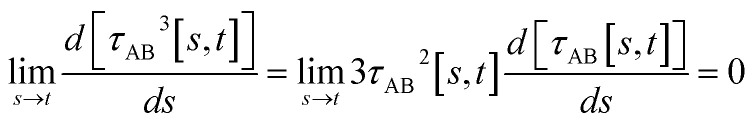
43
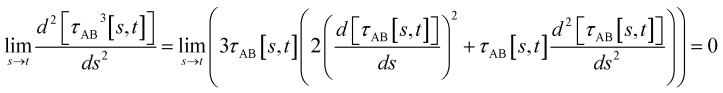
where use has been made of eqn (38).

(6) To achieve a balance between *τ*_AB_ increasing neither too quickly nor too slowly, *τ*_AB_[*s*, *t*] should be mostly turned on (*i.e.*, (*τ*_AB_)^2^ ≈ 0.8) when *t*/*s* = 2.

**Fig. 1:**
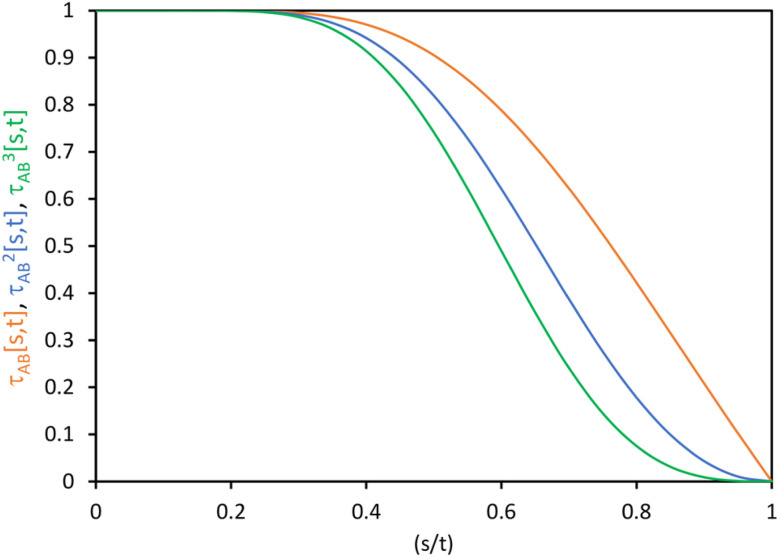
Plot of the smooth transition function *τ*_AB_[*s*, *t*] for the nonbonded potential. The square and cube of this function are also plotted.

The following specific choice of smooth transition function44
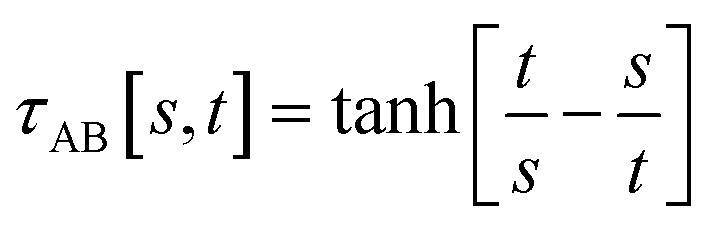
has a simple form satisfying all of the above conditions. When *t*/*s* = 2, *τ*_AB_^2^ [*s*, *t*] = tanh^2^[2 − ½] = 0.819…. This strikes a compromise between *τ*_AB_ increasing neither too quickly nor too slowly as *t*/*s* increases. Fig. 1 plots this smooth transition function.

Using this smooth transition function, we can now arrange the nonbonded interactions according to four cases to satisfy eqn (28)–(36) above. Case # 1: the two atoms A and B are inside the same bonded cluster *j* and a cutoff distance is used for their nonbonded interaction. In this case, we express the effective multibody pairwise potentials as follows:45
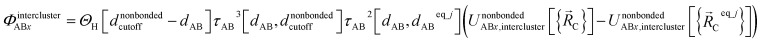
46
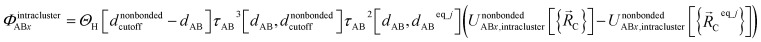
*Θ*_H_ is the Heaviside step function, and *d*_AB_^eq_*j*^ is the equilibrium distance between atoms A and B in the isolated bonded cluster *j*.

Case # 2: the two atoms A and B are inside the same bonded cluster *j* and a cutoff distance is not used for their nonbonded interaction. In this case, we express the effective multibody pairwise potentials as follows:47
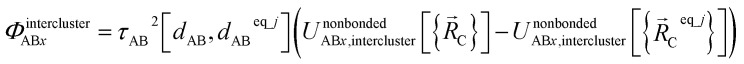
48
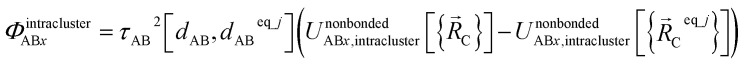


Case # 3: the two atoms A and D are not inside the same bonded cluster and a cutoff distance is used for their nonbonded interaction. In this case, we express the effective multibody pairwise potentials as follows:49
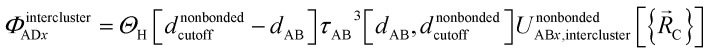

50 *Φ*^intracluster^_AD*x*_ = 0

Case # 4: the two atoms A and D are not inside the same bonded cluster and a cutoff distance is not used for their nonbonded interaction. In this case, we express the effective multibody pairwise potentials as follows:51
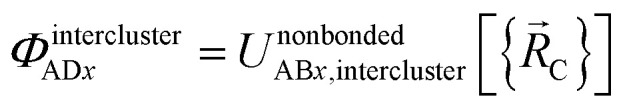

52 *Φ*^intracluster^_AD*x*_ = 0

Analytic first- and second-order derivatives of the nonbonded interactions for these four cases are shown in ESI Section S2.†

If the cutoff distance used for the nonbonded potential is infinite (*i.e.*, *d*^nonbonded^_cutoff_ → ∞), then53
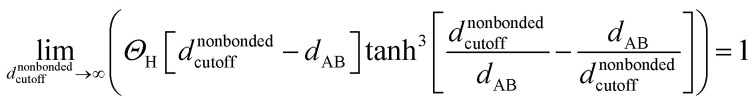
Accordingly, Case # 2 can be regarded as the *d*^nonbonded^_cutoff_ → ∞ limit of Case # 1, and Case # 4 can be regarded as the *d*^nonbonded^_cutoff_ → ∞ limit of Case # 3.

According to these new definitions, *U*^intercluster^_nonbonded_ and *U*^nonbonded,new^_cluster_*j*_ have continuous values and continuous first and second derivatives everywhere with respect to atom displacements. This should provide improved numeric precision when performing classical molecular dynamics and Monte Carlo simulations using a nonbonded interaction cutoff distance.

By convention, the nonbonded interaction energy goes to zero for two atoms infinitely far apart:54
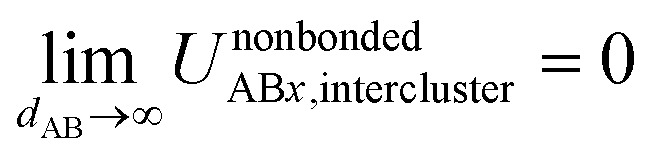
55
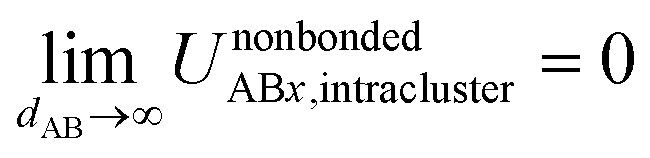


Specific models for *U*^nonbonded^_AB*x*, intercluster_ and *U*^nonbonded^_AB*x*, intracluster_ can include nonbonded interactions due to some or all of the following: atomic charges, atomic dipoles, atomic quadrupoles, atom-in-material polarizabilities, short-range repulsion, long-range dispersion interactions with short-range damping, *etc.*^8–12,14,19,56–63^ A Lennard-Jones plus atomic charges model, 
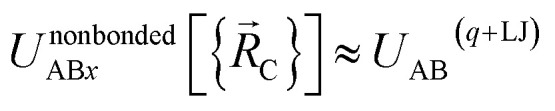
, or more sophisticated (and hopefully more accurate) models could be used for the nonbonded interactions. These are mentioned only as examples, because the possibilities are delineated only by the capacities of human ingenuity.

For an isolated bonded cluster *j*, the force on atom A is56
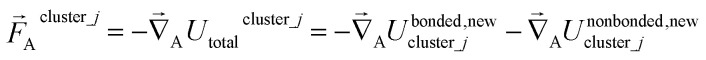
At the optimized geometry of this isolated bonded cluster, the force on each atom in the cluster is zero:57
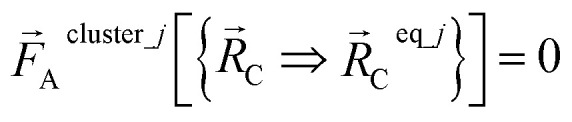
Substituting eqn (29) and (56) into (57) gives58
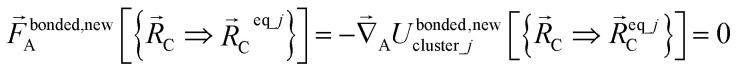


Eqn (58) is the key result that enables the new scheme to directly use the equilibrium geometric parameters from isolated cluster_*j*'s quantum-mechanically-computed optimized ground-state geometry as the ‘resting values’ in the bonded interaction terms. This enables the new scheme to use linear regression instead of requiring nonlinear regression to optimized cluster_*j*'s bonded parameters.

Consider a system that contains only an isolated bonded cluster_*j*. In this case, the quantum-mechanically-computed optimized ground-state geometry 
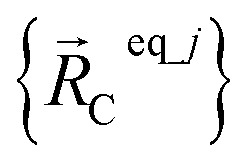
 is always an equilibrium structure of the constructed forcefield; that is, the atom-in-material forces are zero as shown in eqn (57). With proper forcefield parameterization, the quantum-mechanically-computed optimized ground-state geometry 
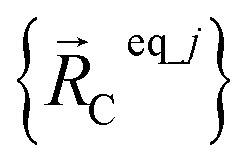
 should preferably be at least a local energy minimum and more preferably a global energy minimum in the forcefield's potential energy surface for this isolated bonded cluster_*j*. In other words, an isolated bonded cluster's (*e.g.*, a molecule's or a MOF's) optimized ground-state geometry can still be predicted exactly by the forcefield even if the forcefield's potential energy function is an approximation! Near 
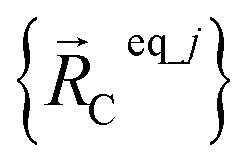
, eqn (28)–(30) show *U*^nonbonded,new^_cluster_*j*_ only affects the third- and higher-order derivatives that control anharmonicity. This enables the forcefield's bonded force constant values (at least within the subdomain containing the cluster's optimized ground-state geometry) to be optimized to good approximation without requiring specific models for 
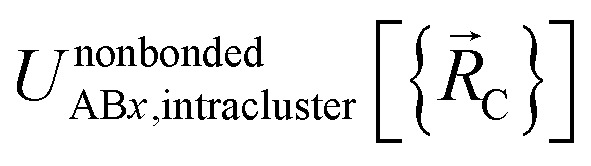
 or 
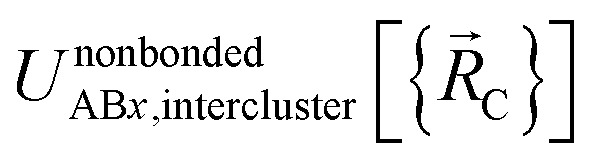
 to be chosen ahead of time. This facilitates directly comparing forcefields using different nonbonded interaction models without having to reoptimize the bonded parameters. (Since it is formally exact under the conditions described in Section 2.5 below, this new scheme can certainly also be used to precisely describe the anharmonicity; however, in that case a change in 
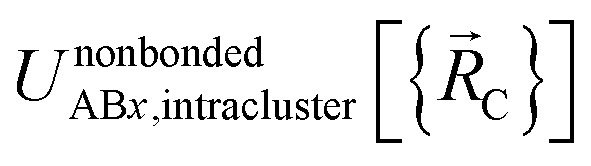
 requires also adjusting the bonded parameters to maintain an accurate description of the anharmonicity.)

The old scheme is more complicated than the new scheme, because the old scheme does not satisfy eqn (58). Consequently, the ‘resting values’ in the bonded interaction terms of the old scheme do not equal the equilibrium geometric parameters from isolated cluster_*j*'s quantum-mechanically-computed optimized ground-state geometry. For example, a bond stretch under the old scheme could be constructed using the MM3 bond stretch potential, but this gives an optimization problem nonlinear in the parameter *d*^resting^_AB_. In stark contrast, the new scheme does not treat *d*^resting^_AB_ as an unknown to solve for during regression, because under the new scheme *d*^resting^_AB_ = *d*_AB_^eq_*j*^ has a known value before regression.

An analogy helps explain relationships between the old scheme and the new scheme. Suppose that we have a system composed of two fruit pies: one cherry pie and one apple pie. Each of these pies is analogous to the energy of a different bonded cluster in our system. Hypothetically, we could cut each pie into several pieces. By itself, this cutting operation does not introduce any approximations. For example, if we cut the cherry pie into two pieces, this does not introduce any approximations, because these two pieces still sum up to the entire pie. We notice that there are different ways we could choose to cut up the cherry pie. For example, we could cut the cherry pie into a left-side piece (called ‘intracluster bonded interactions’) and into a right-side piece (called ‘intracluster nonbonded interactions’). The distinction between the old scheme and the new scheme is that they are different protocols for cutting the cherry pie into pieces. Although this choice affords some flexibility, it is not completely arbitrary, because some protocols (*e.g.*, the new scheme) for separating intracluster bonded interactions from intracluster nonbonded interactions yield a linear regression problem for the bonded parameters while some other separation protocols (*e.g.*, the old scheme) yield a nonlinear regression problem for the bonded parameters. While we get to choose how to cut up the cherry pie into pieces, the physical separation between the cherry pie and the apple pie, which is analogous to the ‘intercluster nonbonded interactions’, is defined by nature rather than being chosen by us. Suppose that we have a system comprised of several bonded clusters. In this case, the ‘intercluster nonbonded potential energy’ is the difference between the Born–Oppenheimer electronic energy of the total system and that of the isolated clusters:59
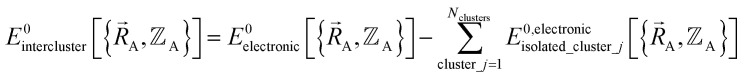
Each term in eqn (59) is physically defined in a non-subjective manner.

### 2.3 Expanding the bonded interactions

Because of eqn (30) and (58), we can construct *U*^bonded,new^_cluster_*j*_ such that leading terms in its series expansion in the vicinity of 
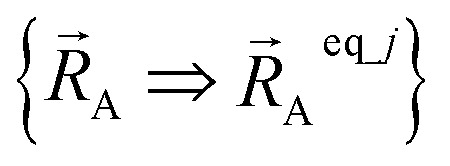
 are second-order in internal coordinate displacements:60
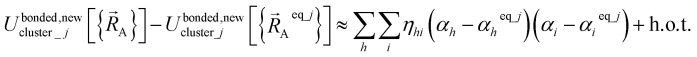
where ‘h.o.t.’ are higher order terms. Here, *α*_*i*_ is an internal coordinate, and *α*_*i*_^eq_*j*^ is its equilibrium value in the quantum-mechanically-computed ground-state geometry of isolated cluster_*j*. *η*_*hi*_ is the corresponding expansion coefficient. Eqn (60) is not a Taylor series expansion, because the set of internal coordinates is partly redundant.

Alternatively, we can expand *U*^bonded,new^_cluster_*j*_ as a linear combination of flexibility terms, such that each flexibility term has a Taylor series expansion whose leading term is second-order in internal coordinate displacements:61
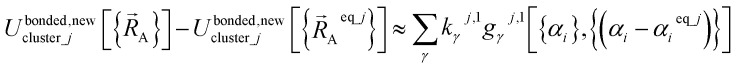
62
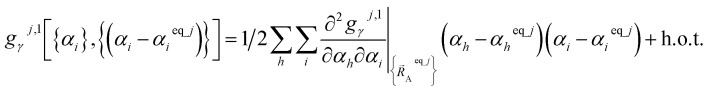
where *k*^*j*^_*γ*_^,1^ is the force constant and ‘h.o.t.’ are higher order terms. Eqn (62) is a Taylor series expansion, because the internal coordinates contributing to a single flexibility term are independent of each other (*i.e.*, non-redundant).

Owing to their continuous differentiability with respect to changes in the internal coordinates, the expansions shown in eqn (61) and (62) are only valid within the subdomain of 
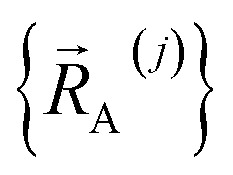
 space that share the same electronic ground state type as 
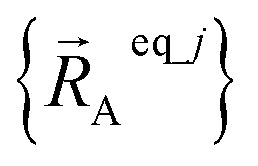
. 
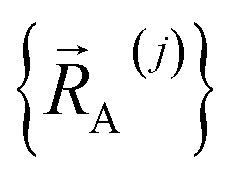
 is the set of atom-in-material coordinates for only those atoms contained in cluster_*j*. We can construct a formally exact expansion of 
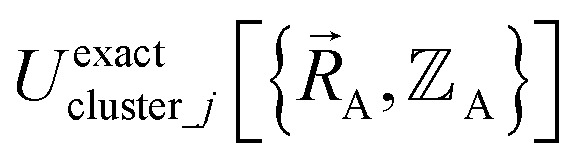
 that is globally valid over all accessible regions of 
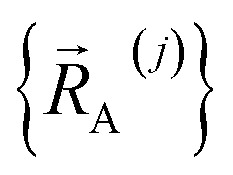
 space by concatenating expansions for the various subdomains describing different electronic ground states:63
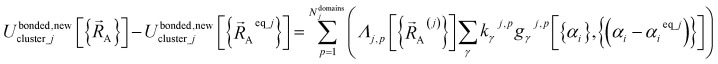
64
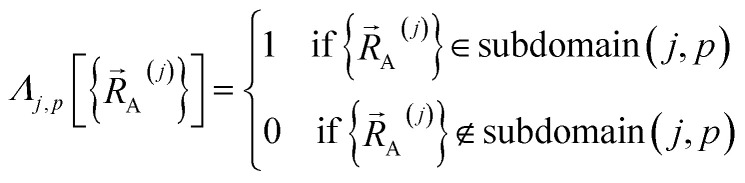


Subdomain (*j*, *p*) means the *p*th electronic ground-state subdomain of cluster_*j*. Each subdomain (*j*, *p*) gets its own internal coordinate expansion and its own force constant values. The subdomains are chosen such that each single geometry, which is defined by 
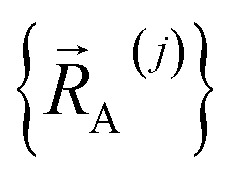
, belongs to exactly one subdomain. Two different geometries of cluster_*j* may belong to the same or different subdomains, but a single geometry of cluster_*j* cannot simultaneously belong to two or more subdomains.

Since the optimized (aka ‘equilibrium’) ground-state geometry which resides in the *p* = 1 subdomain has relative potential and atom-in-material forces equal to zero, its zeroth-order (representing the potential contributions) and first-order (representing the force contributions) terms vanish in the Taylor series expansions of each flexibility term as shown in eqn (62). The *p* ≠ 1 subdomains have no such constraint, because they do not contain the optimized (aka ‘equilibrium’) ground-state geometry. Accordingly, the *p* ≠ 1 subdomains have the following Taylor series expansion:65
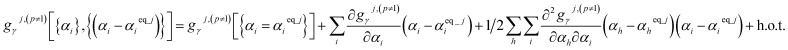


By first choosing various types of flexibility terms (*e.g.*, bond stretches, angle bends, dihedral torsions) as {*g*_*γ*_^*j*,*p*^[{*α*_*i*_}, {(*α*_*i*_ − *α*_*i*_^eq_*j*^)}]}, we clearly have a linear optimization problem whose goal is to find the set of force constants values {*k*_*γ*_^*j*,*p*^} such that 
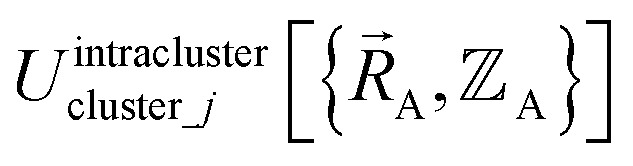
 resembles 
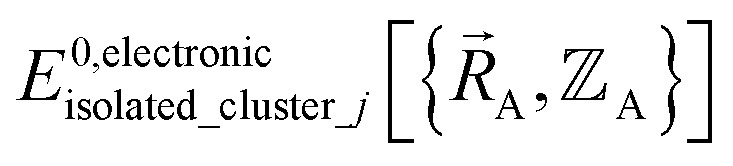
 as closely as feasible subject to some optional constraints on the force constants. For example, we may want to constrain some of the force constants to have non-negative values.

### 2.4 Parameter optimization strategy

The exact bonded force is given by66
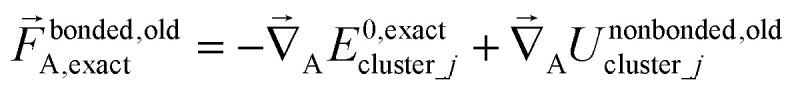
67
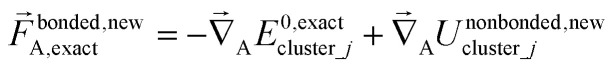


A key distinction between the old and new scheme is that the new scheme obeys eqn (29). Accordingly, 
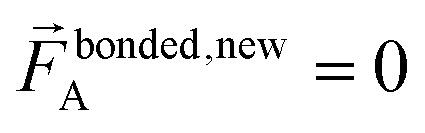
 (eqn (58)) at the equilibrium geometry of the isolated cluster_*j*. This means 
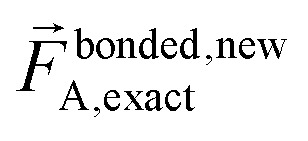
 can be expanded as68
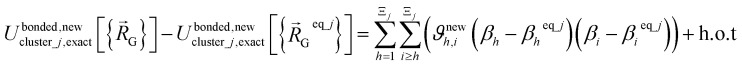
69
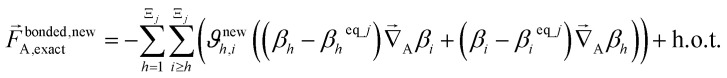
where {*β*_*i*_} is a full set of non-redundant internal coordinates for cluster_*j*. H.o.t. is an abbreviation for higher order terms. {*ϑ*_*h*,*i*_} are the associated constants. Ξ_*j*_ is the total number of non-redundant internal coordinates in cluster_*j*.

In contrast, 
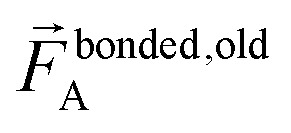
 does not necessarily equal zero at the optimized geometry of isolated cluster_*j*. Consequently, it expands as70
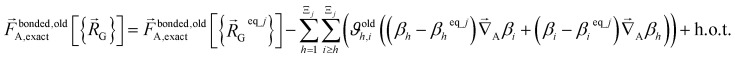
71
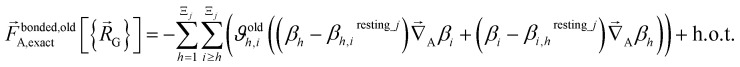
where *β*_*i*,*h*_^resting_*j*^ is usually not equal to *β*_*i*_^eq_*j*^.

For a series of small finite displacements (*e.g.*, 0.0001 Å) away from the equilibrium geometry of isolated cluster_*j*, the intracluster nonbonded force, 
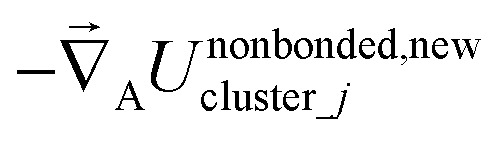
, remains negligible because it is proportional to second-order and higher-order products of the finite displacements. In contrast, the bonded force is proportional to first-order and higher-order products of the finite displacements, as shown in eqn (69). Accordingly, the leading-order harmonic bonded force constants can be optimized by minimizing the following loss function72
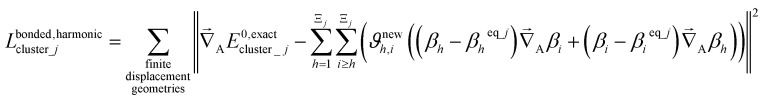


The vector inside ‖.‖ has 3N_atoms_ force components for each geometry. This corresponds to an *x*, *y*, and *z* force component for each atom in the material's unit cell. Minimizing this *L* is a linear least squares optimization problem. Astonishingly, this means the new scheme provides a formally exact method to optimize the leading-order bonded force constants without having to explicitly pick a nonbonded interaction model (*i.e.*, without having to choose a specific model potential for *U*^nonbonded^_AG*x*, intracluster_).

In practice, one often uses a set of redundant (rather than nonredundant) internal coordinates {*α*_*i*_}. In this case, the exact bonded force expands as73
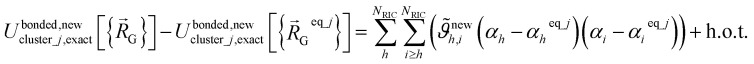
74
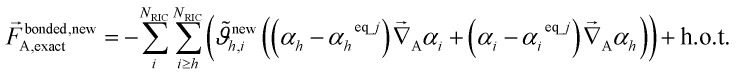
N_RIC_ is the number of redundant internal coordinates in cluster_*j*. This defines a linear least squares problem analogous to eqn (72) except that a regularization method (*e.g.*, LASSO^32,33^) should be used to handle the multicollinearity problem caused by redundancy in the internal coordinates. Again, this allows us to construct and optimize a model for the bonded force constants to leading order without having to explicitly pick a nonbonded interaction model.

As shown in eqn (61) and (62), it is possible to use a set of flexibility terms {*g*^*j*,1^_*γ*_} that have a similar expansion to leading order as eqn (73). This defines a linear least squares problem with possible multicollinearity that should be addressed by using a regularization method (*e.g.*, LASSO^32,33^). Once again, this allows us to construct and optimize a model for the bonded force constants to leading order without having to explicitly pick a nonbonded interaction model. This amazing result is used to optimize bonded interaction models for 116 MOFs in the companion paper.^45^

Notably, the new scheme is formally exact to all orders, not merely to leading order. To compute the formally exact bonded force constants to all higher orders, the new scheme requires the loss function to also include the intracluster nonbonded interactions:75
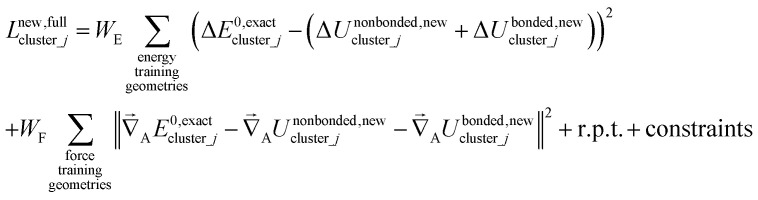

76 Δ*E*^0^_cluster_*j*_ = *E*^0^_cluster_*j*_[{*R*_C_}] − *E*^0^_cluster_*j*_[{*R*_C_^eq_*j*^}]

77 Δ*U*^nonbonded,(scheme)^_cluster_*j*_ = *U*^nonbonded,(scheme)^_cluster_*j*_[{*R*_C_}] − *U*^nonbonded,(scheme)^_cluster_*j*_[{*R*_C_^eq_*j*^}]

78 Δ*U*^bonded,(scheme)^_cluster_*j*_ = *U*^bonded,(scheme)^_cluster_*j*_[{*R*_C_}] − *U*^bonded,(scheme)^_cluster_*j*_[{*R*_C_^eq_*j*^}]

where r.p.t. is the regularization penalty term that handles the multicollinearity problem. Here, *W*_E_ and *W*_F_ are observation weights applied to the energies and forces, respectively. A full series expansion (*e.g.*, eqn (63)) for *U*^bonded,new^_cluster_*j*_ must be inserted into eqn (75). In practical applications, the following leading-order approximation is typically used79
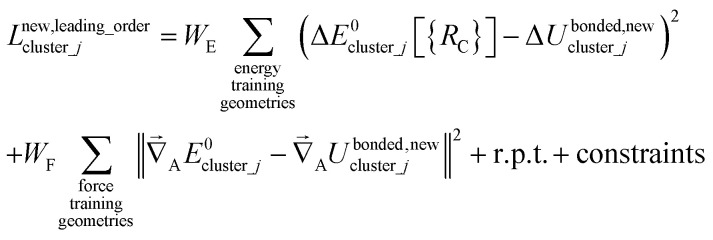
where one uses an approximate (*i.e.*, truncated) series expansion for *U*^bonded,new^_cluster_*j*_.

The old scheme explicitly requires us to specify a particular nonbonded interaction model even if we only want to optimize the bonded force constants to leading order. Although this type of regularization has not been used with the old scheme in the prior literature,^17,22,23,27,64,65^ one could construct the following type of loss function for the old scheme80
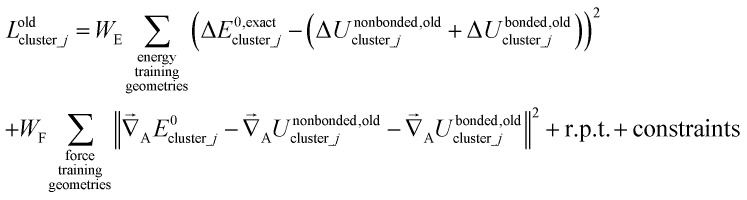


Compared to the new scheme, the old scheme requires additional terms and/or additional parameters to expand the bonded potential, because under the old scheme the bonded forces are not necessarily zero at isolated cluster_*j*'s optimized geometry. The following two requirements of the old scheme make it much more complicated than the new scheme:

(i) The old scheme requires explicitly choosing and including *U*^nonbonded,old^_cluster_*j*_ even if we only want to optimize the bonded force constants to leading order. Under the old scheme, even the leading-order bonded force constant values depend on the particular choice of intracluster nonbonded potential model, *U*^nonbonded,old^_cluster_*j*_.

(ii) The old scheme requires optimizing more bonded parameters than the new scheme. Specifically, the old scheme requires optimizing ‘resting values’ in the flexibility terms or including non-zero force intercept terms in the flexibility model.

In summary, this new scheme for separating bonded from nonbonded interactions in a nonreactive forcefield has so many compelling advantages that it should completely replace the old scheme. It is one of those cases where an important simplification (*i.e.*, turning a nonlinear optimization problem into a linear optimization problem for the bonded parameters) maintains formal exactness and simultaneously improves numeric precision and computational convenience (by providing continuous first- and second-order derivatives when using a nonbonded interaction cutoff distance), transferability (because the choice of 
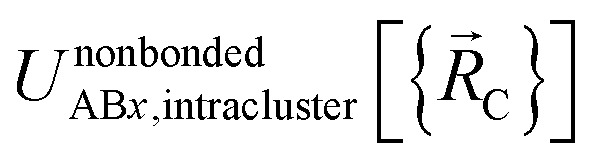
 does not affect forces or Hessian at the isolated cluster's optimized geometry), and convergence robustness (because linear optimization problems do not have multiple local minima in the loss function's value).

**Fig. 2:**
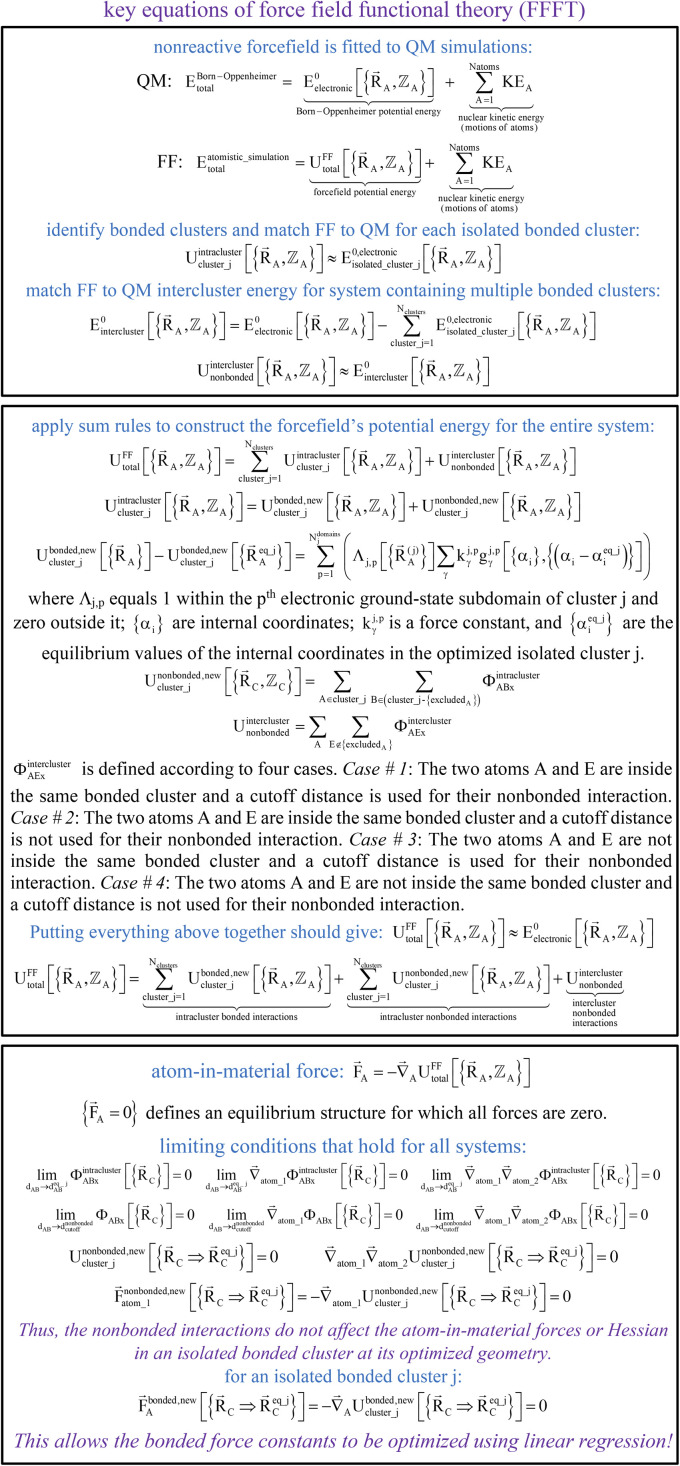
A graphic summarizing relationships between key equations in force field functional theory.

Fig. 2 summarizes the key equations for my new forcefield parameterization process that consists of the following steps:

(1) First, we separate 
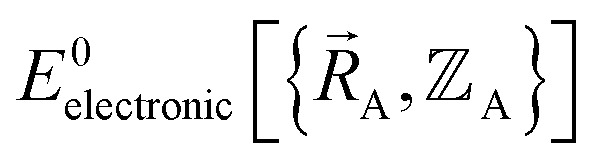
 into intercluster and intracluster contributions. To do this, a series of quantum chemistry calculations are first performed to compute 
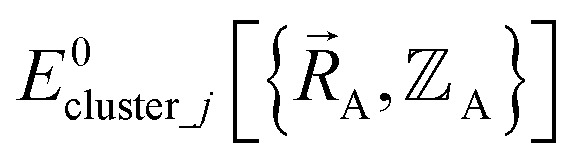
 for each individual bonded cluster_*j* by itself (*i.e.*, an isolated bonded cluster) over many geometries 
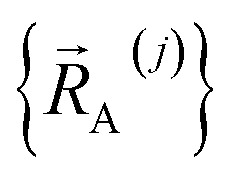
 allowing its internal coordinates (*e.g.*, bond lengths, bond angles, dihedrals, *etc.*) to vary.

(2) Second, a separate set of quantum chemistry calculations is then performed for the entire system that contains all of the bonded clusters together at various geometries. As shown in eqn (59), the intercluster energy is computed as the difference between the Born–Oppenheimer electronic energy of the total system and that of the isolated clusters.

(3) Following the method described in Section 2.2 above, intracluster nonbonded interactions are defined for each isolated bonded cluster. The remaining intracluster energy is assigned to the bonded interactions:81
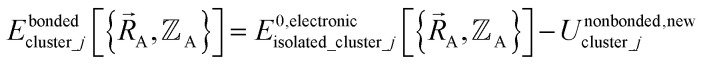


(4) Using linear regression with an appropriate Lagrangian (*L*^bonded^_cluster_*j*_), the intracluster bonded energy model, 
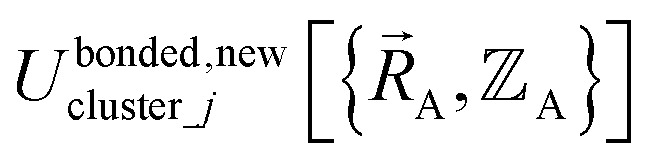
, is fit to an internal coordinate series expansion for each electronic subdomain of cluster *j* to reproduce 
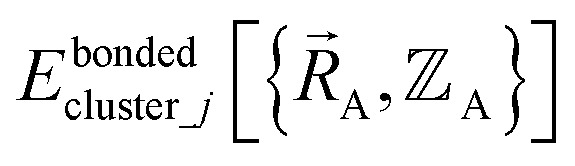
 as closely as feasible:82
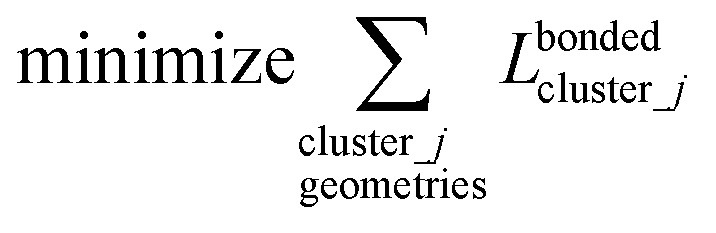


If the internal coordinate series expansion is complete, the minimum of the loss function will be zero, and this corresponds to an exact match between 
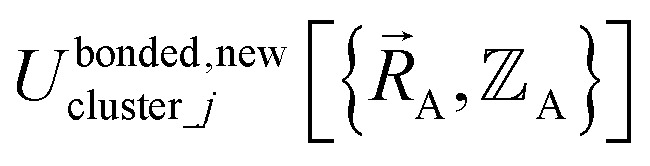
 and 
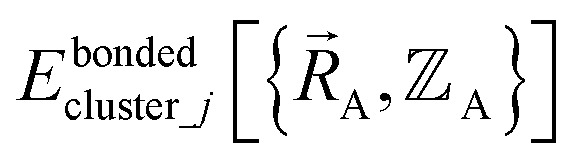
. In most practical applications, a truncated series expansion is used leading to approximation. In addition to energies, the training dataset and Lagrangian for bonded interactions may also include atom-in-material forces and/or constraints (such as bounds on some force constants) and/or regularization terms.

(5) Using linear or nonlinear regression with an appropriate Lagrangian (*L*^intercluster^_nonbonded_), the intercluster nonbonded energy model, 
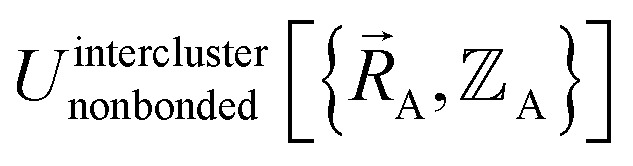
, is fit to an internal coordinate series expansion to reproduce 
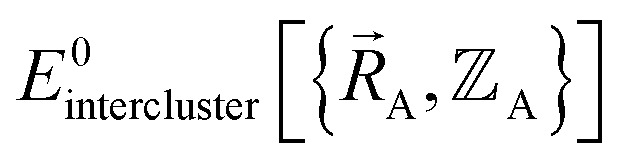
 as closely as feasible by finding the minimum of *L*^intercluster^_nonbonded_. If the internal coordinate series expansion for 
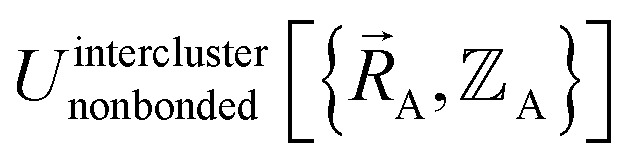
 is complete, the minimum of the loss function will be zero, and this corresponds to an exact match between 
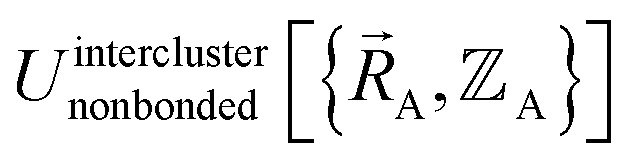
 and 
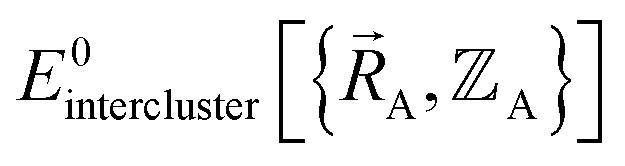
. In most practical applications, a truncated series expansion is used leading to approximation. In addition to energies, the training dataset and loss function for nonbonded interactions may also include atom-in-material forces and/or constraints (such as bounds on some nonbonded parameters) and/or regularization terms. For example, this Lagrangian might take the form83
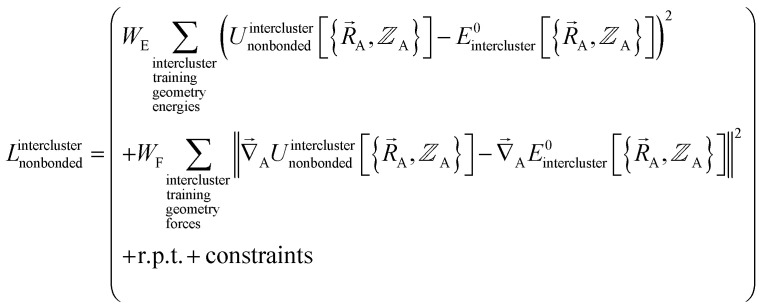


(6) Conceptually, the forcefield's total potential energy can be reconstructed as the sum of three parts, which are the intracluster bonded interactions, the intracluster nonbonded interactions, and the intercluster nonbonded interactions:84
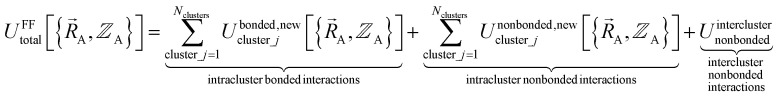


### 2.5 Conditions under which this theoretical framework is formally exact

This section describes the conditions under which the theoretical framework described in the previous sections is formally exact, which means the forcefield's potential energy model asymptotically approaches the exact potential energy model:85
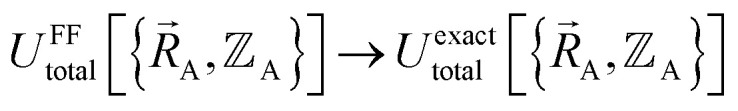


At first one may wonder whether formal exactness is a meaningless theoretical result, because in most practical situations some optional approximations will be chosen to make the forcefield easier to parameterize and use at the expense of losing some accuracy. However, closer analysis shows formal exactness is an extremely important property. If a theoretical framework is not formally exact, then the exact solution lies outside that theoretical framework; in this case, one reaches a wall beyond which the results cannot be further improved without leaving that particular theoretical framework. If a theoretical framework is formally exact, then the exact solution lies inside that theoretical framework; in this case, one can always improve the accuracy of solutions to get closer to and even reach the exact solution without having to leave that particular theoretical framework.

To begin, we must precisely define the problem statement whose exact solution defines the exact solution we seek. Here, the problem statement is defined as follows. For a specific material (aka ‘chemical system’) of precisely defined chemical composition and with precisely defined bond connectivity graph in the absence of externally applied fields, compute the exact Born–Oppenheimer ground-state electronic energy 
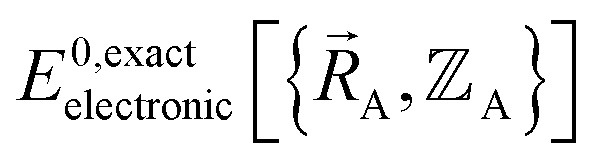
 for various sets of chemical geometries 
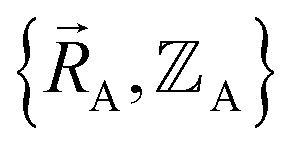
 that match the defined bond connectivity graph, and use these results to construct the exact functional 
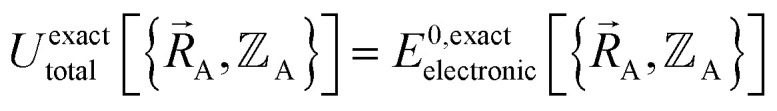
.

The proof that such an exact functional 
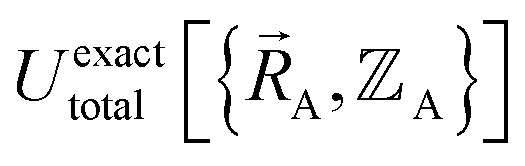
 exists proceeds as follows. In the absence of externally applied fields (*e.g.*, in the absence of externally applied electric, magnetic, and gravitational fields), 
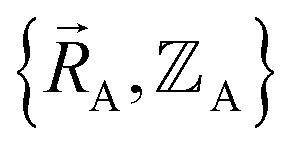
 together with the chosen level of relativistic corrections defines the system's Hamiltonian. The system's Hamiltonian in turn defines its Born–Oppenheimer ground-state electronic energy, 
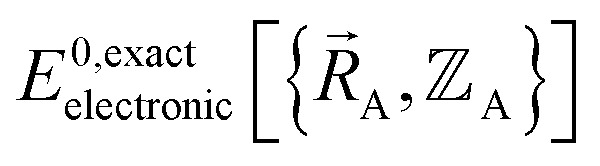
. Since 
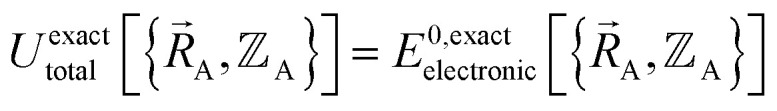
, the existence of 
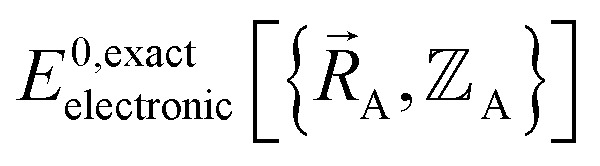
 means 
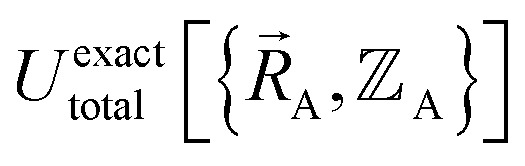
 (which is 
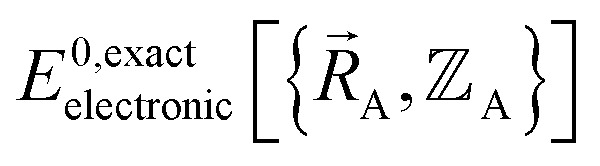
) also exists. Hence, the exact 
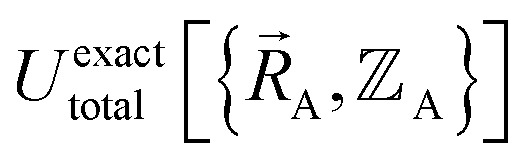
 exists.

Let us examine the hypothetical counter-argument that no 
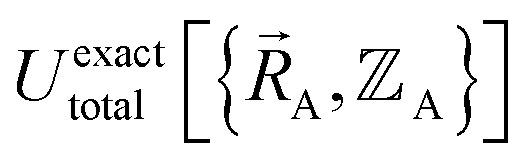
 exists or that it exists but does not equal 
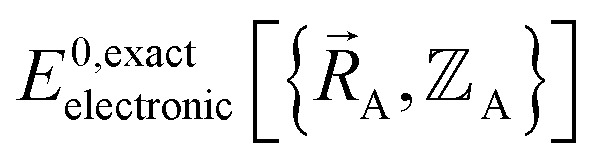
, because the Born–Oppenheimer approximation is itself an approximation. This notion of non-exactness arises from choosing to define the problem statement as *U*^exact^_total_ being intended to match some experimental observable, and since the Born–Oppenheimer approximation is an approximation it does not exactly reproduce experimental observables. While it may be possible to redefine *U*^exact^_total_ in that way, I have chosen not to do so. The concept of using a forcefield's potential energy 
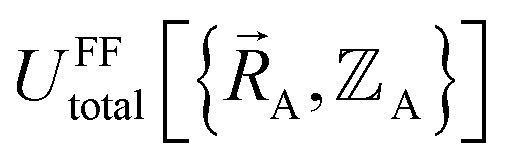
 intrinsically rests on the separation of electronic from nuclear motions. If these are strongly coupled so that the Born–Oppenheimer approximation is unreasonable, then in that case the forcefield's potential energy would need to include the electronic positions 
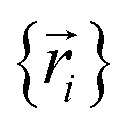
 as well, 
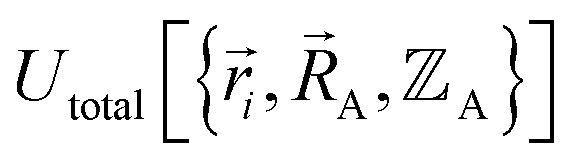
, and both the electrons and atomic nuclei would need to be included as explicit particles when using that forcefield in subsequent simulations. The fact that 
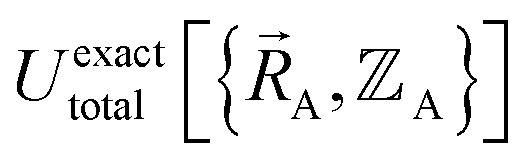
 omits the electronic positions 
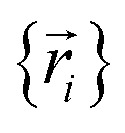
 as explicit coordinates means it only applies within the Born–Oppenheimer approximation that allows the electronic relaxation to be performed for fixed nuclear positions. Hence, 
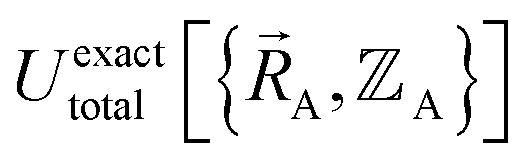
 must be defined as equal to 
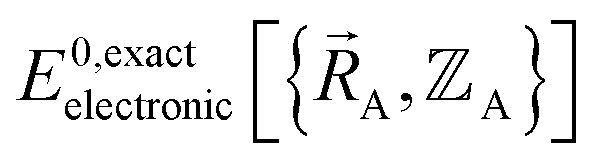
. If we want something that goes beyond the Born–Oppenheimer approximation, that requires a different type of construct, 
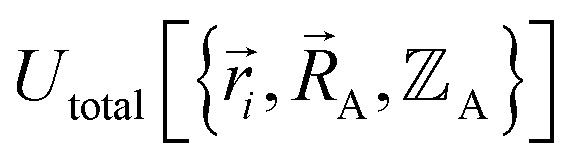
, and a completely different type of forcefield that includes both the electrons and atomic nuclei as explicit particles in the forcefield. Here, I have chosen to define force field functional theory within the scope of the Born–Oppenheimer approximation. Within that defined scope, formal exactness is defined as constructing a forcefield model that exactly reproduces the Born–Oppenheimer potential energy surface as shown in eqn (14) and (85).

Table 1 lists the conditions that must be satisfied for this theoretical framework to reach the exact solution. Each of these conditions is now discussed.

**Table 1:** List of conditions that must be met for this theoretical framework to be formally exact

(1) The bonded clusters and overall system being studied must be exactly the same ones as the forcefield was trained for
(2) No nuclear reactions, no nuclear decay processes, and no nuclear excitation processes take place
(3) Since the nuclear spin and local rotational orientation of an atomic nucleus is neglected in this type of nonreactive forcefield, this type of nonreactive forcefield is formally exact only when each atomic nucleus in the real physical system is spinless and spherically symmetric
(4) There are no externally applied fields, or the forcefield has been specifically parameterized for the precise configuration of externally applied fields that is present
(5) Within the Born–Oppenheimer approximation, an exact electronic structure theory is used to compute 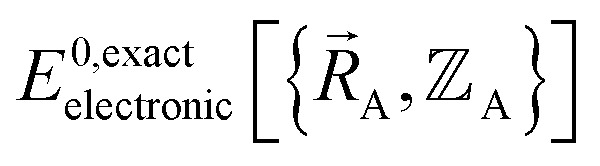 . This requires using an exact exchange–correlation theory together with appropriate relativistic corrections
(6) No new chemical bonds are formed and no chemical bonds are completely severed; however, the bond length of a bond may approach infinity
(7) For nonbonded interactions having theoretically infinite distance range, the *d*^nonbonded^_cutoff_ → ∞ limit must be used. For nonbonded interactions having theoretically limited distance range, the exact range *d*^nonbonded^_cutoff_ = *d*^exact_range^_cutoff_ must be used
(8) Because the forcefield is parameterized for the electronic ground state only, it does not describe processes involving excited electronic states or excited spin states
(9) The forcefield must be used only within the particular electronic ground-state subdomains for which it was parameterized. Since each electronic ground-state subdomain defines a region of atom-in-material positions, 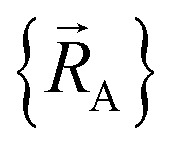 , this type of nonreactive forcefield must be used only within the general regions of 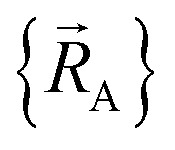 space for which it was trained
(10) 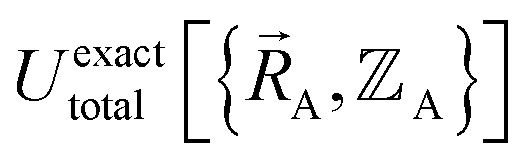 has been constructed to exactly match 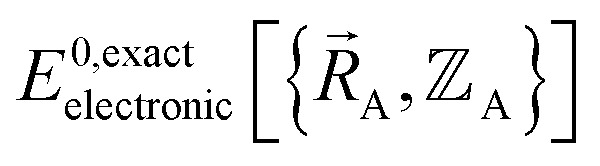 over the relevant electronic ground-state subdomains

To be exact, the bonded clusters and overall system being studied must be exactly the same ones as the forcefield was trained for. Within this theoretical framework, bonded interactions are parameterized for each isolated bonded cluster, and each bonded interaction is intracluster. On the other hand, there are both intracluster nonbonded interactions and intercluster nonbonded interactions. For example, if the forcefield was trained on a system containing three water molecules, then formal exactness is lost and approximations manifest if we try to apply that same forcefield to a new system containing four water molecules. In this case, system # 1 containing 3 water molecules properly has exactly the same bonded interactions and intracluster nonbonded interactions as system # 2 containing 4 water molecules; however, these two systems have different intercluster nonbonded interactions. In other words, the bonded interactions and intracluster nonbonded interactions are strictly transferable between different systems comprised of the same kinds of bonded clusters, but the intercluster nonbonded interactions are not strictly transferable across such systems.

In practice, it is often more convenient to accept some level of approximation that results from applying a versatile intercluster nonbonded interaction model to different but similar systems. Consider as an example a series of systems in which a particular MOF is loaded with different combinations of molecules such as N_2_, O_2_, CO_2_, methane, *etc.* In this case, a forcefield could be developed as follows. First, the bonded interactions and intracluster nonbonded interactions are calculated for each isolated bonded cluster. These are exactly transferable to the combined system containing several bonded clusters. Then, a versatile (but approximate) intercluster nonbonded interaction model is parameterized and applied to each system in the series. This is generally more convenient than the formally exact approach that requires separately parameterizing a new intercluster nonbonded interaction model for each specific combination of molecules in the MOF.

This type of nonreactive forcefield treats the atomic nuclei as immutable. Consequently, this type of nonreactive forcefield describes processes in which no nuclear reactions, no nuclear decay processes, and no nuclear excitation processes take place. Since the nuclear spin and local rotational orientation of an atomic nucleus is neglected in this type of nonreactive forcefield, this type of nonreactive forcefield is formally exact only when each atomic nucleus in the real physical system is spinless and spherically symmetric. In other words, this type of nonreactive forcefield does not describe nuclear magnetic resonance (NMR), the Mössbauer effect, and other phenomena related to nuclear spins or nuclear energy transitions. In line with the Born–Oppenheimer approximation that separates the electronic and nuclear motions, this immutability of atomic nuclei is considered to be part of the problem statement whose formally exact solution is sought.

A formally exact parameterization of the forcefield corresponds to one specific Hamiltonian. Consequently, there is a one-to-one correspondence between the system's Hamiltonian and the forcefield. Any modification that alters the system's Hamiltonian requires parameterizing a new forcefield to retain formal exactness. Since adding external fields (*e.g.*, external electric, magnetic, or gravitational fields) changes the system's Hamiltonian, to retain formal exactness a new forcefield would have to be parameterized for each combination of externally applied fields. Because reparametrizing the forcefield for each specific combination of externally applied fields would be extremely tedious, it is generally more convenient to accept some level of approximation that allows the same forcefield to be applied irrespective of the specific combination of externally applied fields. In general, polarizable forcefields can more accurately approximate responses to externally applied electric fields than nonpolarizable forcefields.^13,19,66–68^

Formal exactness requires that the Born–Oppenheimer electronic ground-state energy, 
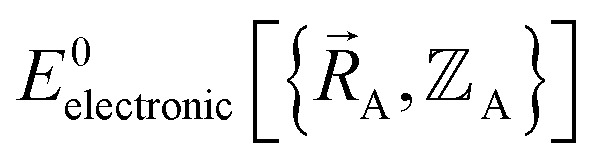
, be computed using an exact quantum chemistry method. This requires both that the exchange–correlation theory used is exact and that appropriate relativistic corrections^69–73^ are included in the quantum chemistry calculations. Examples of formally exact quantum chemistry methods include full configuration interaction expansion and density functional theory (DFT) calculations in the complete basis set limit.^74–77^ Since the exact DFT exchange–correlation functional is still unknown, in practice 
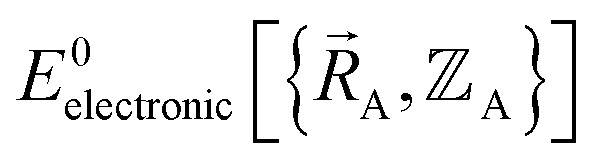
 is normally computed using a density functional approximation (DFA) or any other desired quantum chemistry method (*e.g.*, coupled-cluster, configuration interaction, *etc.*).^77–85^ For best results, the quantum chemistry method chosen should include long-range dispersion interactions.^86–89^ For convenience, a finite-sized basis set is normally used instead of the complete basis set limit.^90–92^ If the finite-sized basis set is appropriately chosen, then this introduces an acceptable level of approximation. For heavy chemical elements, additional approximations such as freezing some of the core electrons or replacing some of the core electrons with a relativistic effective core potential (RECP) are sometimes used.^72,93,94^ Even though the exact DFT exchange–correlation functional is still unknown, all of these approximations are formally optional, because we could conceivably (but not necessarily practically) choose to perform a full configuration interaction calculation in the complete basis set limit to obtain the exact quantum chemistry result; however, that would be extremely (and sometimes prohibitively) computationally expensive.

Each nonreactive forcefield operates only within the scope of a particular fixed bond connectivity graph; however, bonds (treated as harmonic or anharmonic springs) are allowed to stretch beyond the sum of their atom typing radii. Some non-reactive forcefields (*e.g.*, Morse potential,^48^ QMDFF^47^) even allow bonds to stretch to infinite length. Accordingly, nonreactive forcefields do not describe complex chemical reactions. I classify this as a restriction on the scope of nonreactive forcefields, rather than treating it as an approximation. For a collection of atoms, a complete Born–Oppenheimer potential energy surface may contain separate regions (aka ‘valleys’) for reactants and products that are connected by reaction paths. Traversing these reaction paths involves forming and/or breaking chemical bonds. For example, a complete Born–Oppenheimer potential energy surface for four hydrogen, one carbon, and four oxygen atoms would contain separate regions and connecting reaction paths corresponding to: (a) one methane (CH_4_) and two oxygen (O_2_) molecules, (b) one carbon dioxide (CO_2_) and two water (H_2_O) molecules, (c) one formaldehyde (CH_2_O) plus one water (H_2_O) plus one oxygen (O_2_) molecule, and (d) many other regions and connecting reaction paths.

When using a nonbonded interaction cutoff distance, interactions having a theoretically infinite distance range (such as the Coulomb interaction between charged particles) will be undercounted between particles farther apart than *d*^nonbonded^_cutoff_. For nonbonded interactions that have theoretically infinite distance range, formal exactness requires that we use the *d*^nonbonded^_cutoff_ → ∞ limit for those interactions. We also allow the possibility that some (but not all) of the nonbonded interactions may have a theoretically limited distance range. For those particular nonbonded interactions, we should set *d*^nonbonded^_cutoff_ = *d*^exact_range^_cutoff_ (This possibility is included to accommodate multibody interaction models in fluids that have a finite-range of the multibody interactions. In such case, *d*^nonbonded^_cutoff_ = *d*^exact_range^_cutoff_ could be tuned so that the multibody interaction model reproduces the correct interaction energy.). This means the value of *d*^nonbonded^_cutoff_ can be different for different nonbonded interactions.

Because this type of nonreactive forcefield is parameterized for the electronic ground state only, it does not describe processes involving excited electronic states or excited spin states. I classify this as a restriction on the forcefield's scope, rather than treating it as an approximation. Manifestly, this type of nonreactive forcefield cannot describe optical transitions, fluorescence, phosphorescence, photoelectronic processes, electron excitation, electron transport, spin excitation, and spin transport phenomena.

To achieve formal exactness, this type of nonreactive forcefield must be used only within the particular electronic ground-state subdomains for which it was parameterized. It is possible to simultaneously parameterize this type of nonreactive forcefield for one, two, or more different electronic ground-state subdomains. Since each electronic ground-state subdomain defines a region of atom-in-material positions, 
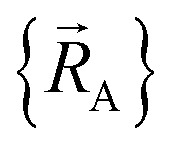
, this type of nonreactive forcefield must be used only within the general regions of 
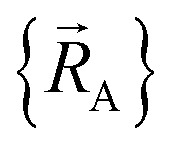
 space for which it was trained.

Finally, to achieve formal exactness, 
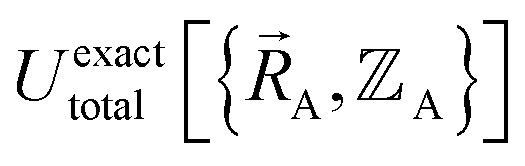
 must be constructed to exactly match 
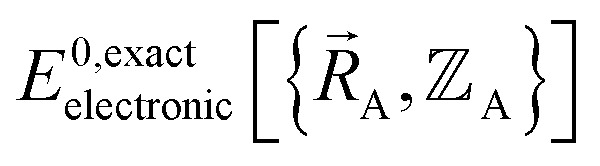
 over the relevant electronic ground-state subdomains. To accomplish this, the process illustrated in Fig. 2 and described in the previous sections should be followed employing complete series expansions in terms of the internal coordinates for both the intracluster bonded interactions and the intercluster nonbonded interactions within each electronic ground-state subdomain. In this context, a ‘complete series expansion’ means a series expansion that employs all the independent degrees of freedom and also has enough functional representation (*i.e.*, a complete set of basis functions) to achieve an exact match between 
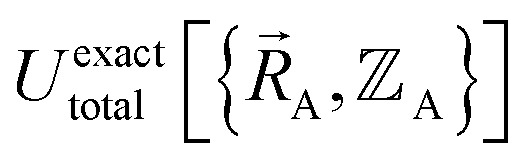
 and 
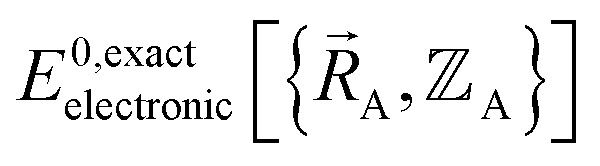
 within each electronic ground-state subdomain.

Both the electronic ground-state subdomains of the full system (which may contain multiple bonded clusters) and the electronic ground-state subdomains of each associated isolated bonded cluster must be included to construct an exact forcefield. Derivative discontinuities in 
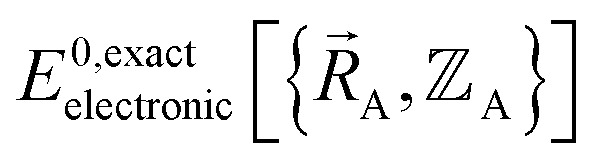
 and hence also in 
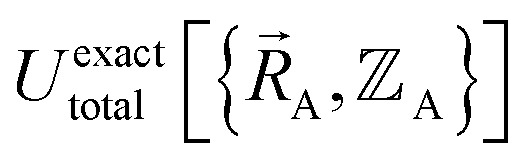
 may occur at a boundary between two or more electronic ground-state subdomains of the full system. 
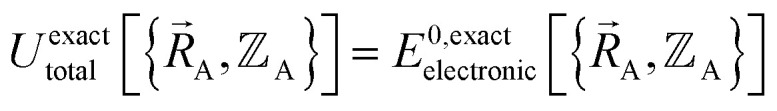
 is continuous but not necessarily continuously differentiable at such boundaries. Derivative discontinuities in 
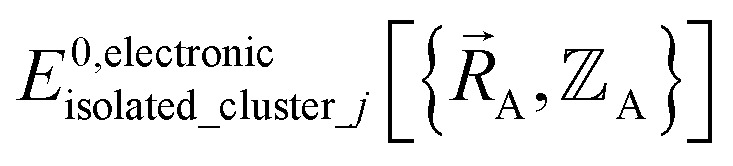
 and hence also in 
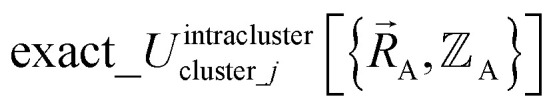
 may occur at a boundary between two or more electronic ground-state subdomains of isolated cluster_*j*. 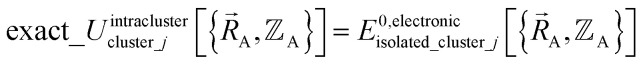 is continuous but not necessarily continuously differentiable at such boundaries. To be exact, 
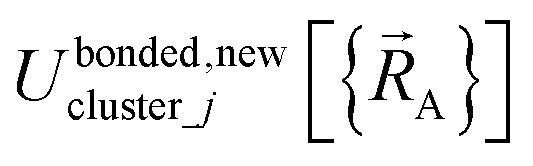
 must be expanded locally within each electronic ground-state subdomain of the isolated cluster_*j* (see eqn (63)). Because 
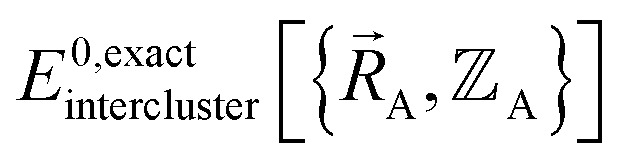
 depends on both 
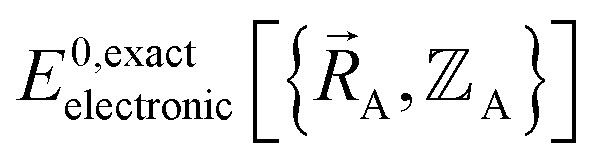
 and 
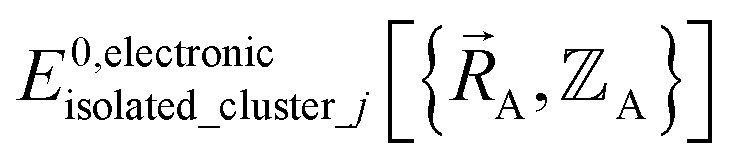
 as shown in eqn (59), this means 
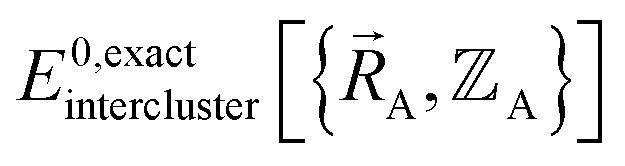
 may exhibit derivative discontinuities wherever either the full system or any of the associated isolated bonded clusters undergoes a change in electronic ground state. This means 
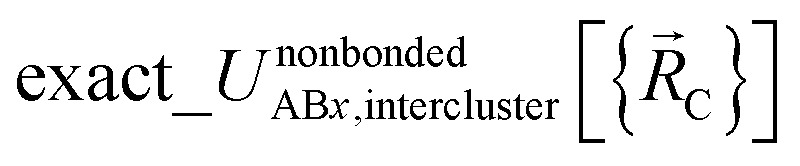
 has the following piecewise expansion86
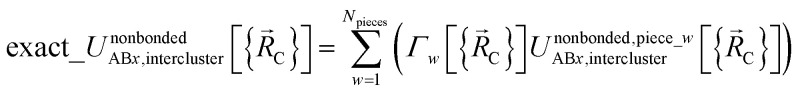
where87
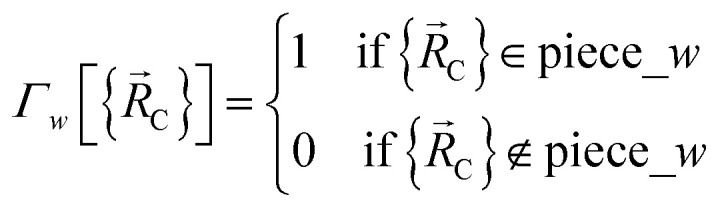


All locations inside piece_*w* share the same electronic ground-state type of the full system. All locations inside piece_*w* also share the same electronic ground-state type of isolated cluster_*j*. Locations inside two different pieces have either different electronic ground-state types for the full system or for any associated isolated bonded cluster. 
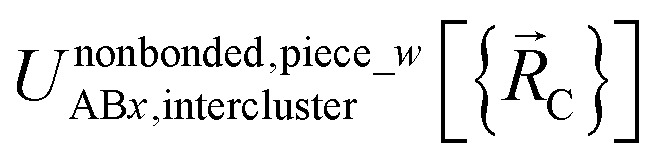
 is continuous and continuously differentiable (up to some order) with respect to atom displacements (*i.e.*, changes in 
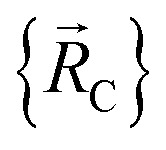
).

We normally only know values of 
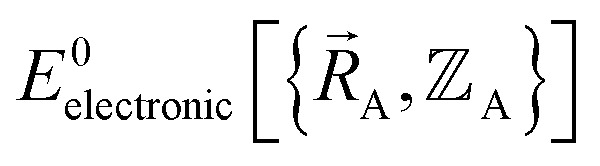
 that have been numerically computed for several chosen chemical geometries. Consequently, we have to use regression techniques to build a model for 
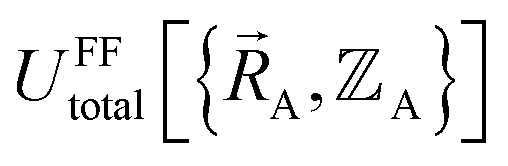
. In practice, approximate expressions are normally used to build the forcefield's 
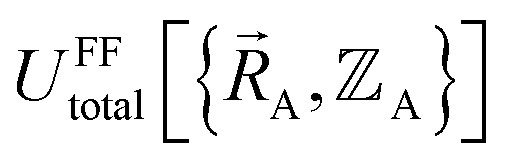
 functional, and this introduces a (hopefully small) difference between 
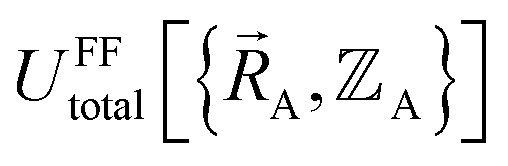
 and 
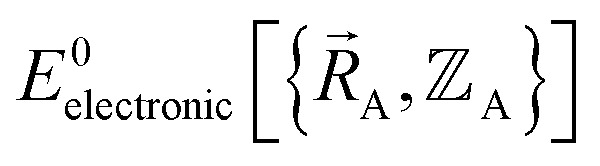
:88
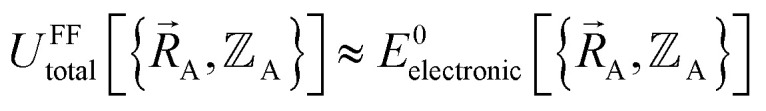


Although careful accounting of electronic ground-state subdomains is required to construct the exact forcefield, normally it is much easier to pursue an approximate treatment in which we focus on training the forcefield over particular region(s) of 
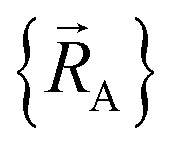
 space. 
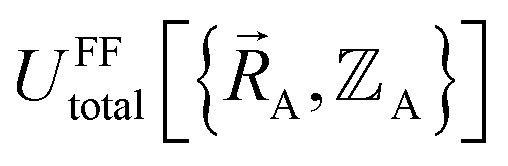
 only provides reasonable approximation to 
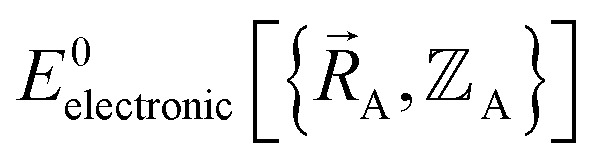
 within the general region(s) of 
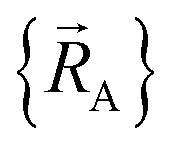
 space for which it was trained (*i.e.*, fitted, parameterized). As an approximation, 
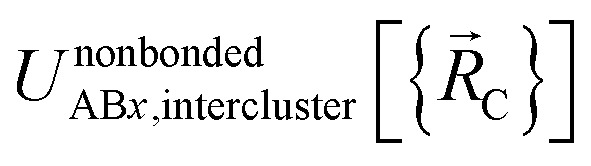
 is often oversimplified to have only a single piece (*i.e.*, *N*_pieces_ = 1) and 
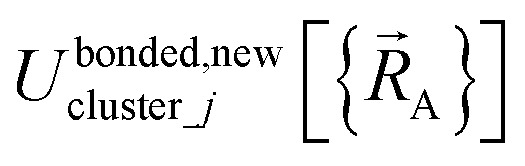
 is often oversimplified to have only one subdomain (*i.e.*, *N*^domains^_*j*_ = 1). These approximations make forcefield training easier.

### 2.6 Worked examples

#### 2.6.1 The stretched H_2_ molecule

Full configuration interaction (FCI) calculations were performed for the H_2_ molecule. Because the H_2_ molecule contains only two electrons, FCI calculation for this molecule corresponds to configuration interaction with single and double excitations (*i.e.*, CISD). CISD calculations were performed in Gaussian (ref. 95) software using the aug-cc-pVQZ^96–98^ basis set. These calculations solved the time-independent multi-electronic Schrodinger equation

89 *Ĥ*_el_*Ψ*^0^_electronic_ = *E*^0^_electronic_*Ψ*^0^_electronic_

using the following multi-electronic Hamiltonian operator (expressed in atomic units):90
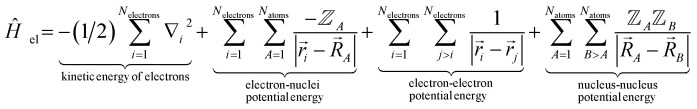


Eqn (89) and (90) are discussed in common quantum chemistry textbooks.^99–101^ In eqn (89), *Ψ*^0^_electronic_ is the ground-state multi-electronic wavefunction. The multi-electronic Hamiltonian operator in eqn (90) is one of several possible choices that can be used in this theoretical framework. If desired, the multi-electronic Hamiltonian operator could include various relativistic corrections, spin–orbit coupling, and/or spin–spin magnetic coupling, *etc.*^99–101^ For simplicity, those interactions were not included in the example studied in this section.

In the absence of externally applied fields, the only independent internal geometric coordinate for this molecule is its bond length. The FCI/aug-cc-pVQZ optimized bond length for the H_2_ singlet spin state is 74.199 picometer (pm). The triplet energy was also computed at this same bond length. For both the singlet and triplet spin states, FCI/aug-cc-pVQZ calculations were also performed at a series of constrained bond lengths from 50 to 500 pm.

For each H atom, the aug-cc-pVQZ basis set contains six sets of s-type basis functions, four sets of p-type basis functions, three sets of d-type basis functions, and two sets of f-type basis functions.^97^ One s-type basis function is a contraction of multiple Gaussian exponents.^97^ The other five s-type basis functions and all of the p, d, and f basis functions contain one Gaussian exponent per basis function.^97^ As shown in Table S1 of ESI,† the FCI/aug-cc-pVQZ singlet and triplet energies at 500 pm are less than 10^−4^ hartree away from the complete basis set limit value of exactly minus one hartree for two isolated hydrogen atoms. This shows the FCI/aug-cc-pVQZ results for the H_2_ molecule are close to the complete basis set limit. Accordingly, the quantum-mechanically-computed results listed in Table S1 of ESI† are a nearly exact solution to eqn (89) and (90).

At each finite bond length, the exact (*i.e.*, FCI near the complete basis set limit) spin triplet electronic energy of H_2_ is higher than the spin singlet electronic energy of the same bond length. At infinite bond length, the exact (*i.e.*, FCI near the complete basis set limit) spin triplet and spin singlet electronic energies of H_2_ are equal. Accordingly, there is no singlet-to-triplet electronic ground-state crossover for this molecule at the FCI level of theory near the complete basis set limit. Thus, only one electronic ground-state subdomain (*i.e.*, the singlet electronic ground state) is needed to construct the nonreactive forcefield for this molecule.

For the H_2_ molecule, there are no intracluster nonbonded interactions, because each H atom is directly bonded to the other H atom. Because the forcefield is nonreactive, this bond remains active even as the bond length is stretched to arbitrarily large distances.

For this molecule, the bond stretch energy can be represented exactly by the following expansion:91
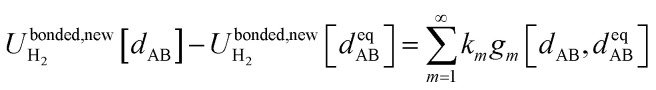
92
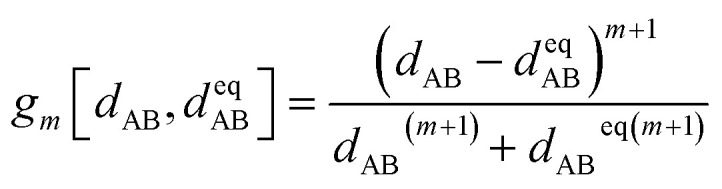
Note that *g*_*m*_ = 0 and ∂*g*_*m*_/∂*d*_AB_ = 0 at *d*_AB_ = *d*^eq^_AB_; *g*_*m*_ = −1 (if *m* is odd) or +1 (if *m* is even) at *d*_AB_ = 0, and *g*_*m*_ = 1 at *d*_AB_ = ∞. Eqn (92) is not the only possible choice of flexibility terms to expand the bond stretch energy, but it is a reasonable and workable choice. The *d*_AB_ = *d*^eq^_AB_ datapoint yields93
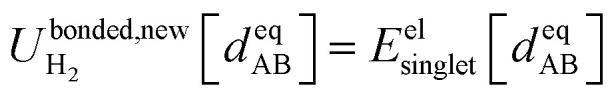


Truncating the summation in eqn (91) at *m* = 18 yields 18 force constants that can be computed by using linear regression to fit the bonded interaction model to a training dataset containing the 18 *d*_AB_ ≠ *d*^eq^_AB_ quantum-mechanically-computed *E*_singlet_ datapoints from Table S1 of ESI.† To handle the multicollinearity issue, I performed this linear regression using the LASSO method. This minimized the following loss function:94
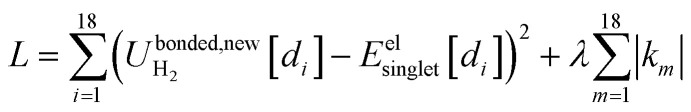
where *λ* is the LASSO regularization parameter.

The Matlab lasso function was used with the following settings: intercept = false, standardize = false, RelTol = 10^−8^, MaxIter = 10^9^. As lambda decreased, the number of nonzero parameters increased and the root-mean-squared-error (RMSE) of the training dataset decreased. Table S2 of ESI† lists the resulting force constant values for several values of *λ*. The optimized force constants had the following numbers of nonzero values: 7 for *λ* = 10^−8^, 9 for *λ* = 10^−9^, 12 for *λ* = 10^−10^, 17 for *λ* = 10^−11^, and 18 for *λ* ≤ 10^−12^. The sum of absolute values of the force constants increased as *λ* decreased.

Due to the multicollinearity issue, the solution for *λ* = 0 is ill-defined. Instead, one generally refers to a *λ* → 0 result that means the smallest value of *λ* for which converged results were computed. As the LASSO regularization parameter *λ* becomes closer to zero in value, the number of iterations allowed for convergence (*i.e.*, MaxIter) needs to be increased and the RelTol needs to be decreased. Here, the tightest convergence achieved was for RelTol = 10^−9^, MaxIter = 10^10^, and *λ* = 10^−20^ as shown in the last column of Table S1 of ESI.†

**Fig. 3:**
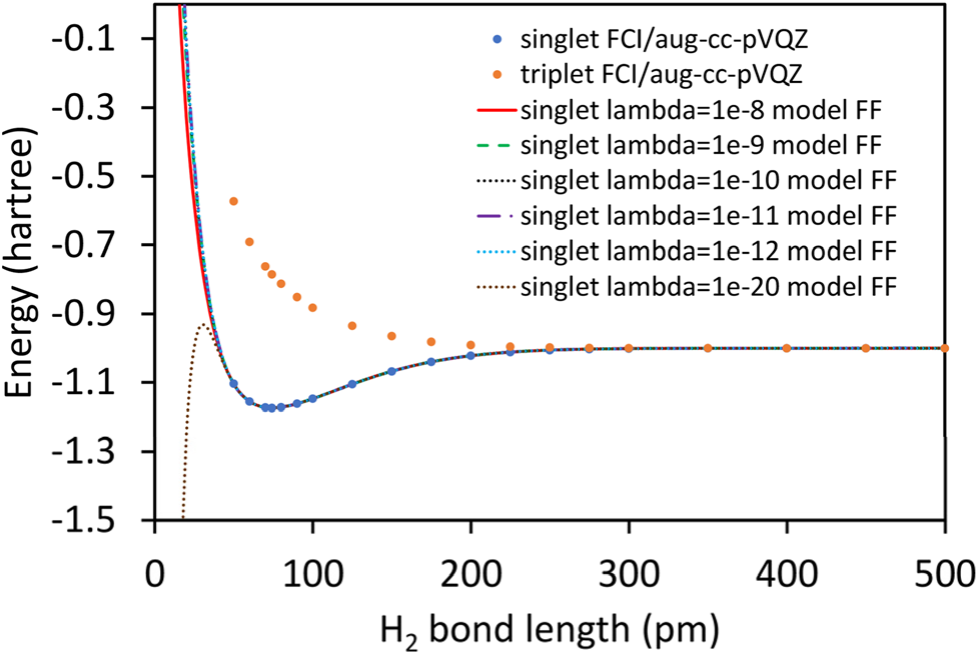
Born–Oppenheimer potential energy surface for the H_2_ molecule. Quantum chemistry results are compared to fitted forcefield models.

As shown in Table S1 of ESI,† this fitted forcefield nearly reproduced the quantum-mechanically-computed training data. The RMSE values (in hartree) for the training dataset decreased monotonically from 2.0 × 10^−4^ for *λ* = 10^−8^ to 3.4 × 10^−5^ for *λ* = 10^−20^. Fig. 3 plots the QM-computed singlet and triplet energies for the H_2_ molecule as a function of bond length. For the singlet state, the model forcefield curves are shown for comparison and are in extremely close agreement to the FCI/aug-cc-pVQZ spin singlet data. However, the *λ* = 10^−20^ model behaves erratically in the extrapolated region for bond lengths <40 pm. This clearly demonstrates that using *λ* values too close to zero causes the over-fitting problem that decreases the model's accuracy for describing regions outside the training data.

In summary, this example illustrates a practical implementation of parameterizing a formally exact nonreactive forcefield for an isolated bonded cluster. Formal exactness means that by improving the computational accuracy and precision, the parameterized model can be made infinitesimally close to the exact solution without having to leave the theoretical framework. Some ways to increase the computational accuracy and/or precision include:

(1) Increasing the basis set size is one aspect of ‘improving the computational accuracy and precision’. Although the aug-cc-pVQZ basis set already gets fairly close to the complete basis set limit for this molecule, increases in the basis set size would enable the quantum chemistry results to get even closer to the complete basis set limit. As the basis set size tends towards infinite, the complete basis set limit can be reached.

(2) The real number models used in the quantum chemistry calculations and the linear regression calculations have a finite storage size (*e.g.*, 64 bit real numbers) that determines the number of stored digits. Increasing the number of stored digits would enable these calculations to get even closer to the exact solution.

(3) Iterative quantum chemistry calculations (such as the FCI calculations performed here) employ convergence tolerances that allow the energy to be computed to some finite number of significant digits. Tightening these convergence tolerances would enable the quantum chemistry results to get even closer to the exact solution.

(4) The LASSO method for performing regularized linear regression uses a convergence tolerance, maximum number of allowed iterations, and a regularization parameter (*λ*). *λ* > 0 values increase the model's transferability, robustness, and conciseness at the expense of introducing some approximation. To solve the linear regression problem exactly, an infinitesimal *λ* → 0 solution is required together with extremely tight convergence tolerances, and this might require an extremely large number of iterations to converge.

(5) Here, the bonded interaction series expansion was truncated and a finite number of quantum-mechanically-computed datapoints were used to train the forcefield model. To achieve exactness for all geometries within the relevant connected region of the potential energy landscape, the training dataset would need to be expanded to include all such geometries (an infinite number) and an untruncated bonded interaction series expansion would need to be used. Additionally, the linear regression problem would need to be solved exactly so that the forcefield model exactly reproduced the training dataset.

My new ansatz for separating bonded interactions from nonbonded interactions works for developing forcefields using either machine-learning or non-machine-learning approaches. The example studied in this section used a series expansion containing many flexibility terms. Series expansion approaches can be useful to parameterize machine-learned forcefields that have been used to study many materials.^102–107^ A key advantage of machine-learning strategies is that they can be applied across a wide range of different systems without requiring as much manual human labor to develop an effective working model. Such machine-learning methods allow high accuracy to be reached at the expense of typically requiring a relatively large number of fitted parameters.^102–107^

However, it is often desirable to construct and use frugal forcefields that contain relatively small numbers of fitted parameters associated with carefully chosen physically-motivated forcefield terms. This is desirable, because atomistic simulations (*e.g.*, classical molecular dynamics and Monte Carlo simulations) using frugal forcefields can run quicker than atomistic simulations employing parameter-heavy forcefields containing a relatively large number of forcefield terms. The next section revisits this example using a bond stretch model potential that achieves high accuracy using a small number of fitted parameters.

#### 2.6.2 A new first-principles-derived bond stretch potential

The harmonic stretch model potential is simple to apply, but it only describes the shape of the bond stretch curve for small magnitude displacements (both bond compression and elongation) near the optimized bond length. As a bond is stretched to ever larger values, the energy of the harmonic bond stretch model potential increases proportional to the square of the displacement length, eventually becoming infinitely large in energy as the bond length is stretched to infinity.

For practical applications, it is often desirable to use a bond stretch model potential having a realistic shape. Here, I introduce a new first-principles-derived stretch model potential:95
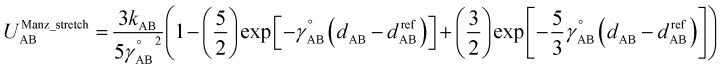


The well-known Morse^48^ potential96
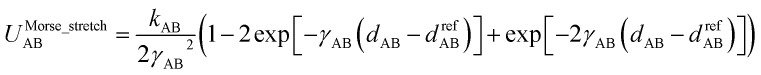
has a related form, but with different coefficients and exponents. Although the Morse potential was originally proposed based on empirical arguments, later authors provided some physically-based rationalizations for its form.^108,109^ As the bond length is stretched to infinity, these stretch potentials approach the predicted bond dissociation energy of97
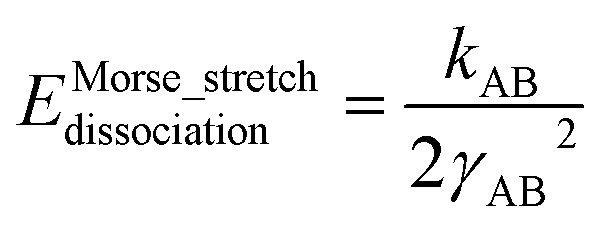
98
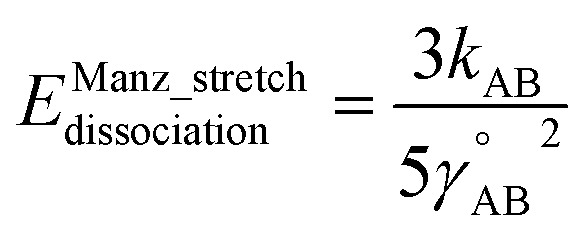


The Morse and Manz stretch potentials have the same number of parameters, but they have different numbers of empirically-fitted parameters. In the Morse stretch potential, the exponent *γ*_AB_ is an empirically-fitted regression parameter that normally requires nonlinear optimization. In the Manz stretch potential, 
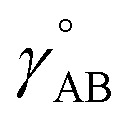
 is a quantum-mechanically-computed physical property not an empirically-fitted regression parameter. My new stretch potential provides a good tradeoff between accuracy and computational cost without requiring nonlinear regression when used with my new ansatz for separating bonded from nonbonded interactions. Analytic first-order through four-order derivatives of *U*^Manz_stretch^_AB_[*d*_AB_] are listed in ESI Section S3.†

Using my ansatz for separating bonded from nonbonded interactions, *d*^ref^_AB_ in eqn (95) or (96) always equals the (experimentally-measured or quantum-mechanically-computed) equilibrium bond length, *d*^eq^_AB_, in the isolated cluster's optimized geometry. Since my approach enables both 
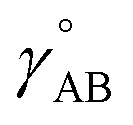
 and *d*^ref^_AB_ = *d*^eq^_AB_ to be computed directly, this facilitates using my stretch potential with forcefield parameterization protocols employing linear regression methods. In contrast, the old ansatz (for separating bonded from nonbonded interactions) requires the value of *d*^ref^_AB_ in eqn (95) or (96) to be an adjustable parameter *d*^resting^_AB_ that must be optimized using nonlinear regression techniques. However, iff the molecule is so small (*e.g.*, diatomic and triatomic molecules) that all intracluster nonbonded interactions are excluded, then in this limiting case *d*^resting^_AB_ = *d*^eq^_AB_ even under the old ansatz.

My stretch potential is derived *via* the following observations and steps:

(1) Consider a chemical system comprised of atomic nuclei and electrons with no externally applied fields (no external potentials). Within the Born–Oppenheimer approximation, this system's electronic energy is the sum of the electronic kinetic energy and the nuclear plus electronic potential energies:

99 *E*^0^_electronic_ = *T*_el_ + *V*_e–e_ + *V*_nucl–e_ + *V*_nucl–nucl_

When the AB bond length is at its optimized value,100
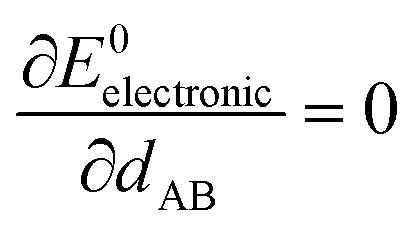


(2) The electron density of an isolated atom decays approximately exponentially in the atom's outer valence region (*i.e.* for large distances *r*_A_ from the atom's nucleus), and the value of this decay exponent *b*_A_ relates to the isolated atom's first ionization energy (I.E.) *via* I.E. ≈−*b*_A_^2^/8.^110–112^

(3) The two major paradigms for assigning atoms in materials are: (a) the overlapping atoms-in-materials paradigm and (b) the non-overlapping atom-in-materials paradigm. Quantum Chemical Topology (QCT), which contains Bader's quantum theory of atoms in molecules (QTAIM) as a subpart, is currently the main theoretical and computational framework amongst those methods within the non-overlapping atoms-in-materials paradigm.^113–120^ The Standard Atoms in Materials Framework (SAMF), which contains the Density-Derived Electrostatic and Chemical (DDEC) methods as a subpart, is currently the most accurate and versatile theoretical and computational framework within the overlapping atoms-in-materials paradigm.^121–123^ To date, the best performing electron-density partitioning methods within the overlapping atom-in-materials paradigm assign atom-in-material electron density distributions 
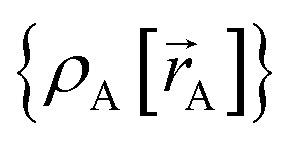
 such that their spherical averages {*ρ*^avg^_A_[*r*_A_]} decay approximately exponentially with increasing distance (*r*_A_) from the atom's nucleus.^9,122,124–127^ Among these approaches, the DDEC6 method is the current state of the art.^9,60,61,128^

(4) It is useful to construct a set of atomic orbitals in materials (AOIMs) that describe the effective electronic spin–orbitals of each atom in the material according to the following criteria. Criterion 1: each AOIM is the product of an orbital function and a spin ket, and the orbital function is an exact atomic orbital angular momentum eigenfunction. This means that each AOIM is a pure spherical harmonic function 
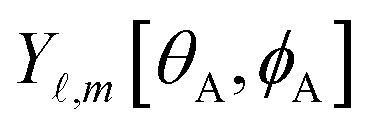
 times a radial function *ζ*^A^_*i*_[*r*_A_] times a spin ket |*s*^A^_*i*_〉.101
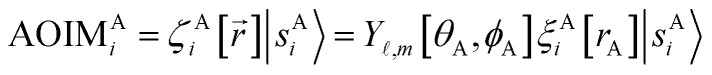
Criterion 2: AOIMs on the same atom are orthonormal to each other:102
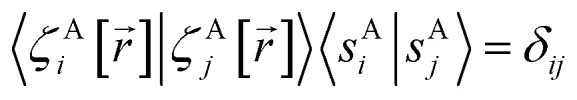
(AOIMs on two different atoms can have non-zero overlap) Criterion 3: these AOIMs have electron populations that satisfy the Pauli^129^ exclusion principle:

103 0 ≤ *n*^A,↑^_*i*_, *n*^*A*,↓^_*i*_ ≤ 1

where *n*^A,↑^_*i*_ and *n*^A,↓^_*i*_ are the number of spin-up and spin-down electrons, respectively, on atom A occupying 
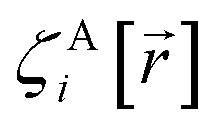
. Criterion 4: the occupation-weighted electron densities of AOIMs on atom A sum to the assigned atom-in-material electron density of atom A:104
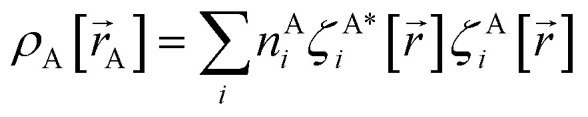
where

105 *n*^A^_*i*_ = *n*^A,↑^_*i*_+*n*^A,↓^_*i*_

and * denotes complex conjugation. This requires that we include enough AOIMs so that all of the electron density will get projected onto the combined set of AOIMs. Criterion 5: the AOIMs are constructed according to some scheme that gives them good transferability for small changes in the material's geometry. This means that the shapes and occupations of individual AOIMs do not change drastically when a bond is slightly stretched or compressed. Of course, the center of each AOIM will move along with the position 
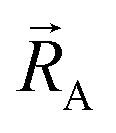
 of the atom to which it belongs. (Criterion # 5 can be satisfied by optimizing each AOIM to be an approximate energy eigenfunction. Together, these five criteria make each AOIM resemble atomic 1s, 2s, 2p, 3s, 3p, 3d, *etc.* orbitals. I recently developed such a method and programmed it into the Chargemol code. The details will be published in future work.)

(5) In the limit 
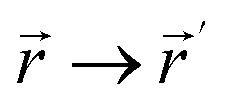
, the products 
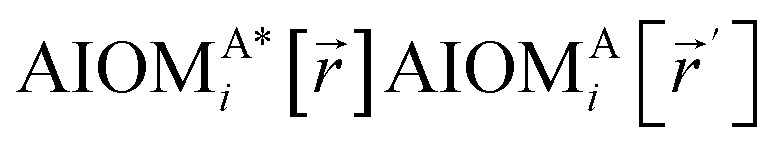
 and 
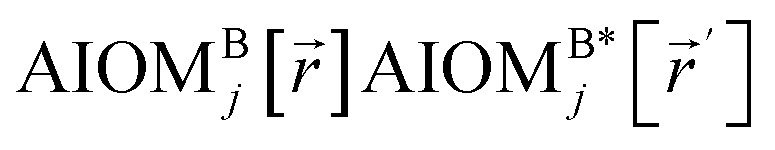
 approach the electron density in these orbitals. Because each AOIM approximately equals a polynomial function of radius times an exponential decay function, its spherically averaged electron density scales like:106
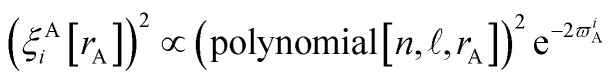

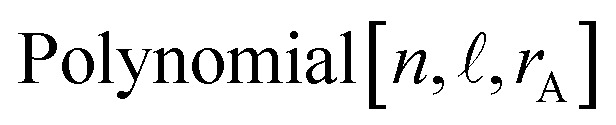
 causes each AOIM to oscillate such that it has 
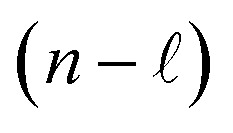
 radial nodes, where *n* is the AOIM's principle quantum number and 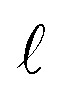 is its orbital angular momentum quantum number. For example, the 4p AOIM has (4 − 1) = 3 radial nodes. For valence AOIMs, these nodes occur in the core and semi-core regions. Well beyond the last radial node for large *r*_A_ (*i.e.*, in the valence region between atoms A and B), we can absorb the polynomial dependence into an effective exponent that approximately equals the effective decay exponent of the atom's valence density:107
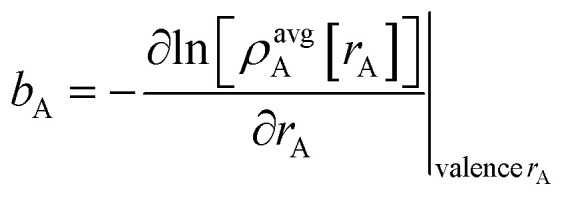

108 (*ξ*^A^_*i*_[*r*_A_])^2^|_valence *r*_A__ ∝ e^−*b*_A_*r*_A_^

(6) The energy of the AB bond is affected by exchange, kinetic energy, coulombic, and dispersion interactions between AOIMs on atom A and AOIMs on atom B. Depending on the circumstances and the value of *d*_AB_, the sum of energy contributions to bonding could be net attractive or net repulsive. For simplicity, we can classify these energy contributions into two major groups: (i) Group # 1 comprises interatomic kinetic and potential energy changes proportional to 
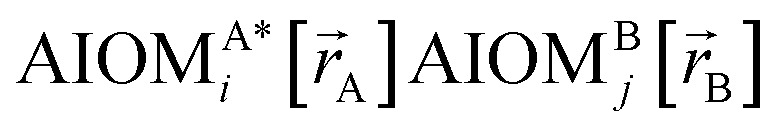
. By the triangle distance inequality,

109 *r*_A_ + *r*_B_ ≥ *d*_AB_

Due to the exponential decay of these AOIMs' radial functions, the largest contributions to 
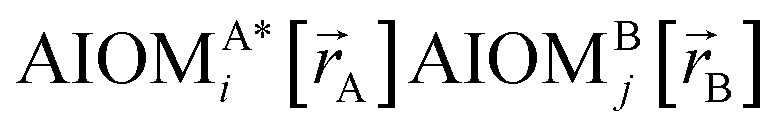
 occur for points satisfying

110 *r*_A_ + *r*_B_ ≈ *d*_AB_

which correspond to points near the bond's axis. This allows us to approximate this type of term as111
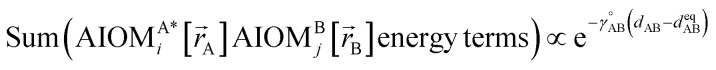


(ii) Group # 2 comprises the short-range repulsion (SRR) energy due to Pauli's^129^ exclusion principle. When two atoms overlap, their electron orbitals must deform (change) to retain orthogonality between all of the molecular orbitals. This raises the energy of the electrons, thus leading to a repulsive force between the overlapping electron clouds. For *d*_AB_ ≪ *d*^eq^_AB_, the SRR term dominates the energy function. This SRR energy contains both kinetic energy and potential energy contributions and has an exponential dependence on *d*_AB_ with a decay exponent approximately equal to 0.83 (*i.e.*, (5/6)) times some weighted average between *b*_A_ and *b*_B_:^8^112
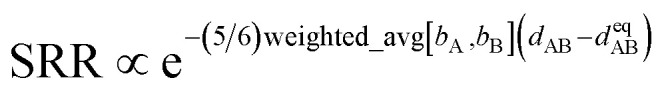


(7) A precise value for the exponent 
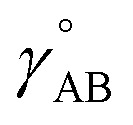
 can be derived by noting the scaling behavior of the overlap integral between two Slater functions113
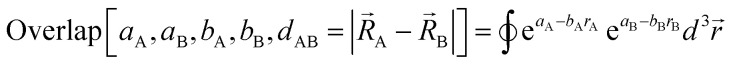
When *b*_A_ ≈ *b*_B_, this integral has the value^8^114
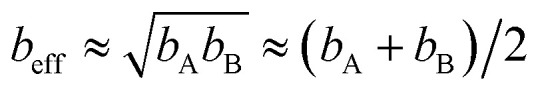
115
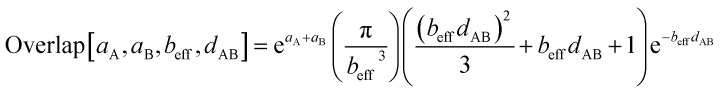


On the other hand, when 0 < *b*_A_ ≪ *b*_B_, then atom B looks almost like a point charge distribution so that the overlap becomes

116 Overlap[*a*_A_, *a*_B_, *b*_A_ ≪ *b*_B_, *d*_AB_] ≈ e^*a*_A_−*b*_A_*d*_AB_^*N*_B_

where117
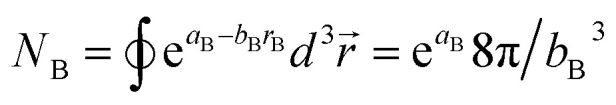
is the volume integral of the Slater function on atom B. For the overlap between the products of valence AOIMs, 
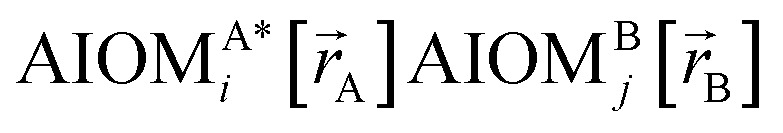
, the effective decay exponents of these valence AOIMs is approximately *b*_A_/2 and *b*_B_/2, respectively. Choosing118
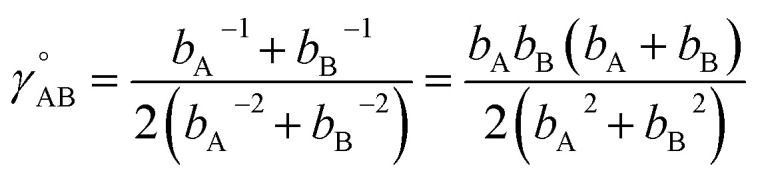
yields the appropriate limits:119
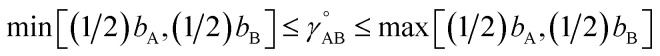
120
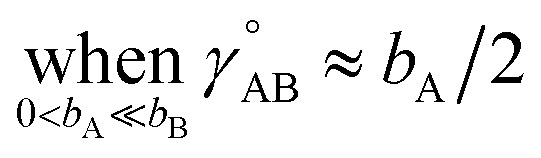
121
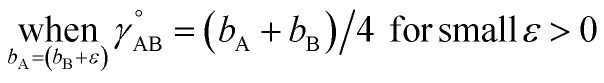


(8) Expressed as a Taylor series:122
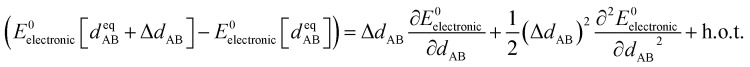


Because of eqn (100), the first non-zero term in eqn (122) is proportional to (Δ*d*_AB_)^2^ instead of Δ*d*_AB_. Assembling the above results means the energy equation should take the form123
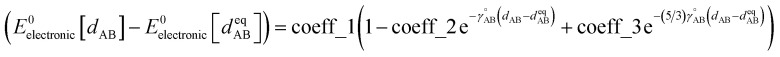


Since the right-hand side must equal zero when *d*_AB_ = *d*^eq^_AB_, this means

124 1 − coeff_2 + coeff_3 = 0

Since the force (and first derivative of energy) must be zero when *d*_AB_ = *d*^eq^_AB_, this means

125 coeff_2 − (5/3)coeff_3 = 0

Solving these two linear equations gives coeff_2 = 5/2 and coeff_3 = 3/2. Defining the force constant by126
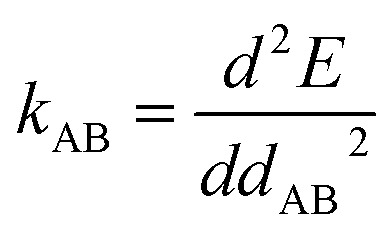
gives127
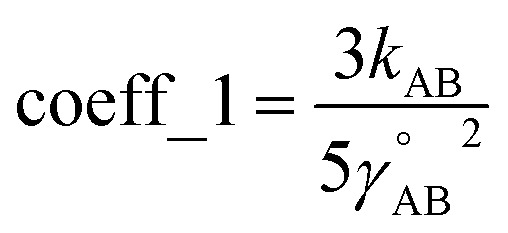
With these values for the coefficients, eqn (123) becomes my new stretch potential (eqn (95)).

My stretch potential is conceptually related to bond order changes. Bond orders computed using my bond order equation applied with DDEC6 partitioning (aka Manz/DDEC6 bond orders) decay approximately exponentially as the bond length is stretched beyond its equilibrium value.^128^ My bond order equals the number of electrons that are dressed exchanged between two atoms in a material.^128^ This bond order is also between approximately 1× and 2× the contact exchange (and DDEC6 overlap population) which also decay approximately exponentially as the bond length is stretched beyond its equilibrium value.^128^ For bonds having similar type, the integrated crystal orbital Hamilton population (ICOHP) strongly correlated to the computed Manz/DDEC6 bond orders in various materials.^130^ Moreover, Pauling proposed an empirical bond-distance-to-bond-order correlation in which the bond order decreases exponentially as the bond length increases.^131^

I now introduce a straightforward algorithm to compute *b*_A_ and *b*_B_ for the AB bond. First, we perform a quantum chemistry calculation on the material's optimized geometry. Then, we perform DDEC analysis on the quantum chemistry results. Starting with the {*ρ*^avg^_A_[*r*_*A*_]} printed by the Chargemol code, we first make sure these are monotonically decreasing functions by imposing

128 *

<svg xmlns="http://www.w3.org/2000/svg" version="1.0" width="12.769231pt" height="16.000000pt" viewBox="0 0 12.769231 16.000000" preserveAspectRatio="xMidYMid meet"><metadata>
Created by potrace 1.16, written by Peter Selinger 2001-2019
</metadata><g transform="translate(1.000000,15.000000) scale(0.013462,-0.013462)" fill="currentColor" stroke="none"><path d="M320 1000 l0 -40 -40 0 -40 0 0 -40 0 -40 40 0 40 0 0 40 0 40 80 0 80 0 0 -40 0 -40 80 0 80 0 0 40 0 40 40 0 40 0 0 40 0 40 -40 0 -40 0 0 -40 0 -40 -80 0 -80 0 0 40 0 40 -80 0 -80 0 0 -40z M400 760 l0 -40 -40 0 -40 0 0 -40 0 -40 -40 0 -40 0 0 -120 0 -120 -40 0 -40 0 0 -160 0 -160 -40 0 -40 0 0 -40 0 -40 40 0 40 0 0 40 0 40 40 0 40 0 0 120 0 120 40 0 40 0 0 -40 0 -40 120 0 120 0 0 40 0 40 40 0 40 0 0 40 0 40 40 0 40 0 0 160 0 160 -40 0 -40 0 0 40 0 40 -120 0 -120 0 0 -40z m240 -200 l0 -160 -40 0 -40 0 0 -40 0 -40 -120 0 -120 0 0 160 0 160 40 0 40 0 0 40 0 40 120 0 120 0 0 -160z"/></g></svg>

*^avg^_A_[*r*^nshells^_A_] = *ρ*^avg^_A_[*r*^nshells^_A_]

129 **^avg^_A_[*r*_A_^*j*^] = max[ρ^avg^_A_[*r*_A_^*j*^], *ρ̃*^avg^_A_[*r*_A_^*j*+1^]] for *j* = (nshells − 1), (nshells − 2),…1

starting with the outer radial shell and proceeding inward to successively smaller radial shells. This procedure is done for all atoms in the material's unit cell. We next identify which atoms in the material are directly bonded to each other (*i.e.*, are nearest neighbors in the bond connectivity graph) using a chosen method. For example, we could consider two atoms to be directly bonded to each other iff the distance between them was no greater than the sum of their element-dependent atom-typing radii:^10^

130 *d*_AB_ ≤ *R*^AT^_A_ + *R*^AT^_B_

Alternatively, one could consider two atoms A and B to be directly bonded to each other if their bond order BO_AB_, overlap population OP_AB_, or contact exchange CE_AB_ is above a chosen threshold value. For a bond pair AB, find (a) the smallest value of *r*_A_ for which *ρ̃*^avg^_B_[*d*_AB_ − *r*_A_] > *ρ̃*^avg^_A_[*r*_A_] and (b) the largest value of *r*_A_ for which *ρ̃*^avg^_B_[*d*_AB_ − *r*_A_] < *ρ̃*^avg^_A_[*r*_A_]; let *D*^AB^_A_ equal the average of these two *r*_A_ values. Next, we perform linear regression to fit the model *a*^AB^_A_ − *b*^AB^_A_*r*_A_ to the datapoints ln[*ρ̃*^avg^_A_[*r*_A_^*j*^]] over the set of radial shells satisfying

131 ((*D*^AB^_A_ − 0.5 bohr)) ≤ *r*_A_^*j*^ ≤ (*D*^AB^_A_ + 2.5 bohr)

The sampled range of [*r*^*j*^_A_] values is asymmetric about *D*^AB^_A_ to emphasize the outer valence region of atom A. This ensures the computed exponent *b*^AB^_A_ is fairly characteristic of the *ρ̃*^avg^_A_[*r*^*j*^_A_] decay behavior over the most relevant range of *r*_A_ values. This process is repeated for atom B in the AB pair to get *b*^AB^_B_. These are plugged into eqn (118) to compute 
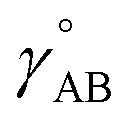
. Finally, this process is repeated for all bond pairs in the material.

This process assigns a different *D*^AB^_A_ and hence different *b*^AB^_A_ value for each different bond connected to atom A. For example, in the acetonitrile molecule (H_3_C–C≡N), the central C atom is singly bonded to another carbon atom and triply bonded to a nitrogen atom. This has the effect of making the central carbon atom's *D*^AB^_A_ slightly smaller for the triple bond than for the single bond, which means the resulting *b*^AB^_A_ is fitted over slightly smaller *r*_A_ values for the triple bond compared to the single bond. As another example, we could consider a hydrogen atom that is covalently bonded to one oxygen atom and opportunistically ‘hydrogen bonded’ to another oxygen atom. Since the opportunistic ‘hydrogen bond’ has a greater length than the O–H covalent single bond, the procedure described above automatically fits *b*^AB^_A_ for each bond over the relevant *r*_A_ values for that particular bond. Accordingly, this procedure should provide good results for a wide range of bond orders.

**Fig. 4:**
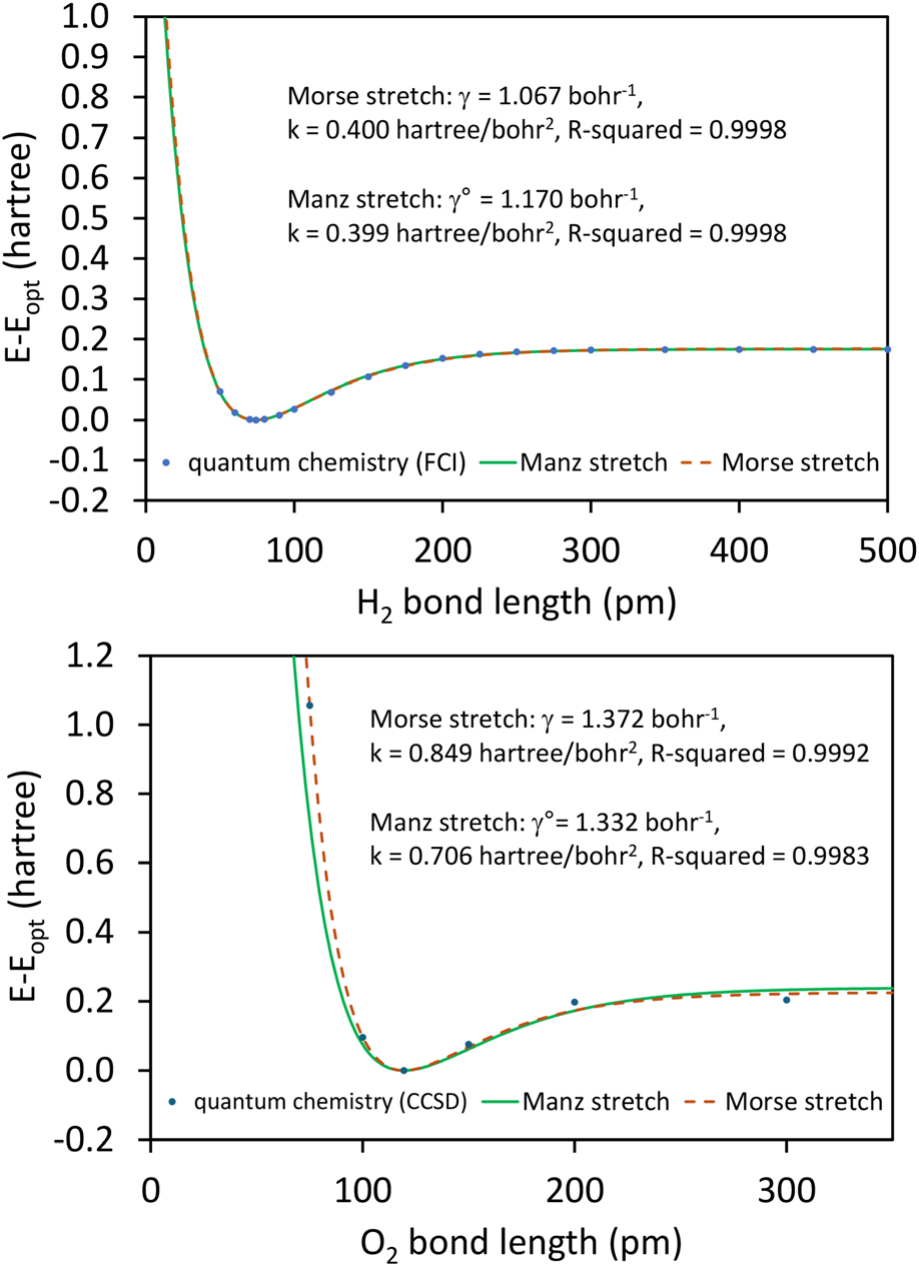
Comparison of Morse and Manz stretch potentials fitted to the quantum-mechanically-computed H_2_ singlet (top panel) and O_2_ triplet (bottom panel) potential energy curves.

This procedure was used to analyze the stretched H_2_ singlet molecule using the FCI/aug-cc-pVQZ quantum chemistry calculations introduced in the previous section. Also shown are CCSD calculations for the spin triplet O_2_ molecule using the d-aug-cc-pVQZ^96–98^ basis set (These were performed using Gaussian (ref. 95) software.) As shown in Fig. 4, both the Morse and Manz stretch potentials fit the quantum-mechanically-computed data well. These regression parameters were optimized in Excel using the Generalized Reduced Gradient (GRG) solver that works for both linear and nonlinear optimization problems. The Manz stretch potential has the advantage of requiring only a linear regression, while the Morse potential required a nonlinear regression to optimize its parameters.

The ‘goodness of fit’ (*R*-squared) was computed as follows:

132 *R*^2^ = 1 − SSE/SST

where SSE = sum of squared errors and SST = sum of squares total. In this case,133
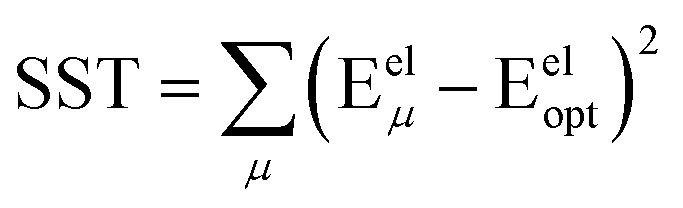
134
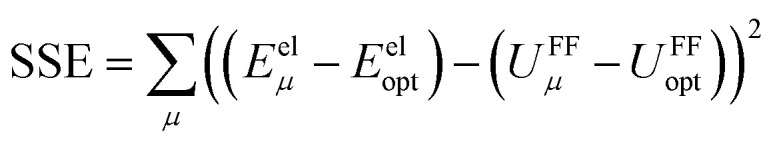
where the summation runs over the geometries in the dataset.

By fitting the empirical Morse stretch potential to the first-principles-derived Manz stretch potential, a hack was developed to accurately estimate the Morse potential exponent *γ*^Morse^_AB_. Equating the Morse potential's force constant (*k*) and dissociation energy (*E*^Morse_stretch^_dissociation_, eqn (97)) to those of the Manz stretch potential (eqn (98)) yields:135
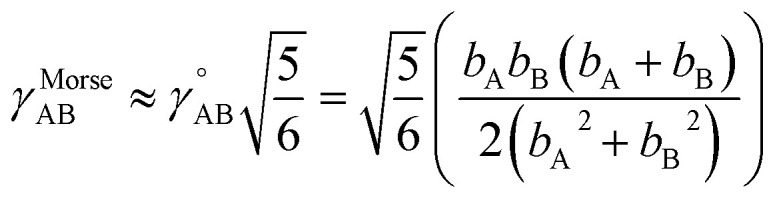


For the H_2_ molecule, this equation predicts *γ*^Morse^_AB_ ≈ 1.068 bohr^−1^ compared to the optimized value of 1.067 bohr^−1^. For the O_2_ molecule, this equation predicts *γ*^Morse^_AB_ ≈ 1.216 bohr^−1^ compared to the optimized value of 1.372 bohr^−1^. If using these predicted exponents in the Morse potential, the force constant *k*_Morse_ would be optimized (using linear regression) to yield 0.401 (H_2_) and 0.952 (O_2_) hartree per bohr^2^ with *R*-squared values of 0.9998 (H_2_) and 0.9881 (O_2_).

The above procedure does not require a combining (aka ‘mixing’) rule that relates *γ*^Morse^_AB_ and *γ*^Manz^_AB_ to *γ*^Morse^_AA_, *γ*^Morse^_BB_, *γ*^Manz^_AA_, and *γ*^Manz^_BB_. In the above procedure, 
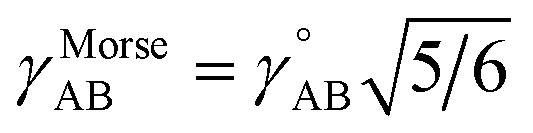
 and 
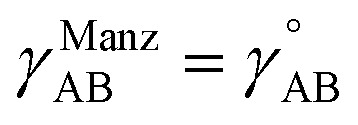
 are extracted directly from quantum chemistry calculations. From eqn (118) and (135), the following combining rules emerge as approximations:136
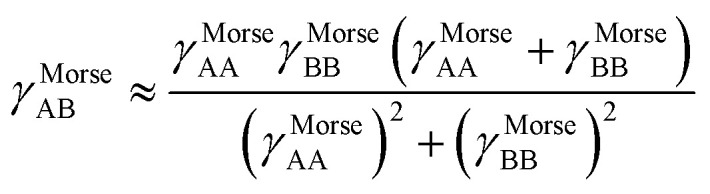
137
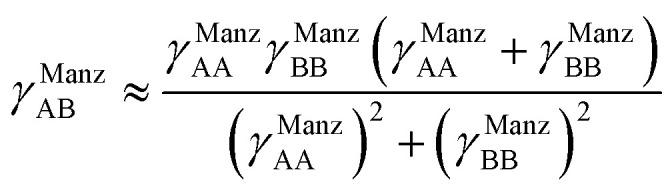


#### 2.6.3 A bond stretch in the hexafluorobenzene molecule

We now consider the C–F bond stretch force constant in the C_6_F_6_ molecule. This molecule was chosen for two reasons:

(1) First, its symmetry means there is only one independent atom-in-material charge value. Specifically, if *q*_C_ is the net atomic charge assigned to each carbon atom, then *q*_F_ = −*q*_C_ is the net atomic charge assigned to each fluorine atom.

(2) Second, this molecule includes first-, second-, third-, fourth-, and fifth-nearest neighbors. This ensures that some nonbonded interactions will still be present even when 1–2 (*i.e.*, first-neighbor), 1–3 (*i.e.*, second-neighbor), and/or 1–4 (*i.e.*, third-neighbor) nonbonded interactions are excluded in the forcefield model.

First, the geometry was fully optimized in Gaussian 16 (ref. 95) using the B3LYP^82,132,133^ exchange–correlation functional and def2-TZVPD^134^ basis set. The geometry was optimized to tight convergence criteria (*i.e.*, Gaussian keyword opt = tight). In the fully optimized geometry, the optimized bond lengths were 1.332 (C–F) and 1.389 Å (C–C), the optimized bond angles were 120.0° (both C–C–C and C–C–F), and all atoms were in the same plane.

**Fig. 5:**
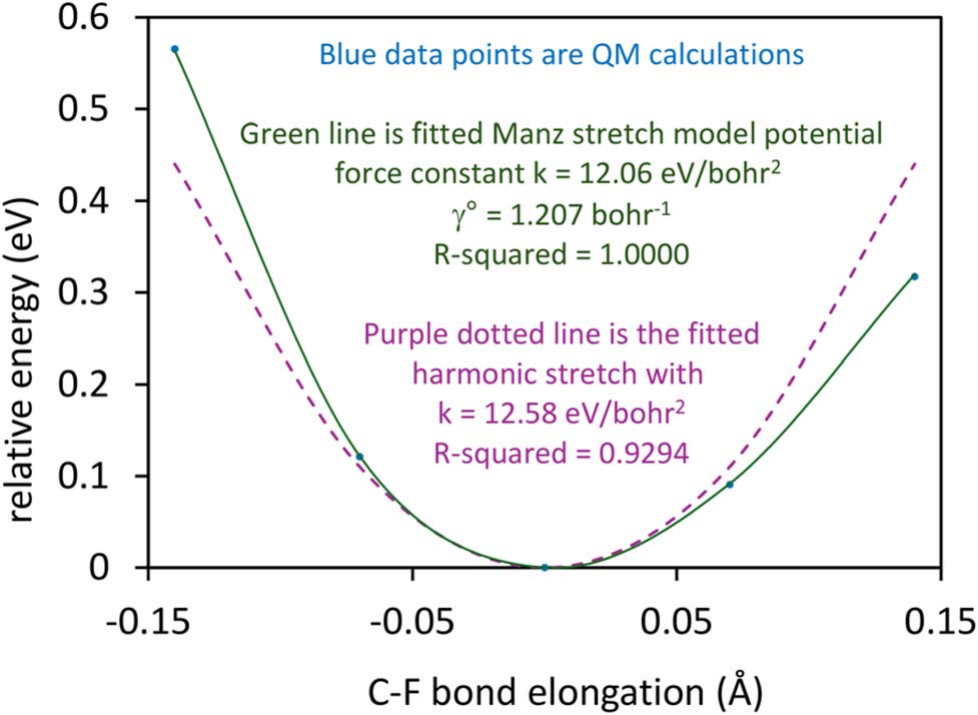
Quantum-mechanically-computed Born–Oppenheimer potential energy curve for the C–F bond stretch in the C_6_F_6_ molecule. Fitted harmonic and Manz stretch model potentials are shown for comparison.

Next, single-point energies were computed when the length of one C–F bond was changed by −0.14, −0.07, +0.07, and +0.14 Å compared to its length in the fully optimized geometry. This was done by moving one F atom in the C_6_F_6_ plane while holding the positions of all other atoms rigid at the same positions they had in the fully optimized geometry. All bond angle values were the same as in the fully optimized geometry. Since all atoms remained in the plane, no changes in dihedral values occurred. In summary, only the value of one internal coordinate (*i.e.*, the length of one and only one C–F bond) changed. This allows us to isolate the energy change of a single flexibility term; namely, the bond stretch for this one bond as shown in Fig. 5.

I wrote and used the calculate_Manz_and_Morse_stretch_potential_exponents program to compute the exponent *γ*°. This program uses the computational algorithm described in Section 2.6.2 above to analyze the DDEC6-computed {*ρ*^avg^_A_[*r*_A_]} printed from the Chargemol^60^ program. The calculate_Manz_and_Morse_stretch_potential_exponents program can handle any number (*i.e.*, 0, 1, 2, or 3) of periodic boundary conditions and works for molecules, dense and porous solids, solid surfaces, polymers, nanotubes, nanosheets, ionic and covalent materials, opportunistically-hydrogen-bonded materials, magnetic and non-magnetic materials, *etc.* For the C–F bond in C_6_F_6_, the result was *γ*° = 1.207 bohr^−1^ using the fully optimized geometry.

The stretch force constants were optimized by minimizing the following least-squares loss function:138
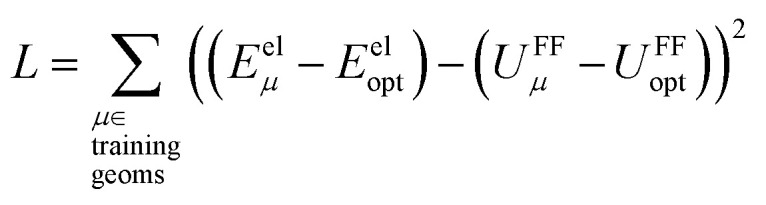
In eqn (138), *μ* is the geometry number in the training dataset (In this example, there are a total of four nonequilibrium geometries in the training dataset.). *E*^el^_*μ*_ is the quantum-mechanically-computed energy of geometry *μ*. *E*^el^_opt_ is the quantum-mechanically-computed energy of the fully optimized ground-state geometry. *U*^FF^_*μ*_ is the forcefield model's potential energy of geometry *μ*. *U*^FF^_opt_ is the forcefield model's potential energy of the fully optimized ground-state geometry. These optimizations were performed using Excel's GRG solver.

The forcefield's potential energy was expanded as the sum of bonded interactions plus non-bonded interactions:

139 *U*^FF^_*μ*_ = *U*^bonded^_*μ*_ + *U*^nonbonded^_*μ*_

The atomic charges plus Lennard-Jones parameters model *U*_AB_^(*q*+LJ)^ (eqn (9)) was used for these nonbonded interactions. Comparisons were made using different values for the atomic charges and Lennard-Jones parameters. No cutoff distance for the nonbonded interactions (*d*^nonbonded^_cutoff_) was used for these calculations. Models were built and compared using the harmonic stretch (eqn (10)) and Manz stretch (eqn (95) and (118)) potential for the bonded interaction.

Nonbonded interactions were always excluded between bonded first-neighbors (aka 1–2 interactions) and bonded second neighbors (aka 1–3 interactions). Comparisons were made between including or excluding nonbonded interactions for bonded third neighbors (aka 1–4 interactions). More remote nonbonded interactions (*e.g.*, 1–5, 1–6, *etc.*) were always included.

Comparisons were made using either the old ansatz or my new ansatz for separating bonded from non-bonded interactions. When using the old ansatz, the reference bond length, *d*^ref^_AB_, becomes a regression parameter, *d*^resting^_AB_, which leads to a nonlinear optimization problem. When using my new ansatz, the reference bond length, *d*^ref^_AB_, becomes the optimized bond length, *d*^eq^_AB_, which gives a linear optimization problem for both the harmonic and Manz stretch potentials. For the new scheme, *U*^nonbonded^_AB*x*,intracluster_ = *U*_AB_^(*q*+LJ)^ was inserted into eqn (48) to compute *Φ*^intracluster^_AB*x*_ which was inserted into eqn (26) to compute *U*^nonbonded,new^ for each geometry. For the new scheme, the loss function of eqn (138) was minimized by varying the value of *k*^new^_AB_. For the old scheme, *U*^nonbonded^_AB_ = *U*_AB_^(*q*+LJ)^ was inserted into eqn (8) to compute *U*^old^_nonbonded_ for each geometry. For the old scheme, the loss function of eqn (138) was minimized by varying the values of *k*^old^_AB_ and *d*^resting^_AB_.

When using my new ansatz for separating bonded from nonbonded interactions, comparisons were made between the full potential model that included both intracluster bonded and intracluster nonbonded interactions (eqn (138) and (139)) and the leading-order potential model that included only intracluster bonded interactions. As explained in the previous sections, the leading-order potential model accurately describes the intracluster interactions up to and including second-order derivatives of the potential energy at the isolated cluster's optimized geometry.

The leading-order potential models for the harmonic and Manz stretch potentials are plotted in Fig. 5 and equal the full model potential results when all intracluster nonbonded interactions (*e.g.*, atomic charges and Lennard-Jones potential) are set to zero. *R*-Squared values were computed using eqn (132)–(134). As demonstrated by the results shown in Fig. 5, my stretch potential (*R*-squared = 1.0000) fit the QM data nearly perfectly while the harmonic stretch potential (*R*-squared = 0.9294) did not capture the bond's significant anharmonicity. For comparison, optimized values for the Morse stretch potential were: (a) *γ* (optimized) = 1.093 bohr^−1^, *k* (optimized) = 12.01 eV bohr^−2^, giving *R*-squared = 1.0000, and (b) *γ* (predicted using eqn (135)) = 1.102 bohr^−1^, *k* (optimized) = 12.00 eV bohr^−2^, giving *R*-squared = 1.0000.

The full potential model results are summarized in Table 2 (harmonic stretch) and Table 3 (Manz stretch). Results are compared for the new and old schemes using various parameters for the nonbonded interactions. *R*-Squared values were computed using eqn (132)–(134). Tests were performed for three charge values (*q*_C_ = 0, 0.10 (DDEC6), and 0.62 (QTAIM)) both with and without Lennard-Jones (LJ) interactions. The LJ interaction parameters were taken from the Universal Force Field (UFF): *d*^LJ^_C–C_ = 3.851 Å, *d*^LJ^_F–F_ = 3.364 Å, 
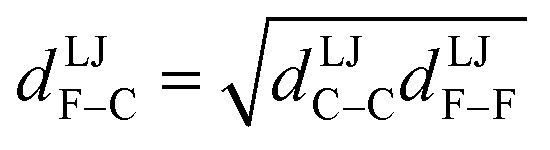
, *ε*^LJ^_C–C_ = 0.105 kcal mol^−1^, *ε*^LJ^_F–F_ = 0.050 kcal mol^−1^, 
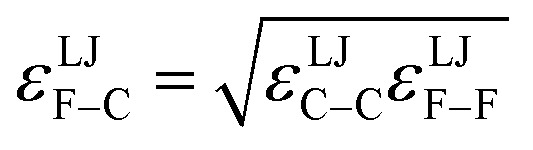
.^135^ Please see ref. 136–138 for a further discussion of forcefield nonbonded parameters for this molecule.

**Table 2:** Sensitivity of the new and old schemes to the nonbonded parameter model. One C–F bond in the C_6_F_6_ molecule was modeled using the harmonic stretch potential. The equilibrium C–F bond length is 1.332 Å

Atom charges	LJ parameters	1–4 nonbonded interactions included?	New scheme		Old scheme		
*q*_C_ (method)			*k* ^new^_AB_ (eV bohr^−2^)	*R*-Squared	*k* ^old^_AB_ (eV bohr^−2^)	*d* ^resting^_AB_ (Å)	*R*-Squared
0 (none)	0	Y, N	12.58	0.9294	12.58	1.349	0.9920
0.10 (DDEC6)	0	Y	12.58	0.9293	12.62	1.347	0.9919
0.10 (DDEC6)	0	N	12.58	0.9294	12.58	1.347	0.9919
0.62 (QTAIM)	0	Y	12.59	0.9285	12.80	1.340	0.9918
0.62 (QTAIM)	0	N	12.58	0.9295	12.56	1.338	0.9915
0 (none)	UFF	Y	12.58	0.9294	12.61	1.350	0.9921
0 (none)	UFF	N	12.58	0.9294	12.58	1.349	0.9920
0.10 (DDEC6)	UFF	Y	12.58	0.9292	12.65	1.348	0.9920
0.10 (DDEC6)	UFF	N	12.58	0.9294	12.58	1.347	0.9919
0.62 (QTAIM)	UFF	Y	12.59	0.9285	12.83	1.341	0.9918
0.62 (QTAIM)	UFF	N	12.58	0.9295	12.56	1.338	0.9915

**Table 3:** Sensitivity of the new and old schemes to the nonbonded parameter model. One C–F bond in the C_6_F_6_ molecule was modeled using the Manz stretch potential. The equilibrium C–F bond length is 1.332 Å

Atom charges	LJ parameters	1–4 nonbonded interactions included?	New scheme		Old scheme		
*q*_C_ (method)			*k* ^new^_AB_ (eV bohr^−2^)	*R*-Squared	*k* ^old^_AB_ (eV bohr^−2^)	*d* ^resting^_AB_ (Å)	*R*-Squared
0 (none)	0	Y, N	12.06	1.0000	12.02	1.3323^a^	1.0000
0.10 (DDEC6)	0	Y	12.06	1.0000	12.17	1.331	1.0000
0.10 (DDEC6)	0	N	12.06	1.0000	12.15	1.331	1.0000
0.62 (QTAIM)	0	Y	12.07	1.0000	12.92	1.323	1.0000
0.62 (QTAIM)	0	N	12.06	1.0000	12.82	1.321	1.0000
0 (none)	UFF	Y	12.06	1.0000	11.99	1.333	1.0000
0 (none)	UFF	N	12.06	1.0000	12.03	1.332	1.0000
0.10 (DDEC6)	UFF	Y	12.06	1.0000	12.13	1.332	1.0000
0.10 (DDEC6)	UFF	N	12.06	1.0000	12.16	1.330	1.0000
0.62 (QTAIM)	UFF	Y	12.07	1.0000	12.88	1.324	1.0000
0.62 (QTAIM)	UFF	N	12.06	1.0000	12.82	1.321	1.0000

a An extra significant digit is shown here to explain why the optimized *k* value equals 12.02 instead of 12.06.

As shown in Tables 2 and 3, the new scheme gave optimized force constant values that were practically the same irrespective of the non-bonded interaction model. Under the new scheme, only third-order and higher-order derivatives (*i.e.*, anharmonicities) of the potential energy are affected by fluctuations in the intracluster nonbonded parameters. For these reasons, the bonded force constant values are less sensitive to the particular choice of intracluster nonbonded potential model under the new scheme compared to the old scheme. This is an extremely important consideration, because nonbonded parameters such as atomic-in-material (AIM) charges, AIM multipole moments, AIM polarizabilities, AIM dispersion coefficients, Lennard-Jones parameters, *etc.* carry some uncertainties in their values.

As shown in Tables 2 and 3, the old scheme yielded force constants (*k*^old^_AB_) and resting values (*d*^resting^_AB_) that were moderately but not severely sensitive to the choice of nonbonded interaction model. My new stretch potential was able to describe the C–F bond's potential energy curve nearly perfectly (*i.e.*, *R*-squared = 1.0000) using both the new and old schemes for all of the nonbonded interaction models tested.

The harmonic stretch potential yielded higher *R*-squared values when using the old scheme compared to when using the new scheme. This was due to the old scheme's additional regression parameter (*i.e.*, *d*^resting^_AB_) compared to the new scheme which uses the quantum-mechanically-computed *d*^eq^_AB_ value. However, the old scheme has the disadvantage of predicting the wrong value for *d*^eq^_AB_. By construction, the new scheme yields the correct value *d*^eq^_AB_ = 1.332 Å. For the no charges and no LJ parameters model, the old scheme yields the value *d*^eq^_AB_ = 1.349 Å, which is close but not exact.

In summary, the new scheme is preferable to the old scheme for the following three reasons. (1) The new scheme requires only linear regression to optimize the force constants, while the old scheme sometimes (*e.g.*, Manz and Morse stretch potentials) requires nonlinear regression to optimize the force constants and resting values. (2) The new scheme gives optimized force constant values that are almost insensitive to the choice of nonbonded interaction model. (3) The new scheme exactly reproduces the material's optimized reference geometry in which all atom-in-material forces are zero.

## 3. A better angle-bending model potential

### 3.1 Derivation and comparison to other popular angle-bending model potentials

An angle bending potential (
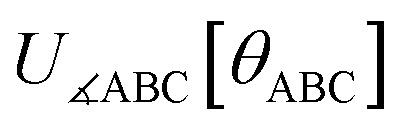
) models the potential energy change from a change of bond angle *θ*_ABC_ where atom A is bonded to atom B, and atom C is bonded to atom B. The angle *θ*_ABC_ is defined as:140
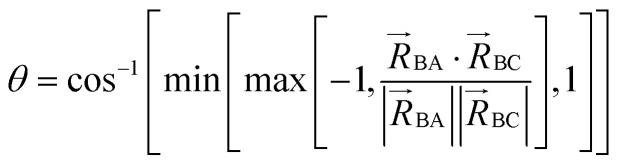
where 
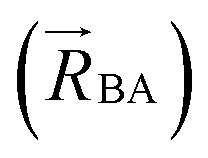
 is the vector from atom B to atom A defined as:141
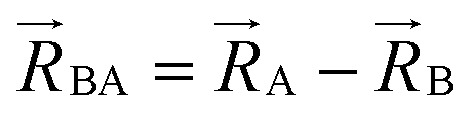
We used min and max functions to cancel the effect of roundoff error on the results; this forces the argument of the arccosine function to be between −1 and 1. *θ*_eq_ is the equilibrium value of this bond angle in the optimized ground-state geometry. The physically allowed ranges are

142 0 < *θ*_eq_ ≤ π

143 0 < *θ*_ABC_ ≤ π

*θ*_eq_ = 0 is not physically allowed, because this would correspond to either (i) two different atoms occupying the same nuclear position which is not allowed or (ii) all three atoms being collinear (*i.e.*, in a line) and in this case *θ*_eq_ would be interpreted as π instead of 0. Pauli repulsion^129^ (aka ‘short-range repulsion’^8^) prevents *θ*_ABC_ from getting close to zero.

As described in prior literature, the forces exhibited by the angle-bending potential on atoms A, B, and C are related to this potential's derivative 
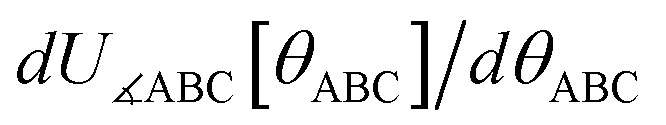
.^1^ At the linear angle value *θ*_ABC_ = π, the angle-bending force should be zero by symmetry, because the angle decreases as either atom A or atom C moves in any direction perpendicular to the starting line ABC.^5^ Accordingly, a physically viable angle-bending potential satisfies the constraint144
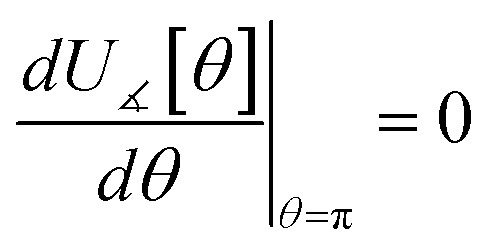


For derivatives of all orders to be continuous at *θ*_ABC_ = π, the angle-bending potential must be symmetric about *θ*_ABC_ = π, which requires:145
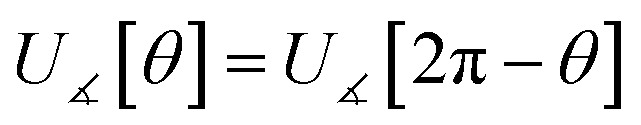
This requirement arises, because a hypothetical bond angle of π + *Δ* where *Δ* ≥ 0 is actually computed (*via*eqn (140)) to be a bond angle of *θ* = π − *Δ*.

**Fig. 6:**
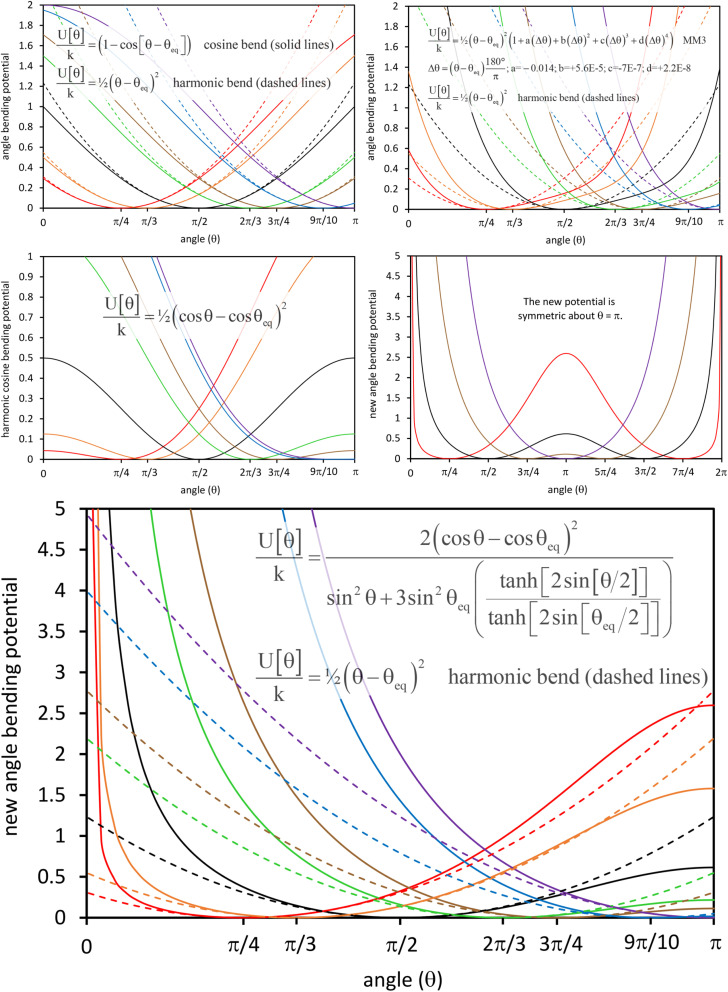
The new angle-bending potential has continuous derivatives of all orders even for an angle of π radians while retaining a harmonic-like shape around the equilibrium angle (*i.e.*, *d*^2^*U*/*dθ*^2^|_*θ*=*θ*_eq__ > 0) for all values of *θ*_eq_ > 0. As shown in the top two and middle left panels, common angle-bending potentials described in prior literature do not achieve this. The middle right panel shows the new potential is symmetric about *θ* = π. See the text for a complete description. The lines are colored as follows: *θ*_eq_ = π/4 (red), π/3 (orange), π/2 (black), 2π/3 (green), 3π/4 (brown), 9π/10 (blue), and π (purple).

If an angle-bending potential does not satisfy eqn (144), the consequent spurious force discontinuity at *θ* = π could potentially degrade the accuracy of trajectories computed using numerical integrators (*e.g.*, Verlet integration^139,140^) for molecular dynamics calculations. As shown in Fig. 6, the following currently used angle-bending potentials violate eqn (144) when *θ*_eq_ ≠ π:

146 *U*_harmonic_bend_[*θ*] = ½*k*(*θ* − *θ*_eq_)^2^

147 *U*_cosine_bend_[*θ*] = *k*(1 − cos[*θ* − *θ*_eq_])

148
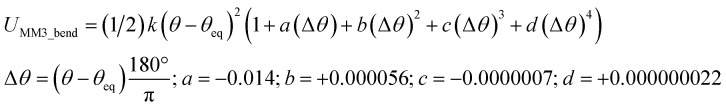
The MM3 bend parameters in eqn (148) are from Allinger's MM3-2000 parameter set, which uses an updated value of the parameter d compared to the original 1989 value of Lii and Allinger.^1,141^ The cosine bend in eqn (147) has not been widely used in forcefields to date; however, there have been some special cases of its use, especially for *θ*_eq_ → π.^23,142^

The harmonic cosine potential^142^

149 *U*_harmonic_cosine_[*θ*] = ½*k*(cos[*θ*] − cos[*θ*_eq_])^2^

obeys eqn (144) but suffers the drawback that the potential's curvature for *θ* → *θ*_eq_ is zero when *θ*_eq_ = π:150
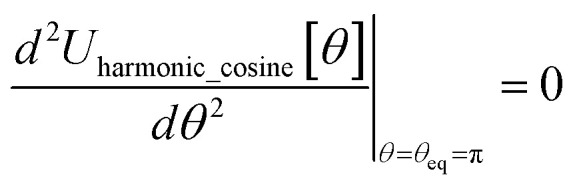
This means *U*_harmonic_cosine_ exhibits too weak restoring force for small displacements when *θ*_eq_ = π.^1^ As shown in Fig. 6, the potential energy curve for *U*_harmonic_cosine_[*θ*] is too flat under these conditions.

The practical consequence of the above problems is that flexible forcefields have often used different forms of angle-bending potentials for nearly linear angles (*i.e.*, *θ*_eq_ ≈ π) compared to significantly bent angles (*i.e.*, *θ*_eq_ ≪ π). For example, van der Spoel *et al.* introduced a special angle-bending potential applicable to only linear bond angles (*i.e.*, *θ*_eq_ = π).^143^ The DREIDING forcefield used the harmonic_cosine potential for bent angles (*i.e.*, *θ*_eq_ < π) and the cosine_bend potential for linear bond angles (*i.e.*, *θ*_eq_ = π).^142^ As another example, version 2 of the QuickFF protocol used the harmonic_bend potential for bent angles (*i.e.*, *θ*_eq_ < π) and the cosine_bend potential for linear bond angles (*i.e.*, *θ*_eq_ = π).^23^ These workarounds raise the additional question of how small (π − *θ*_eq_) should be to trigger the linear bond angle potential. For such potentials, *θ*_eq_ must be rounded to π to remove the force discontinuity at *θ* = π. For example, if *θ*_eq_ = 179°(π/180°) triggers the linear bond angle potential and gets rounded up to *θ̃*_eq_ = π, then this can introduce a small non-zero change in the optimized equilibrium geometry.

A new angle-bending potential is required to resolve these problems. Its form was derived as follows. Since

151 cos[*θ*] = cos[2π − *θ*]

it follows that eqn (145) is satisfied by choosing

152 *U*[*θ*] = func[cos[*θ*]]

If func[s] is an infinitely differentiable function with respect to the independent variable *s*, then *U*[*θ*] will be an infinitely differentiable function with respect to the independent variable *θ*.

The second derivative of the harmonic_cosine potential (eqn (149)) is153
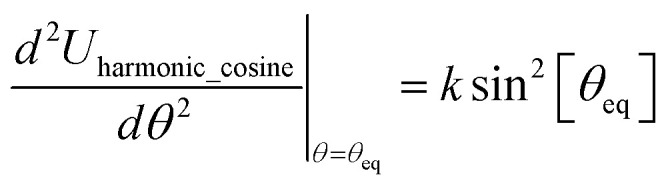
Therefore, the angle-bending potential's curvature at *θ* = *θ*_eq_ will equal its force constant *k* if we divide the harmonic_cosine potential by sin^2^[*θ*_eq_]:154
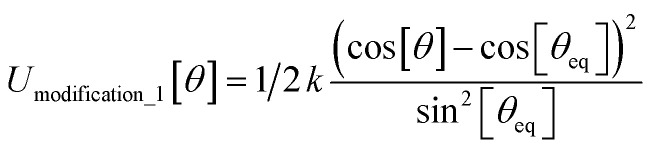


Unfortunately, this modified potential becomes infinite (aka ‘blows up’) when *θ*_eq_ = π, because *θ* ≠ *θ*_eq_ makes the numerator greater than zero while the denominator is zero when *θ*_eq_ = π. Close examination reveals this issue can be resolved by choosing155
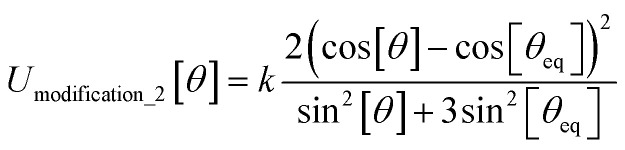
In the denominator, the 1 to 3 ratio of coefficients for sin^2^[*θ*] relative to sin^2^[*θ*_eq_] is required to make the potential's curvature equal to the force constant *k* at the equilibrium angle even when *θ*_eq_ = π:156
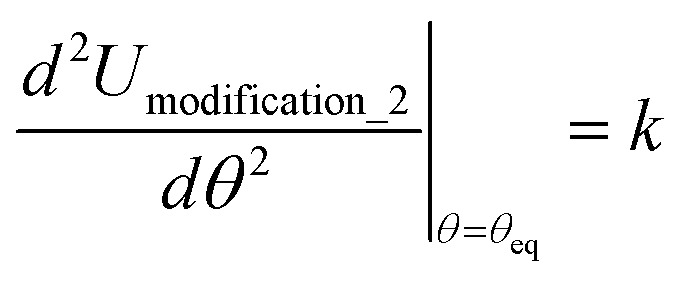


Eqn (156) holds for any possible value of the equilibrium angle 0 < *θ*_eq_ ≤ π. Eqn (155) has the deficiency that the restoring force approaches zero as *θ* approaches zero. Unfortunately, this means the modification_2 potential does not have sufficient repulsive force to prevent the bond angle from reaching *θ* = 0.

This problem is resolved by including a factor that makes the potential's denominator approach zero as *θ* approaches zero:157
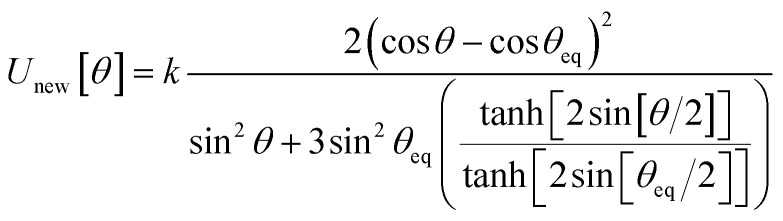
The denominator of eqn (157) includes the factor158
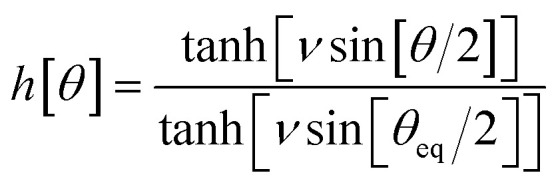
with the tanh multiplier *ν* having the specific value *ν* = 2. *h*[*θ*] has the following important limits:

159 *h*[*θ* = 0] = 0

160 *h*[*θ* = *θ*_eq_] = 1

161 *h*[*θ* > *θ*_eq_] > 1

162 *h*[*θ* < *θ*_eq_] < 1

163 *h*[*θ* = π] = tanh[*ν*]/tanh[*ν* sin[*θ*_eq_/2]]

Fig. 6 plots the new potential shown in eqn (157). Because this new potential approaches infinite value as *θ* approaches zero, it prevents a physical system with finite energy from reaching *θ* = 0. This behavior models the Pauli repulsion^129^ that prevents bond angles in a real physical system from reaching bond angles of *θ* = 0. Since164
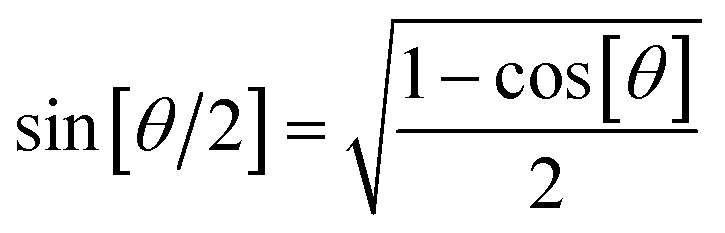
*U*_new_[*θ*] can be rewritten as a function of cos[*θ*]:165
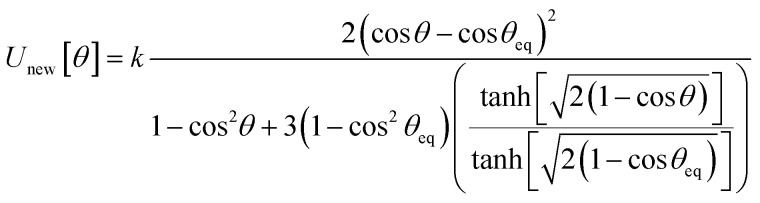


Accordingly, this new angle-bending potential has continuous well-defined derivatives of all orders for all 0 < *θ* ≤ π. ESI Section S4† gives the analytic first- and second-order derivatives of *U*_new_[*θ*].

As shown in Fig. 6, *U*_new_[*θ*] has the same function value, first derivative, and second derivative (curvature) as both *U*_harmonic_bend_[*θ*] and *U*_cosine_bend_[*θ*] at the energy minimum *θ* = *θ*_eq_. This leads to the following resolution. In every classical forcefield that uses *U*_harmonic_bend_[*θ*] and/or *U*_cosine_bend_[*θ*], the angle-bending potential can be upgraded to *U*_new_[*θ*] without requiring a change in the angle-bending force constant values. If a forcefield has been optimized to use *U*_new_[*θ*] but a molecular dynamics or Monte Carlo simulation program has not yet been updated to include this potential, in the meantime is it feasible to substitute either *U*_harmonic_bend_[*θ*] or *U*_cosine_bend_[*θ*] without requiring a change in the angle-bending force constant values, but in the long-term it is preferable to update the simulation code to use the more robust and general *U*_new_[*θ*] potential.

A potential argument against using *U*_new_[*θ*] is that its form is more complicated which will increase the computational costs during classical molecular dynamics and Monte Carlo simulations. However, a closer analysis shows the increased computational cost is likely to be insignificant in practical use, because the number of bonded interactions is typically much smaller than the number of non-bonded interactions during such simulations. Specifically, an atom-in-material A only shares bond angles with its first and second bonded neighbors, while it shares non-bonded interactions with a potentially much larger number of atoms within the non-bonded interaction cutoff distance (if used) or with all atoms in the entire simulation unit cell and their periodic images (if a non-bonded interaction cutoff distance is not used) except those within {excluded_A_}. Accordingly, the increased robustness and generality of this new angle-bending potential outweighs its relatively insignificant increased computational cost.

### 3.2 Computational results for real molecules

Table 4 summarizes the optimized geometries for ten triatomic molecules. CCSD calculations were performed in Gaussian 16 (ref. 95) using the def2-TZVPD^134^ basis set (In this article, CCSD not CCSD(T) calculations were used.). For molecules containing no elements heavier than neon, all electrons were correlated in the coupled-cluster calculation. For molecules containing one or more elements heavier than neon, the FreezeNobleGasCore keyword was used, which applies the coupled-cluster correlation to the valence shell electrons only on all atoms. Geometries were optimized to the following convergence criteria: (1) the maximum force is less than 0.00045 hartrees per bohr; (2) the root-mean squared (RMS) force is less than 0.0003 hartrees per bohr; (3) the maximum displacement is less than 0.0018 bohr; and (4) the RMS displacement is less than 0.0012 bohr.

**Table 4:** Optimized geometries for ten triatomic molecules

Molecule	Angle (°)	Bond length (Å)
CaH_2_ (HCaH)	180.0	2.065
CO_2_ (OCO)	180.0	1.157
HNO	108.4	1.056 (HN), 1.201(NO)
H_2_O (HOH)	104.7	0.962
Li_2_O (LiOLi)	180.0	1.619
NO_2_ (ONO)	135.0	1.185
NS_2_ (SNS)	154.1	1.543
SF_2_ (FSF)	97.7	1.586
SiH_2_ (HSiH) spin singlet	92.4	1.515
SO_2_ (OSO)	119.4	1.426

**Fig. 7:**
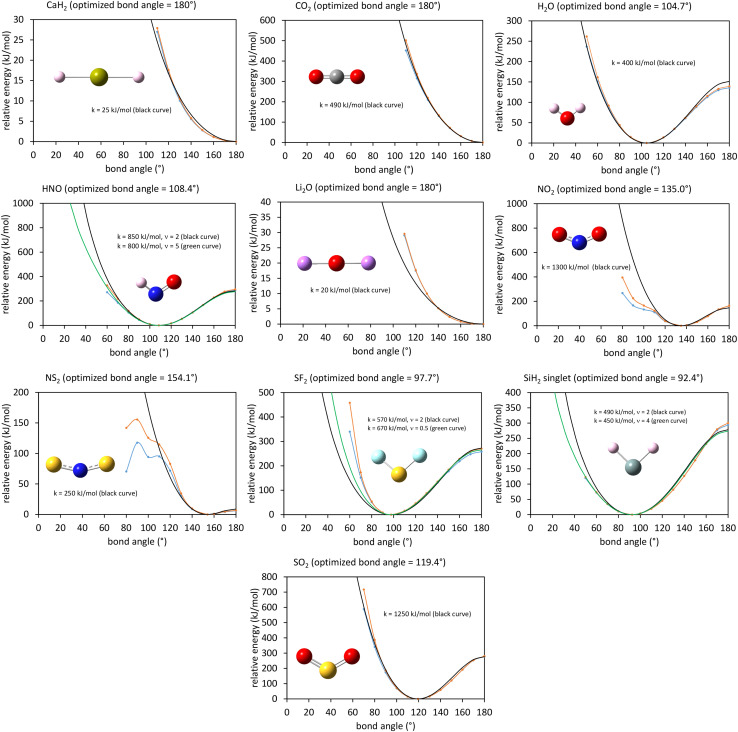
Angle-bending energy curves for ten triatomic molecules. The orange curves show the quantum-mechanically-computed (CCSD/def2-TZVPD) values holding the bond lengths fixed as the angle varied, while the blue curves (CCSD/def2-TZVPD) relaxed the bond lengths. The black curves show the new angle-bending model potential with the displayed force constant value. In some of the panels, green curves show modified model potentials. (For purposes of reporting the force constant values, radians were treated as dimensionless units.)

Fig. 7 compares the new angle-bending model potential to quantum-mechanically-computed angle-bending energy curves for these ten molecules. CCSD/def2-TZVPD energy curves were computed by varying the bond angle with and without relaxing the bond lengths. The settings for these calculations were similar to those described in the previous paragraph, except that some of the geometric parameters were constrained. As shown in Fig. 7, relaxing the bond lengths (blue curves) lowered the energy by only a small amount compared to keeping the bond lengths fixed (orange curves) as the constrained angle varied. The angle-bending force constant used in the model potential (black curves) is displayed on each graph. The particular value for the angle-bending force constant was chosen by visual inspection to achieve approximate agreement between the quantum-mechanically-computed and model potential energy curves.

Even though this model potential requires only a single parameter (*i.e.*, the force constant value) to be adjusted, it was generally in reasonable agreement with the quantum-mechanically-computed energy curves. Notably, this model potential reasonably matched the slope, height, and curvature of the quantum-mechanically-computed energy curve as the bond angle approached the limiting value *θ* = π. The reasons for this are understood. Specifically, the model potential has continuous well-defined derivatives of all orders over the entire range 0 < *θ* ≤ π, and the values of these derivatives change at reasonable rates. Eqn (145) imposes reflection symmetry about *θ* = π. The slope (*i.e.*, first derivative) varies from a value of zero at *θ* = *θ*_eq_ to a value of approximately *k*(*π* − *θ*_eq_)/3 at the midpoint *θ* = ½(*π* + *θ*_eq_) to a value of zero at *θ* = π. These constraints on the slope approximately determine the third derivative's values over this range of bond angles. Together with the function's value of zero at *θ* = *θ*_eq_ and the second-derivative's value of *k* at *θ* = *θ*_eq_, these various conditions approximately determine the curve's shape over the range of angles between *θ*_eq_ and *π*.

For SF_2_, the quantum-mechanically-computed energy curve rises more steeply than the model potential (eqn (157)) over the range *θ* < *θ*_eq_. This can be partially but not fully resolved by modifying the model potential in eqn (157) such that the tanh multiplier used is smaller (*e.g.*, 0.5) instead of 2. The result is plotted as the green curve in the SF_2_ panel of Fig. 7. For SF_2_, further reducing the tanh multiplier value towards zero does not result in significant improvement compared to the *ν* = 0.5 curve. For HNO, a slightly improved fit between the model potential and the quantum-mechanically-computed energy curve can be obtained by using a tanh multiplier value of 5 instead of 2 as shown by the green curve in the HNO panel of Fig. 7. For SiH_2_, a slightly improved fit between the model potential and the quantum-mechanically-computed energy curve can be obtained by using a tanh multiplier value of 4 instead of 2 as shown by the green curve in the SiH_2_ panel of Fig. 7. The tanh multiplier value of 2 (as shown in eqn (157)) was chosen as a compromise value that provides acceptably good results for most materials. Since sin[*θ*_eq_] = 0 when *θ*_eq_ = 180°, the value of the tanh multiplier *ν* has no impact on the model curves for CaH_2_, CO_2_, Li_2_O, and other triatomic molecules having *θ*_eq_ = 180°.

For NO_2_, the quantum-mechanically-computed energy curves have a shoulder in the range of 90 to 110°. This appears to be due to some chemical hybridization changes within the molecule (aka ‘chemical effects’) that are not captured by the model potential. For NS_2_, the quantum-mechanically-computed energy curves have a shoulder around 100° and a local maximum around 90°. This appears to be due to the formation of a S–S bond that lowers the energy as the bond angle is decreased to approximately 80°.

## 4. Flexibility parameters that approximately reproduce experimental vibrational frequencies

### 4.1 Homodiatomic molecules

Using the Manz stretch model potentials for H_2_ and O_2_ shown in Fig. 4, the following one-dimensional Schrodinger equation was solved for the vibrational eigenstates:166
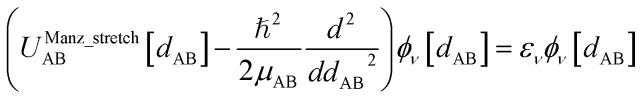
The reduced mass is defined as167
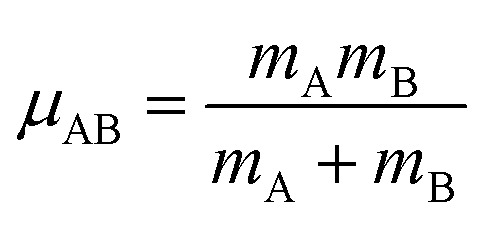
This Schrodinger equation corresponds to the situation in which the molecule is not rotating, its center-of-mass remains stationary, the Born–Oppenheimer approximation applies, relativistic effects are neglected, and the molecule is in the electronic ground state. The zero point energy (ZPE) corresponds to *ν* = 0, while *ν* = 1 is the first excited vibrational level.

I wrote a Matlab script to solve for the eigenvalues *ε*_*ν*_ and eigenfunctions *ϕ*_*ν*_[*d*_AB_] for *ν* = 0, 1, 2,…. This script and its output results are provided in the ESI.† This script used a uniform grid for (*d*^eq^_AB_ − 1.5 bohr) ≤ *d*_AB_ ≤ (*d*^eq^_AB_ + 5 bohr) with a grid spacing of 0.001 bohr. The second-derivative in eqn (166) was computed using the central finite difference approximation. Computational tests with a finer grid spacing (0.0005 bohr) and the slightly larger range (*d*^eq^_AB_ − 2 bohr) ≤ *d*_AB_ ≤ (*d*^eq^_AB_ + 6 bohr) changed the (*ε*_*ν*_ − *ε*_*ν*−1_) and ZPE results for H_2_ and O_2_ by less than 0.1 cm^−1^.

As shown in Table 5, the computed ZPE and first dozen excited vibrational levels for the H_2_ molecule differed by less than 10% from the experimental values. For different isotopes, the Born–Oppenheimer potential energy curve (and hence optimized force constant) remains the same, but the vibrational frequencies change owing to changes in reduced mass. Table 6 shows good agreement between the computed and experimental values for the first vibrational transition frequency of each hydrogen molecule isotope. As shown in Table 7, the model potential predicted the first 25 vibrational energy levels for the O_2_ molecule within 3% of the experimental values. The relative deviations became larger closer to the bond dissociation energy (*e.g.*, 7% error for the *ν*_29 ← 30_ transition of O_2_). Overall, these results show the Manz stretch model potential approximately reproduces experimental bond vibration frequencies.

**Table 5:** Comparison of vibrational frequencies (in wavenumber, cm^−1^) calculated using the Manz stretch model potential to experimental values for the H_2_ molecule. Each value is the energy of that vibrational level minus the energy of the prior vibrational level. ZPE = zero point energy

*υ*	Experiment^a^	Calculated	% error
ZPE	2179	2254	3%
1	4161	4312	4%
2	3926	4048	3%
3	3695	3782	2%
4	3468	3515	1%
5	3242	3247	0%
6	3014	2976	−1%
7	2782	2704	−3%
8	2543	2429	−4%
9	2293	2151	−6%
10	2026	1871	−8%
11	1737	1587	−9%
12	1415	1299	−8%

a Experimental data from ref. 144–146.

**Table 6:** Comparison of *υ*_0 ← 1_ transition frequency (in wavenumber, cm^−1^) calculated using the Manz stretch model potential to experimental values for different isotopes of the hydrogen molecule: H_2_, HD, D_2_, HT, DT, and T_2_

	Experiment	Calculated	% error
H_2_	4161 (ref. 145 and 147)	4312	4
HD	3632 (ref. 145 and 147)	3765	4
D_2_	2994 (ref. 145 and 147)	3104	4
HT	3435 (ref. 148)	3561	4
DT	2743 (ref. 148)	2845	4
T_2_	2465 (ref. 148)	2556	4

**Table 7:** Comparison of vibrational frequencies (in wavenumber, cm^−1^) calculated using the Manz stretch model potential to experimental values for the O_2_ molecule (^16^O isotope). Each listed value is the energy of that vibrational level minus the energy of the prior vibrational level. ZPE = zero point energy

*υ*	Experiment^a^	Calculated	% error
ZPE	787	761	−3%
1	1556	1506	−3%
2	1533	1484	−3%
3	1510	1463	−3%
4	1486	1441	−3%
5	1463	1420	−3%
6	1440	1398	−3%
7	1419	1377	−3%
8	1395	1355	−3%
9	1372	1334	−3%
10	1350	1312	−3%
11	1329	1290	−3%
12	1304	1269	−3%
13	1280	1247	−3%
14	1258	1225	−3%
15	1236	1203	−3%
16	1212	1181	−3%
17	1188	1160	−2%
18	1166	1138	−2%
19	1141	1116	−2%
20	1117	1094	−2%
21	1092	1072	−2%
22	1067	1050	−2%
23	1040	1028	−1%
24	1013	1006	−1%
25	985	983	0%
26	956	961	1%
27	925	939	2%
28	891	917	3%
29	858	894	4%
30	818	872	7%

a Experimental data as compiled in ref. 149.

### 4.2 Triatomic molecules

Why is it useful to compute both rigid and relaxed angle-bending scans as shown in Fig. 7? Comparing the rigid scan energy curve to the relaxed scan energy curve provides extremely valuable insights into the relative importance of some cross terms. Iff the relaxed scan curve is greatly below the rigid scan curve, then this indicates that changing bond lengths substantially lowers the energy at non-equilibrium angle values, and in this case bond-bend cross terms may be needed to construct an accurate forcefield. Iff the relaxed and rigid angle-scan curves are nearly identical, this suggests bond-bend cross terms are not required to construct an accurate forcefield model.

In this section, flexibility models were constructed for several triatomic molecules as examples, because these molecules do not require dihedral terms. Owing to the lengthy space required to thoroughly explain the dihedral terms, I decided that it would be easier for readers if the content related to dihedral model potentials is presented in a subsequent companion article rather than incorporating it here.

As shown in Table 8, several flexibility models were parameterized and compared for the CO_2_, water, HNO, and SO_2_ molecules. For each molecule in this set, Fig. 7 shows that the relaxed angle-scan energy curve is approximately the same as the rigid angle-scan energy curve for the same molecule. Consequently, bond-bend cross terms were not required to build accurate flexibility models for these molecules. The constructed flexibility models contained bond-stretch and angle-bend terms. Flexibility models with and without Urey–Bradley or bond–bond cross terms were compared. My new angle-bending model potential (eqn (157)) was used for all of these flexibility models. Both bond and Urey–Bradley stretches were modeled using either the harmonic stretch (eqn (10)) or Manz stretch (eqn (95)) model potential. When present, the bond–bond cross term had the form:

168 *U*^bond–bond^_ABC_ = *k*(*d*_AB_ − *d*^eq^_AB_)(*d*_BC_ − *d*^eq^_BC_)

**Table 8:** Flexibility parameters fitted for the CO_2_, H_2_O, HNO, and SO_2_ molecules. UB = Urey–Bradley term. BBC = bond–bond cross term. Entries marked with “—” indicate that type of term was not considered for inclusion in the model. Entries marked with “0” mean that type of term was considered but converged to a value of zero. Please see the main text for a list of *γ*° values. For each molecule, results for the recommended flexibility model are shown in boldface type

	Stretch type	*k*_stretch_ (eV bohr^−2^)	*k*_bend_ (eV)	*k*_UB_ (eV bohr^−2^)	*k*_BBC_ (eV bohr^−2^)	*R*-Squared training	*R*-Squared validation
CO_2_	Manz	30.58	5.17	—	—	0.9928	0.9940
**CO**_**2**_	**Manz**	**27.26**	**5.03**	**2.31**	**—**	**0.9995**	**0.9998**
CO_2_	Harmonic	31.58	5.17	—	—	0.9287	0.9911
CO_2_	Harmonic	28.15	5.02	2.86	—	0.9341	0.9982
CO_2_	Harmonic	31.01	5.17	—	2.86	0.9341	0.9929
**H**_**2**_ **O**	**Manz**	**14.95**	**4.26**	**—**	**—**	**0.9996**	**0.9974**
H_2_O	Manz	14.87	4.11	0.10	—	0.9996	0.9978
H_2_O	Harmonic	15.62	4.26	—	—	0.9456	0.9957
H_2_O	Harmonic	15.62	4.26	0.00	—	0.9456	0.9957
H_2_O	Harmonic	15.66	4.26	—	−0.16	0.9457	0.9957
**HNO**	**Manz**	**8.97 (HN), 22.34 (NO)**	**8.07**	**—**	**—**	**0.9902**	**0.9816**
HNO	Manz	8.55 (HN), 21.89 (NO)	6.23	0.61	—	0.9922	0.9751
HNO	Harmonic	9.03 (HN), 23.45 (NO)	7.99	—	—	0.9410	0.9787
HNO	Harmonic	7.47 (HN), 21.74 (NO)	4.07	2.50	—	0.9472	0.9907
HNO	Harmonic	9.03 (HN), 23.45 (NO)	8.00	—	1.71	0.9465	0.9814
SO_2_	Manz	20.34	11.60	—	—	0.9970	0.9948
**SO**_**2**_	**Manz**	**19.90**	**9.71**	**0.46**	**—**	**0.9986**	**0.9973**
SO_2_	Harmonic	20.86	11.56	—	—	0.9648	0.9934
SO_2_	Harmonic	19.88	9.42	1.11	—	0.9658	0.9943
SO_2_	Harmonic	20.83	11.56	—	0.16	0.9648	0.9934

The force constants (*i.e.*, *k* values) were the only adjustable parameters in these flexibility models. Nonadjustable parameters included the equilibrium lengths and equilibrium bond angle, which were taken from the CCSD/def2-TZVPD optimized geometries. For the Manz stretch potential, the *γ*° values computed using the method described in Section 2.6.2 were (in bohr^−1^): (a) 1.203 (C–O) and 1.257 (O–O) for CO_2_, (b) 1.276 (H–O) and 1.129 (H–H) for water, (c) 1.246 (H–N), 1.251 (N–O), and 1.212 (H–O) for HNO, and (d) 1.079 (S–O) and 1.151 (O–O) for SO_2_. *γ*° between the two outer atoms was only relevant when the flexibility model contained Urey–Bradley interaction.

The training and validation datasets contained quantum chemistry calculations at the CCSD/def2-TZVPD level of theory. For each molecule, the training dataset contained:

(1) The QM optimized geometry and energy.

(2) Both the relaxed angle-scan and rigid angle-scan geometries and energies for the subset of angles satisfying (*θ*^eq^_ABC_ − 30°) ≤ *θ*_ABC_ ≤ (*θ*^eq^_ABC_ + 30°). This subset of angles was chosen, because it focused the fit on angle values that are not extremely far away from *θ*^eq^_ABC_. The specific datapoints used were those plotted in Fig. 7 that satisfied the additional condition that they were within this angle range.

(3) QM-computed single-point energies for a set of geometries in which each bond length was changed by −0.14, −0.07, 0.00, +0.07, +0.14 Å relative to the fully-relaxed unconstrained geometry. For these structures, the bond angle was held rigid at *θ*^eq^_ABC_. For each structure, the single-point energy was computed without constrained geometry relaxation. For a symmetric triatomic (*e.g.*, CO_2_, H_2_O, SO_2_), this yielded 14 distinct displaced geometries. For HNO, this yielded 24 distinct displaced geometries.

The GRG solver in Excel was used to solve this linear regression problem that minimizes the least-squares loss function shown in eqn (138) subject to the following force constant bounds. The angle-bending, bond stretch, and Urey–Bradley stretch (if present) force constants were constrained to be non-negative. No bounds were placed on the bond–bond cross (if present) force constant.

For each molecule, the validation dataset was constructed by using an uniform random number generator to generate random bond displacements in the interval −0.07 to +0.07 Å and random angle displacements in the interval −30 to +30° relative to the optimized geometry. In each validation geometry, three separate random numbers were used to independently displace each of the two bonds and the angle. For each molecule, nine validation geometries were prepared in this manner.

*R*-Squared values for the training and validation datasets were then computed using eqn (132)–(134). Table 8 lists the optimized force constant values, *R*-squared training, and *R*-squared validation.

For each flexibility model, normal vibrational mode analysis within the harmonic oscillator approximation was performed by diagonalizing the mass-weighted Hessian (MWH) matrix expressed in Cartesian coordinates:169
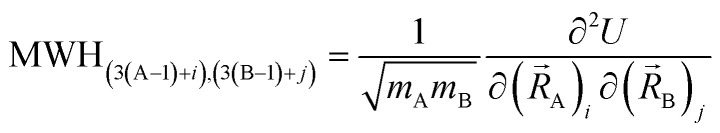
where *m*_A_ is the mass of atom A. Here, 
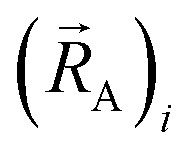
 for *i* ∈ {1, 2, 3} denotes the X, Y, or Z component of the nuclear position 
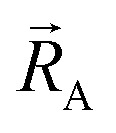
. The second derivatives can be computed either analytically or numerically; here, they were computed numerically using the central finite difference approximation. The eigenvalues {*λ*_*i*_} of the MWH matrix are related to the normal mode frequencies {freq_*i*_} *via*:^101^170
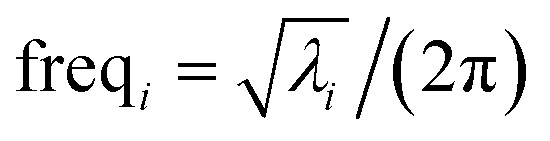


Each normal mode frequency was converted to wavenumber by dividing by the speed of light, *c*. Each eigenvector 
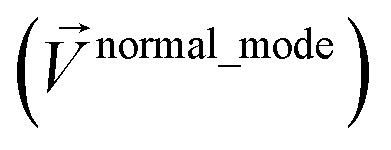
 of the MWH matrix is the corresponding normal mode's mass-weighted differential displacement vector:171
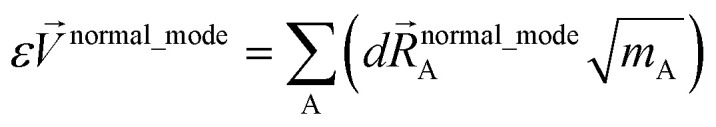
for infinitesimal |*ε*|.

For linear molecules, five of the MWH eigenvalues are zero; these correspond to molecular rotation (2 modes) and center-of-mass translation (3 modes). For nonlinear molecules, six of the MWH eigenvalues are zero; these correspond to molecular rotation (3 modes) and center-of-mass translation (3 modes).

Table 9 lists the computed vibrational frequencies (in wavenumber, cm^−1^) and their percent errors relative to experimental reference values. Examining Tables 8 and 9, flexibility models using the Manz stretch potential performed slightly better than those using the harmonic stretch potential. However, all of the parameterized flexibility models performed reasonably well. Including a Urey–Bradley term improved the results for CO_2_ and SO_2_ but had little effect for H_2_O and HNO. Including a bond–bond cross term had little effect. In Tables 8 and 9, the ‘recommended’ flexibility model shown in boldface type achieves a good combination of high *R*-squared validation, high *R*-squared training, and accuracy for computed frequencies, while not introducing an excessive number of force constants.

**Table 9:** Computed vibrational frequencies (in wavenumber, cm^−1^) using different forcefields for the CO_2_, H_2_O, HNO, and SO_2_ molecules. For CO_2_, the bend mode is 2-fold degenerate. The percent error relative to experimentally-measured value (ref. 150) is shown in parentheses. For each molecule, results for the recommended flexibility model are shown in boldface type

	Stretch type	UB?	BBC?	Bend	Stretch # 1^a^	Stretch # 2^a^
CO_2_	Manz	No	No	694 (4%)	1363 (2%)	2609 (11%)
**CO**_**2**_	**Manz**	**Yes**	**No**	**684 (3%)**	**1391 (4%)**	**2463 (5%)**
CO_2_	Harmonic	No	No	694 (4%)	1385 (4%)	2651 (13%)
CO_2_	Harmonic	Yes	No	684 (3%)	1434 (8%)	2503 (7%)
CO_2_	Harmonic	No	Yes	694 (4%)	1434 (8%)	2503 (7%)
**H**_**2**_ **O**	**Manz**	**No**	**No**	**1634 (2%)**	**3885 (6%)**	**3942 (5%)**
H_2_O	Manz	Yes	No	1629 (2%)	3889 (6%)	3932 (5%)
H_2_O	Harmonic	No	No	1633 (2%)	3972 (9%)	4030 (7%)
H_2_O	Harmonic	Yes	No	1633 (2%)	3972 (9%)	4030 (7%)
H_2_O	Harmonic	No	Yes	1633 (2%)	3956 (8%)	4055 (8%)
**HNO**	**Manz**	**No**	**No**	**1451 (-3%)**	**3047 (14%)**	**1776 (13%)**
HNO	Manz	Yes	No	1407 (−6%)	3032 (13%)	1723 (10%)
HNO	Harmonic	No	No	1453 (−3%)	3058 (14%)	1807 (15%)
HNO	Harmonic	Yes	No	1434 (−4%)	3051 (14%)	1714 (10%)
HNO	Harmonic	No	Yes	1455 (−3%)	3051 (14%)	1798 (15%)
SO_2_	Manz	No	No	550 (6%)	1259 (9%)	1468 (8%)
**SO**_**2**_	**Manz**	**Yes**	**No**	**529 (2%)**	**1255 (9%)**	**1452 (7%)**
SO_2_	Harmonic	No	No	549 (6%)	1275 (11%)	1487 (9%)
SO_2_	Harmonic	Yes	No	553 (7%)	1272 (11%)	1452 (7%)
SO_2_	Harmonic	No	Yes	549 (6%)	1279 (11%)	1480 (9%)

a For CO_2_, H_2_O and SO_2_, stretch # 1 is the symmetric stretch, and stretch # 2 is the asymmetric stretch. For HNO, stretch # 1 is the H–N stretch, and stretch # 2 is the N–O stretch.

For CO_2_, the energy splitting between the asymmetric stretch and the symmetric stretch was not predominantly due to Urey–Bradley or bond–bond cross interactions as mistakenly suggested in the companion article.^45^ That suggestion was based on the observation that without Urey–Bradley or bond–bond cross terms, the Hessian matrix is already diagonal (with equal eigenvalues for the two bond stretches) when expressed in terms of the internal coordinates (*d*_AB_, *d*_BC_, *θ*_ABC_) as:^45^172
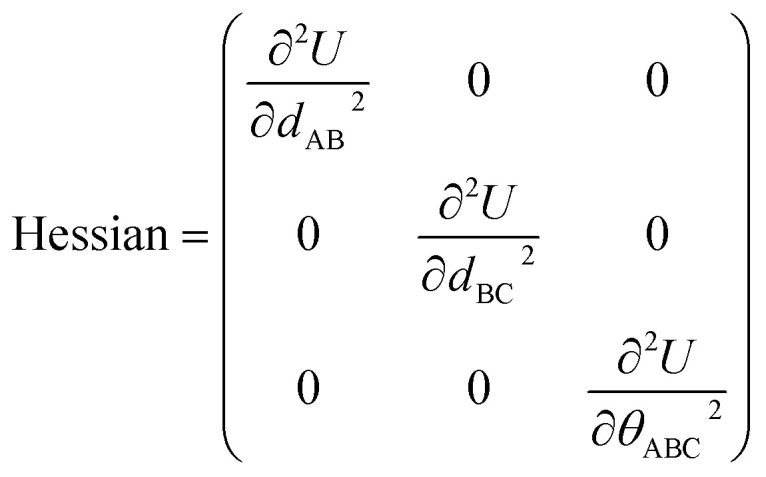
However, the normal vibrational modes must be computed by diagonalizing the Hessian matrix defined by mass-weighted Cartesian coordinates, as shown in eqn (169).^101^ For CO_2_, this mass-weighted Hessian contains some non-zero off-diagonal elements even when the flexibility model contains no Urey–Bradley or bond–bond cross interactions, and this leads to a splitting between asymmetric and symmetric stretch frequencies even if no Urey–Bradley or bond–bond cross interactions are contained in the flexibility model.

## 5. Conclusions

In this article, I derived theoretical foundations of force field functional theory (FFFT). FFFT studies topics related to the functional representation of nonreactive forcefields to achieve various desirable properties such as:

(a) Formal exactness of the forcefield's energy functional under certain conditions.

(b) A formally exact ansatz separating the bonded potential energy from the nonbonded potential energy within a bonded cluster in a way that enables bonded parameters to be optimized using linear regression instead of requiring nonlinear regression.

(c) The potential energy's continuous differentiability to various orders with respect to energetically accessible internal coordinate displacements within a subdomain defined by one electronic ground state.

(d) Forcefield design that guarantees the reference ground-state geometry is exactly reproduced as an equilibrium structure on the forcefield's potential energy landscape.

(e) Reasonably accurate and broadly applicable frugal model potentials.

(f) Computationally efficient embedded feature selection that identifies and removes unimportant forcefield terms.

(g) Well-designed methods to parameterize the forcefield from quantum-mechanically-computed and (optionally) experimental reference data.

(h) Forcefields that approximately reproduce experimentally-measured properties.

Theoretical foundations of items (a), (b), and (d) were derived in Sections 2.1–2.5 above and demonstrated with examples in Sections 2.6, 4.1, and 4.2. Examples of (e) frugal model potentials include my new angle-bending and bond stretch model potentials that have (c) continuous differentiability to all orders. A companion article describes and applies several (f) embedded feature selection techniques including dihedral pruning, dihedral mode smart selection, and LASSO regression to identify and remove unimportant forcefield terms.^45^ A companion article performs (g) quantum-mechanically-derived forcefield parameterization for more than a hundred MOFs that (h) exactly reproduces the experimental lattice constants.^45^

In general, a forcefield's potential energy is a functional of the material's chemical geometry and externally applied fields (if any) that exactly or approximately matches the quantum-mechanically-computed Born–Oppenheimer electronic energy as shown in eqn (14) or (88). This Born–Oppenheimer electronic energy surface is composed of subdomains such that chemical geometries within the same subdomain share the similar electronic ground state. Within each subdomain, 
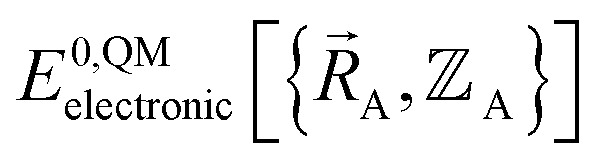
 has continuous first-order derivatives with respect to changes in the atomic coordinates 
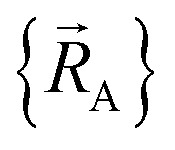
; however, its first (and/or higher-order) derivatives may be discontinuous at boundaries where two or more subdomains (*i.e.*, two or more different electronic ground states) intersect in energy. As shown in eqn (16), the system's total energy is obtained by adding the nuclear kinetic energy to the potential energy.

For convenience, 
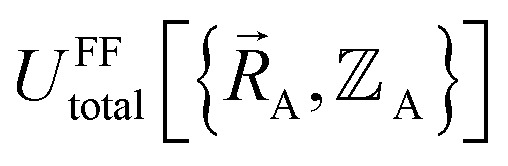
 is often partitioned into bonded and nonbonded interactions. In this article, I showed how to construct such a partition in a way that always guarantees the reference ground-state geometry of an isolated bonded cluster is exactly reproduced as a stationary point on the forcefield's potential energy landscape independently of the particular values to be assigned to the forcefield's force constants. At this optimized geometry, the new scheme's bonded interaction terms completely account for the isolated bonded cluster's geometry, potential energy, forces (first derivatives of potential energy), and Hessian (second derivatives of potential energy) with non-bonded interactions affecting only higher-order derivatives. This partitioning scheme is formally exact, because it does not introduce any new approximations into the forcefield model. In this partitioning scheme, the so-called ‘resting values’ contained in each flexibility term are precisely equal to the equilibrium values from the material's quantum-mechanically-computed ground-state geometry. Because these equilibrium values can be computed directly and do not need to be fitted during the forcefield parameterization, this transforms the task of optimizing the forcefield's bonded parameters from a nonlinear regression problem into a linear regression problem. Because linear regression problems are convex, this prevents separated regions in the optimization landscape from containing different local minima that can trap the optimizer. Moreover, multicollinearity issues can be more easily resolved (*e.g.*, by using the LASSO^32,33^ method) in linear regression compared to nonlinear regression.

A key advantage of this new ansatz for separating intracluster nonbonded interactions from bonded interactions is that it reduces the sensitivity of optimized values for the bonded parameters on the particular choice of nonbonded interaction model. This allows the bonded interaction terms to be optimally parameterized to leading order without having to first choose specific values for the nonbonded interaction parameters. As an example, these important features were clearly demonstrated for the C–F bond stretch in the C_6_F_6_ molecule.

Section 2.5 above discusses the particular conditions that must be satisfied for 
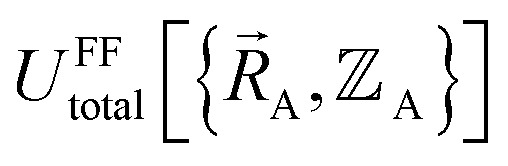
 to exactly equal 
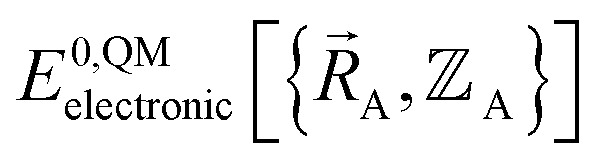
. Formal exactness requires that the forcefield was parameterized for the exact system being studied by the forcefield. The formally exact nonreactive forcefield requires a full series expansion of 
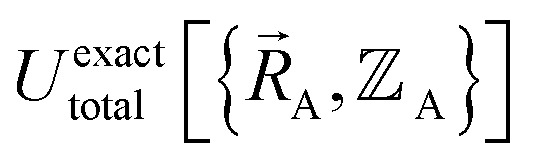
 in terms of the material's internal coordinates; however, in most practical applications the explicit form of this exact expansion is unknown. In most practical applications, 
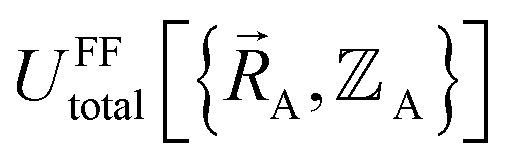
 is represented by a weighted sum of model potentials to provide a pragmatic approximation of 
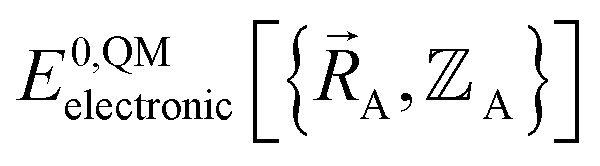
. The coefficients in front of these model potentials are called force constants.

In practice, the formally exact series expansion is normally truncated by using model potentials having a finite number of interaction terms. This truncation introduces approximation. Careful choice of the model potentials can yield high computational efficiency, a relatively small number of required flexibility terms, continuous derivatives of all orders with respect to energetically accessible atom-in-material displacements, and excellent accuracy. This article introduced new angle-bending and bond-stretch model potentials that require only a small number of terms to achieve excellent accuracy, high computational efficiency, and continuous derivatives of all orders with respect to atom-in-material displacements.

The new angle-bending model potential was carefully derived to capture correct dynamics across a wide range of bond angles including the limiting value of *θ* = π. In contrast, most previously used angle-bending model potentials have either a derivative discontinuity or incorrect dynamics when the bond angle reaches *θ* = π. This new angle-bending model potential was compared to CCSD/def2-TZVPD quantum-mechanically-computed energy curves for ten triatomic molecules: CaH_2_, CO_2_, H_2_O, HNO, Li_2_O, NO_2_, NS_2_, SF_2_, SiH_2_, and SO_2_. In all ten cases, the new angle-bending potential provided reasonably good results. However, some moderate discrepancies for *θ* < *θ*_eq_ were observed for NS_2_ (due to chemical bonding changes), NO_2_ (due to chemical hybridization changes), and SF_2_ (due to steric repulsion between the two F atoms).

The new bond-stretch model potential was derived using first principles. This provides the key advantage that its exponent 
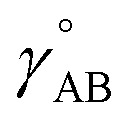
 is directly quantum-mechanically computed. This new bond-stretch model potential provides excellent accuracy for many bonds across a wide range of Δ*d*_AB_ = *d*_AB_ − *d*^eq^_AB_ values, even as the bond length is stretched to infinity. Remarkably, this is accomplished with only one empirically-fitted parameter, which is the bond's force constant (*k*_stretch_).

In this work, complete flexibility models (*i.e.*, bonded interaction models) were constructed for the H_2_, O_2_, CO_2_, water, HNO, and SO_2_ molecules. For each of these molecules, vibrational frequencies predicted by the parameterized flexibility model agreed closely with previously published experimentally-measured frequencies. For H_2_ and O_2_, these parameterized flexibility models agreed closely with the quantum-mechanically-computed bond-energy-*versus*-bond-distance curve. For CO_2_, water, HNO, and SO_2_, these parameterized flexibility models gave excellent *R*-squared values for approximately reproducing the quantum-mechanically-computed energies of independently chosen sets of validation geometries.

In a companion article, this new theory was used to optimize bonded parameters (aka flexibility parameters) for 116 MOFs.^45^ As shown in that article, flexible forcefields constructed using FFFT and my new angle-bending and dihedral torsion model potentials gave excellent performance. Specifically, the model-predicted forces yielded goodness-of-fit (*R*-squared values) of 0.910 (avg across all MOFs) ± 0.018 (st. dev.) for atom-in-material forces across a quantum-mechanically-computed validation set of geometries generated using *ab initio* molecular dynamics in the NVE ensemble, where the parameterized forcefield model used dihedral pruning, individual equilibrium values, and no bond–bond cross terms.^45^ This clearly demonstrates FFFT has enormous practical utility. That companion article introduces new best practices for: (a) typing bonds, angles, dihedrals, and other internal coordinates, (b) pruning dihedrals to reduce the redundancy of internal coordinates, (c) using the LASSO method in least-squares regression of the force constants to identify and eliminate unimportant forcefield terms, and (d) designing the forcefield to exactly reproduce experimental lattice constants defining the material's unit cell. That article introduces the well-designed SAVESTEPS protocol to parameterize the forcefield's bonded terms from quantum-mechanically-computed reference data.

## Data availability

Optimized geometries of molecules, data analysis spreadsheets, Matlab codes and results, and outputs of the calculate_Manz_and_Morse_stretch_potential_exponents program are included as part of the ESI.† The calculate_Manz_and_Morse_stretch_potential_exponents program is available for download from http://ddec.sourceforge.net.

## Conflicts of interest

There are no conflicts of interest to declare.

## Supplementary Material

RA-014-D4RA01861C-s001
