# Supplementary material for: A formally exact theory to construct nonreactive forcefields using linear regression to optimize bonded parameters
Source: RSC Adv. 2024 Oct 22;14(45):33345–83. doi: 10.1039/d4ra01861c (PMC11494935; doi:10.1039/d4ra01861c)
Supplement: RA-014-D4RA01861C-s001 [file RA-014-D4RA01861C-s001.pdf]

Supplementary Information (SI) for RSC Advances.
This journal is © The Royal Society of Chemistry 2024

Electronic Supplementary Information for

A Formally Exact Theory to Construct Nonreactive Forcefields using Linear Regression to Optimize Bonded Parameters

Thomas A. Manz

Chemical & Materials Engineering, New Mexico State University, Las Cruces, NM 88001
Email: tmanz@nmsu.edu

Contents

1. Supplementary tables

2. Analytic derivatives of the damped nonbonded potential

3. Analytic derivatives of the Manz stretch potential

4. Analytic derivatives of my new angle-bending potential

S1. Supplementary tables

Table S1: Comparison of quantum-mechanically-computed Born-Oppenheimer energies (FCI/aug-cc-pVQZ) to those of the fitted forcefield for the H_2 singlet molecule. Results are shown for different values of the LASSO regularization parameter λ. The last row shows the root-mean-squared error (RMSE) in hartree. Please see Section 2.6.1 of the main text for additional details and discussion.

length (pm)	$E_{singlet}$ (hartree)	$E_{triplet}$ (hartree)	U^{FF}-$E_{singlet}$ ($\lambda=10^{-8}$)	U^{FF}-$E_{singlet}$ ($\lambda=10^{-9}$)	U^{FF}-$E_{singlet}$ ($\lambda=10^{-10}$)	U^{FF}-$E_{singlet}$ ($\lambda=10^{-11}$)	U^{FF}-$E_{singlet}$ ($\lambda=10^{-12}$)	U^{FF}-$E_{singlet}$ ($\lambda=10^{-20}$)
50	-1.10342	-0.57329	-0.00003	-0.00001	0.00000	0.00000	0.00000	0.00000
60	-1.15536	-0.69147	0.00015	0.00008	0.00002	-0.00001	-0.00002	0.00000
70	-1.17259	-0.76151	0.00000	0.00001	0.00001	0.00001	0.00001	-0.00002
74.199	-1.17387	-0.78459	0.00000	0.00000	0.00000	0.00000	0.00000	0.00000
80	-1.17190	-0.81221	-0.00002	0.00001	0.00001	0.00002	0.00002	-0.00002
90	-1.16189	-0.85111	-0.00017	0.00001	0.00003	0.00006	0.00007	0.00000
100	-1.14725	-0.88188	-0.00029	-0.00007	-0.00002	0.00002	0.00003	0.00005
125	-1.10531	-0.93467	0.00008	-0.00007	-0.00005	-0.00008	-0.00009	-0.00004
150	-1.06790	-0.96483	0.00036	0.00015	0.00010	0.00009	0.00008	0.00002
175	-1.04010	-0.98161	-0.00001	0.00004	0.00002	0.00004	0.00005	0.00005
200	-1.02193	-0.99066	-0.00034	-0.00014	-0.00010	-0.00009	-0.00008	-0.00004
225	-1.01130	-0.99539	-0.00027	-0.00010	-0.00006	-0.00006	-0.00006	-0.00005
250	-1.00558	-0.99779	0.00002	0.00003	0.00005	0.00004	0.00003	0.00001
275	-1.00268	-0.99896	0.00025	0.00011	0.00009	0.00008	0.00007	0.00004
300	-1.00125	-0.99951	0.00029	0.00008	0.00003	0.00003	0.00004	0.00003
350	-1.00023	-0.99986	0.00001	-0.00008	-0.00011	-0.00009	-0.00008	-0.00004
400	-0.99999	-0.99991	-0.00025	-0.00008	-0.00005	-0.00005	-0.00005	-0.00004
450	-0.99998	-0.99991	-0.00014	0.00008	0.00013	0.00011	0.00010	0.00006
500	-0.99991	-0.99991	0.00017	-0.00002	-0.00005	-0.00005	-0.00004	-0.00002
RMSE	—	—	2.0E-04	7.6E-05	6.3E-05	5.9E-05	5.8E-05	3.4E-05

Table S2: Computed force constants representing the H_2 electronic singlet state's bonded interaction energy. Results are shown for different values of the LASSO regularization parameter λ. The last row shows the sum of absolute values of the force constants. Please see Section 2.6.1 of the main text for additional details and discussion.

	$\lambda=10^{-8}$	$\lambda=10^{-9}$	$\lambda=10^{-10}$	$\lambda=10^{-11}$	$\lambda=10^{-12}$	$\lambda=10^{-20}$
k_1	0.722095562	0.732963059	0.731056623	0.732239879	0.732721173	0.716352037
k_2	-0.493924304	-0.453665595	-0.437217721	-0.425469207	-0.422810820	-0.401964886
k_3	0.423826705	0.172199834	0.203826522	0.164324310	0.152145226	0.620093926
k_4	0.000000000	-0.220855956	-0.436476147	-0.552373758	-0.576139531	-1.588700146
k_5	0.000000000	1.547859193	1.439235648	1.755529569	1.830273684	-0.723790820
k_6	-1.370826079	-2.221089984	-1.326968080	-1.099867066	-1.070479469	6.738112940
k_7	0.000000000	-0.659979520	-0.700716437	-1.128691139	-1.116208404	-3.372565479
k_8	0.000000000	0.000000000	-1.068785502	-1.502396056	-1.627598580	-4.246902958
k_9	0.000000000	0.000000000	0.000000000	-0.249227598	-0.529246504	-5.135999922
k_{10}	0.000000000	0.000000000	0.000000000	0.383964516	0.554896362	-0.243272935
k_{11}	1.404363463	0.000000000	0.992454504	1.802334706	1.728244142	4.554823776
k_{12}	0.158317729	2.789790589	1.532480608	1.860919841	2.130693142	7.914267812
k_{13}	0.000000000	0.000000000	1.120819449	0.963001977	1.684902249	6.919472380
k_{14}	0.000000000	0.000000000	0.000000000	0.000000000	0.392860928	1.508486184
k_{15}	0.000000000	0.000000000	0.000000000	-0.013939077	-1.083956753	-6.637173720
k_{16}	0.000000000	0.000000000	0.000000000	-1.585807685	-2.432520787	-12.808342789
k_{17}	0.000000000	0.000000000	0.000000000	-1.349541481	-2.005604045	-9.225503143
k_{18}	-0.754863080	-1.915303544	-2.374382200	0.007312916	1.475379887	15.592033023
abs sum	5.32821692	10.71370727	12.36441944	15.57694078	21.54668169	88.94785887

S2. Analytic derivatives of the damped nonbonded potential

When evaluating derivatives for Cases #1, #2, and #3 described below, the following derivatives of the separation functions are useful:

$$\tau_{AB}\left[d_{AB}, d_{cutoff}^{nonbonded}\right] = \tanh\left[\frac{d_{cutoff}^{nonbonded}}{d_{AB}} - \frac{d_{AB}}{d_{cutoff}^{nonbonded}}\right] \quad (S1)$$

$$\tau_{AB}\left[d_{AB}, d_{AB}^{eq\_j}\right] = \tanh\left[\frac{d_{AB}^{eq\_j}}{d_{AB}} - \frac{d_{AB}}{d_{AB}^{eq\_j}}\right] \quad (S2)$$

$$\vec{\nabla}_{C\notin\{A,B\}}\tau_{AB}\left[d_{AB}, d_{cutoff}^{nonbonded}\right] = 0 \quad (S3)$$

$$\vec{\nabla}_{C\notin\{A,B\}}\tau_{AB}\left[d_{AB}, d_{AB}^{eq\_j}\right] = 0 \quad (S4)$$

$$\vec{\nabla}_{atom\_1}\vec{\nabla}_{C\notin\{A,B\}}\tau_{AB}\left[d_{AB}, d_{cutoff}^{nonbonded}\right] = 0 \quad \text{for any atom\_1} \quad (S5)$$

$$\vec{\nabla}_{C\notin\{A,B\}}\vec{\nabla}_{atom\_2}\tau_{AB}\left[d_{AB}, d_{cutoff}^{nonbonded}\right] = 0 \quad \text{for any atom\_2} \quad (S6)$$

$$\vec{\nabla}_{atom\_1}\vec{\nabla}_{C\notin\{A,B\}}\tau_{AB}\left[d_{AB}, d_{AB}^{eq\_j}\right] = 0 \quad \text{for any atom\_1} \quad (S7)$$

$$\vec{\nabla}_{C\notin\{A,B\}}\vec{\nabla}_{atom\_2}\tau_{AB}\left[d_{AB}, d_{AB}^{eq\_j}\right] = 0 \quad \text{for any atom\_2} \quad (S8)$$

$$\vec{\nabla}_{A}\tau_{AB}\left[d_{AB}, d_{cutoff}^{nonbonded}\right] = \left(\frac{d_{cutoff}^{nonbonded}}{d_{AB}{}^{2}} + \frac{1}{d_{cutoff}^{nonbonded}}\right)\left(1-\tanh^{2}\left[\frac{d_{cutoff}^{nonbonded}}{d_{AB}} - \frac{d_{AB}}{d_{cutoff}^{nonbonded}}\right]\right)\hat{R}_{AB} \quad (S9)$$

$$\hat{R}_{AB} = \left(\vec{R}_{B} - \vec{R}_{A}\right)\Big/ d_{AB} \quad (S10)$$

$$\vec{\nabla}_{B}\tau_{AB}\left[d_{AB}, d_{cutoff}^{nonbonded}\right] = -\vec{\nabla}_{A}\tau_{AB}\left[d_{AB}, d_{cutoff}^{nonbonded}\right] \quad (S11)$$

$$\vec{\nabla}_{A}\tau_{AB}\left[d_{AB}, d_{AB}^{eq\_j}\right] = \left(\frac{d_{AB}^{eq\_j}}{d_{AB}{}^{2}} + \frac{1}{d_{AB}^{eq\_j}}\right)\left(1-\tanh^{2}\left[\frac{d_{AB}^{eq\_j}}{d_{AB}} - \frac{d_{AB}}{d_{AB}^{eq\_j}}\right]\right)\hat{R}_{AB} \quad (S12)$$

$$\vec{\nabla}_{B}\tau_{AB}\left[d_{AB}, d_{AB}^{eq\_j}\right] = -\vec{\nabla}_{A}\tau_{AB}\left[d_{AB}, d_{AB}^{eq\_j}\right] \quad (S13)$$

$$\vec{\nabla}_{A}\vec{\nabla}_{A}\tau_{AB}\left[d_{AB}, d_{cutoff}^{nonbonded}\right] = \vec{\nabla}_{B}\vec{\nabla}_{B}\tau_{AB}\left[d_{AB}, d_{cutoff}^{nonbonded}\right] =$$
$$\left(\begin{array}{l} \left(3\frac{d_{cutoff}^{nonbonded}}{d_{AB}{}^{3}} + \frac{1}{d_{cutoff}^{nonbonded} d_{AB}}\right)\left(1-\tanh^{2}\left[\frac{d_{cutoff}^{nonbonded}}{d_{AB}} - \frac{d_{AB}}{d_{cutoff}^{nonbonded}}\right]\right)\hat{R}_{AB}\hat{R}_{AB} \\ -2\left(\frac{d_{cutoff}^{nonbonded}}{d_{AB}{}^{2}} + \frac{1}{d_{cutoff}^{nonbonded}}\right)^{2}\tanh\left[\frac{d_{cutoff}^{nonbonded}}{d_{AB}} - \frac{d_{AB}}{d_{cutoff}^{nonbonded}}\right]\left(1-\tanh^{2}\left[\frac{d_{cutoff}^{nonbonded}}{d_{AB}} - \frac{d_{AB}}{d_{cutoff}^{nonbonded}}\right]\right)\hat{R}_{AB}\hat{R}_{AB} \\ -\frac{1}{d_{AB}}\left(\frac{d_{cutoff}^{nonbonded}}{d_{AB}{}^{2}} + \frac{1}{d_{cutoff}^{nonbonded}}\right)\left(1-\tanh^{2}\left[\frac{d_{cutoff}^{nonbonded}}{d_{AB}} - \frac{d_{AB}}{d_{cutoff}^{nonbonded}}\right]\right)\vec{\vec{\delta}} \end{array}\right) \quad (S14)$$

where $\vec{\vec{\delta}}$ is the 3×3 identity tensor.

$$\vec{\nabla}_{A}\vec{\nabla}_{B}\tau_{AB}\left[d_{AB}, d_{cutoff}^{nonbonded}\right] = \vec{\nabla}_{B}\vec{\nabla}_{A}\tau_{AB}\left[d_{AB}, d_{cutoff}^{nonbonded}\right] = -\vec{\nabla}_{A}\vec{\nabla}_{A}\tau_{AB}\left[d_{AB}, d_{cutoff}^{nonbonded}\right] \quad (S15)$$

$$\vec{\nabla}_A\vec{\nabla}_A\tau_{AB}\left[d_{AB},d_{AB}^{eq\_j}\right]=\vec{\nabla}_B\vec{\nabla}_B\tau_{AB}\left[d_{AB},d_{AB}^{eq\_j}\right]=$$
$$\begin{pmatrix} \left(3\frac{d_{AB}^{eq\_j}}{d_{AB}^{\ 3}}+\frac{1}{d_{AB}^{eq\_j}d_{AB}}\right)\left(1-\tanh^2\left[\frac{d_{AB}^{eq\_j}}{d_{AB}}-\frac{d_{AB}}{d_{AB}^{eq\_j}}\right]\right)\hat{R}_{AB}\hat{R}_{AB} \\ -2\left(\frac{d_{AB}^{eq\_j}}{d_{AB}^{\ 2}}+\frac{1}{d_{AB}^{eq\_j}}\right)^2\tanh\left[\frac{d_{AB}^{eq\_j}}{d_{AB}}-\frac{d_{AB}}{d_{AB}^{eq\_j}}\right]\left(1-\tanh^2\left[\frac{d_{AB}^{eq\_j}}{d_{AB}}-\frac{d_{AB}}{d_{AB}^{eq\_j}}\right]\right)\hat{R}_{AB}\hat{R}_{AB} \\ -\frac{1}{d_{AB}}\left(\frac{d_{AB}^{eq\_j}}{d_{AB}^{\ 2}}+\frac{1}{d_{AB}^{eq\_j}}\right)\left(1-\tanh^2\left[\frac{d_{AB}^{eq\_j}}{d_{AB}}-\frac{d_{AB}}{d_{AB}^{eq\_j}}\right]\right)\vec{\vec{\delta}} \end{pmatrix} \tag{S16}$$

$$\vec{\nabla}_A\vec{\nabla}_B\tau_{AB}\left[d_{AB},d_{AB}^{eq\_j}\right]=\vec{\nabla}_B\vec{\nabla}_A\tau_{AB}\left[d_{AB},d_{AB}^{eq\_j}\right]=-\vec{\nabla}_A\vec{\nabla}_A\tau_{AB}\left[d_{AB},d_{AB}^{eq\_j}\right] \tag{S17}$$

In the below formulas, atom_1 is any chosen atom in the material, and atom_2 is any chosen atom in the material. *Note*: atom_2 may either be the same atom or a different atom than atom_1.

Case # 1: The two atoms A and B are inside the same bonded cluster j and a cutoff distance is used for their nonbonded interaction. In this case, we express the effective multibody pairwise potentials as follows:

$$\Phi_{ABx}^{intercluster}=\Theta_H\left[d_{cutoff}^{nonbonded}-d_{AB}\right]\tau_{AB}^{\ 3}\left[d_{AB},d_{cutoff}^{nonbonded}\right]\tau_{AB}^{\ 2}\left[d_{AB},d_{AB}^{eq\_j}\right]\left(U_{ABx,intercluster}^{nonbonded}\left[\left\{\vec{R}_C\right\}\right]-U_{ABx,intercluster}^{nonbonded}\left[\left\{\vec{R}_C^{eq-j}\right\}\right]\right) \tag{S18}$$

$$\Phi_{ABx}^{intracluster}=\Theta_H\left[d_{cutoff}^{nonbonded}-d_{AB}\right]\tau_{AB}^{\ 3}\left[d_{AB},d_{cutoff}^{nonbonded}\right]\tau_{AB}^{\ 2}\left[d_{AB},d_{AB}^{eq\_j}\right]\left(U_{ABx,intracluster}^{nonbonded}\left[\left\{\vec{R}_C\right\}\right]-U_{ABx,intracluster}^{nonbonded}\left[\left\{\vec{R}_C^{eq-j}\right\}\right]\right) \tag{S19}$$

Θ_H is the Heaviside step function, and $d_{AB}^{eq\_j}$ is the equilibrium distance between atoms A and B in the isolated bonded cluster j. The first-order and second-order derivatives expand as follows:

$$\vec{\nabla}_{atom\_1}\Phi_{ABx}^{intercluster}=\Theta_H\left[d_{cutoff}^{nonbonded}-d_{AB}\right]$$
$$\begin{pmatrix} 3\tau_{AB}^{\ 2}\left[d_{AB},d_{cutoff}^{nonbonded}\right]\tau_{AB}^{\ 2}\left[d_{AB},d_{AB}^{eq\_j}\right]\left(U_{ABx,intercluster}^{nonbonded}\left[\left\{\vec{R}_C\right\}\right]-U_{ABx,intercluster}^{nonbonded}\left[\left\{\vec{R}_C^{eq-j}\right\}\right]\right)\vec{\nabla}_{atom\_1}\tau_{AB}\left[d_{AB},d_{cutoff}^{nonbonded}\right] \\ +2\tau_{AB}^{\ 3}\left[d_{AB},d_{cutoff}^{nonbonded}\right]\tau_{AB}\left[d_{AB},d_{AB}^{eq\_j}\right]\left(U_{ABx,intercluster}^{nonbonded}\left[\left\{\vec{R}_C\right\}\right]-U_{ABx,intercluster}^{nonbonded}\left[\left\{\vec{R}_C^{eq-j}\right\}\right]\right)\vec{\nabla}_{atom\_1}\tau_{AB}\left[d_{AB},d_{AB}^{eq\_j}\right] \\ +\tau_{AB}^{\ 3}\left[d_{AB},d_{cutoff}^{nonbonded}\right]\tau_{AB}^{\ 2}\left[d_{AB},d_{AB}^{eq\_j}\right]\vec{\nabla}_{atom\_1}U_{ABx,intercluster}^{nonbonded}\left[\left\{\vec{R}_C\right\}\right] \end{pmatrix} \tag{S20}$$

$$\vec{\nabla}_{atom\_1}\Phi_{ABx}^{intracluster}=\Theta_H\left[d_{cutoff}^{nonbonded}-d_{AB}\right]$$
$$\begin{pmatrix} 3\tau_{AB}^{\ 2}\left[d_{AB},d_{cutoff}^{nonbonded}\right]\tau_{AB}^{\ 2}\left[d_{AB},d_{AB}^{eq\_j}\right]\left(U_{ABx,intracluster}^{nonbonded}\left[\left\{\vec{R}_C\right\}\right]-U_{ABx,intracluster}^{nonbonded}\left[\left\{\vec{R}_C^{eq-j}\right\}\right]\right)\vec{\nabla}_{atom\_1}\tau_{AB}\left[d_{AB},d_{cutoff}^{nonbonded}\right] \\ +2\tau_{AB}^{\ 3}\left[d_{AB},d_{cutoff}^{nonbonded}\right]\tau_{AB}\left[d_{AB},d_{AB}^{eq\_j}\right]\left(U_{ABx,intracluster}^{nonbonded}\left[\left\{\vec{R}_C\right\}\right]-U_{ABx,intracluster}^{nonbonded}\left[\left\{\vec{R}_C^{eq-j}\right\}\right]\right)\vec{\nabla}_{atom\_1}\tau_{AB}\left[d_{AB},d_{AB}^{eq\_j}\right] \\ +\tau_{AB}^{\ 3}\left[d_{AB},d_{cutoff}^{nonbonded}\right]\tau_{AB}^{\ 2}\left[d_{AB},d_{AB}^{eq\_j}\right]\vec{\nabla}_{atom\_1}U_{ABx,intracluster}^{nonbonded}\left[\left\{\vec{R}_C\right\}\right] \end{pmatrix} \tag{S21}$$

$$\vec{\nabla}_{atom\_1}\vec{\nabla}_{atom\_2}\Phi_{ABx}^{intercluster}=\Theta_H\left[d_{cutoff}^{nonbonded}-d_{AB}\right]$$
$$\left(\begin{array}{l}
3\tau_{AB}{}^{2}\left[d_{AB},d_{cutoff}^{nonbonded}\right]\tau_{AB}{}^{2}\left[d_{AB},d_{AB}^{eq_j}\right]\left(U_{ABx,intercluster}^{nonbonded}\left[\left\{\vec{R}_C\right\}\right]-U_{ABx,intercluster}^{nonbonded}\left[\left\{\vec{R}_C^{eq_j}\right\}\right]\right)\vec{\nabla}_{atom_1}\vec{\nabla}_{atom_2}\tau_{AB}\left[d_{AB},d_{cutoff}^{nonbonded}\right]\\
+2\tau_{AB}{}^{3}\left[d_{AB},d_{cutoff}^{nonbonded}\right]\tau_{AB}\left[d_{AB},d_{AB}^{eq_j}\right]\left(U_{ABx,intercluster}^{nonbonded}\left[\left\{\vec{R}_C\right\}\right]-U_{ABx,intercluster}^{nonbonded}\left[\left\{\vec{R}_C^{eq_j}\right\}\right]\right)\vec{\nabla}_{atom_1}\vec{\nabla}_{atom_2}\tau_{AB}\left[d_{AB},d_{AB}^{eq_j}\right]\\
+\tau_{AB}{}^{3}\left[d_{AB},d_{cutoff}^{nonbonded}\right]\tau_{AB}{}^{2}\left[d_{AB},d_{AB}^{eq_j}\right]\vec{\nabla}_{atom_1}\vec{\nabla}_{atom_2}U_{ABx,intercluster}^{nonbonded}\left[\left\{\vec{R}_C\right\}\right]\\
+3\tau_{AB}{}^{2}\left[d_{AB},d_{cutoff}^{nonbonded}\right]\tau_{AB}{}^{2}\left[d_{AB},d_{AB}^{eq_j}\right]\left(\vec{\nabla}_{atom_1}U_{ABx,intercluster}^{nonbonded}\left[\left\{\vec{R}_C\right\}\right]\right)\vec{\nabla}_{atom_2}\tau_{AB}\left[d_{AB},d_{cutoff}^{nonbonded}\right]\\
+3\tau_{AB}{}^{2}\left[d_{AB},d_{cutoff}^{nonbonded}\right]\tau_{AB}{}^{2}\left[d_{AB},d_{AB}^{eq_j}\right]\left(\vec{\nabla}_{atom_1}\tau_{AB}\left[d_{AB},d_{cutoff}^{nonbonded}\right]\right)\vec{\nabla}_{atom_2}U_{ABx,intercluster}^{nonbonded}\left[\left\{\vec{R}_C\right\}\right]\\
+2\tau_{AB}{}^{3}\left[d_{AB},d_{cutoff}^{nonbonded}\right]\tau_{AB}\left[d_{AB},d_{AB}^{eq_j}\right]\left(\vec{\nabla}_{atom_1}U_{ABx,intercluster}^{nonbonded}\left[\left\{\vec{R}_C\right\}\right]\right)\vec{\nabla}_{atom_2}\tau_{AB}\left[d_{AB},d_{AB}^{eq_j}\right]\\
+2\tau_{AB}{}^{3}\left[d_{AB},d_{cutoff}^{nonbonded}\right]\tau_{AB}\left[d_{AB},d_{AB}^{eq_j}\right]\left(\vec{\nabla}_{atom_1}\tau_{AB}\left[d_{AB},d_{AB}^{eq_j}\right]\right)\vec{\nabla}_{atom_2}U_{ABx,intercluster}^{nonbonded}\left[\left\{\vec{R}_C\right\}\right]\\
+6\tau_{AB}{}^{2}\left[d_{AB},d_{cutoff}^{nonbonded}\right]\tau_{AB}\left[d_{AB},d_{AB}^{eq_j}\right]\left(U_{ABx,intercluster}^{nonbonded}\left[\left\{\vec{R}_C\right\}\right]-U_{ABx,intercluster}^{nonbonded}\left[\left\{\vec{R}_C^{eq_j}\right\}\right]\right)\left(\vec{\nabla}_{atom_1}\tau_{AB}\left[d_{AB},d_{AB}^{eq_j}\right]\right)\vec{\nabla}_{atom_2}\tau_{AB}\left[d_{AB},d_{cutoff}^{nonbonded}\right]\\
+6\tau_{AB}{}^{2}\left[d_{AB},d_{cutoff}^{nonbonded}\right]\tau_{AB}\left[d_{AB},d_{AB}^{eq_j}\right]\left(U_{ABx,intercluster}^{nonbonded}\left[\left\{\vec{R}_C\right\}\right]-U_{ABx,intercluster}^{nonbonded}\left[\left\{\vec{R}_C^{eq_j}\right\}\right]\right)\left(\vec{\nabla}_{atom_1}\tau_{AB}\left[d_{AB},d_{cutoff}^{nonbonded}\right]\right)\vec{\nabla}_{atom_2}\tau_{AB}\left[d_{AB},d_{AB}^{eq_j}\right]\\
+6\tau_{AB}\left[d_{AB},d_{cutoff}^{nonbonded}\right]\tau_{AB}{}^{2}\left[d_{AB},d_{AB}^{eq_j}\right]\left(U_{ABx,intercluster}^{nonbonded}\left[\left\{\vec{R}_C\right\}\right]-U_{ABx,intercluster}^{nonbonded}\left[\left\{\vec{R}_C^{eq_j}\right\}\right]\right)\left(\vec{\nabla}_{atom_1}\tau_{AB}\left[d_{AB},d_{cutoff}^{nonbonded}\right]\right)\vec{\nabla}_{atom_2}\tau_{AB}\left[d_{AB},d_{cutoff}^{nonbonded}\right]\\
+2\tau_{AB}{}^{3}\left[d_{AB},d_{cutoff}^{nonbonded}\right]\left(U_{ABx,intercluster}^{nonbonded}\left[\left\{\vec{R}_C\right\}\right]-U_{ABx,intercluster}^{nonbonded}\left[\left\{\vec{R}_C^{eq_j}\right\}\right]\right)\left(\vec{\nabla}_{atom_1}\tau_{AB}\left[d_{AB},d_{AB}^{eq_j}\right]\right)\vec{\nabla}_{atom_2}\tau_{AB}\left[d_{AB},d_{AB}^{eq_j}\right]
\end{array}\right) \quad \text{(S22)}$$

$$\vec{\nabla}_{atom\_1}\vec{\nabla}_{atom\_2}\Phi_{ABx}^{intracluster}=\Theta_H\left[d_{cutoff}^{nonbonded}-d_{AB}\right]$$
$$\left(\begin{array}{l}
3\tau_{AB}{}^{2}\left[d_{AB},d_{cutoff}^{nonbonded}\right]\tau_{AB}{}^{2}\left[d_{AB},d_{AB}^{eq_j}\right]\left(U_{ABx,intracluster}^{nonbonded}\left[\left\{\vec{R}_C\right\}\right]-U_{ABx,intracluster}^{nonbonded}\left[\left\{\vec{R}_C^{eq_j}\right\}\right]\right)\vec{\nabla}_{atom_1}\vec{\nabla}_{atom_2}\tau_{AB}\left[d_{AB},d_{cutoff}^{nonbonded}\right]\\
+2\tau_{AB}{}^{3}\left[d_{AB},d_{cutoff}^{nonbonded}\right]\tau_{AB}\left[d_{AB},d_{AB}^{eq_j}\right]\left(U_{ABx,intracluster}^{nonbonded}\left[\left\{\vec{R}_C\right\}\right]-U_{ABx,intracluster}^{nonbonded}\left[\left\{\vec{R}_C^{eq_j}\right\}\right]\right)\vec{\nabla}_{atom_1}\vec{\nabla}_{atom_2}\tau_{AB}\left[d_{AB},d_{AB}^{eq_j}\right]\\
+\tau_{AB}{}^{3}\left[d_{AB},d_{cutoff}^{nonbonded}\right]\tau_{AB}{}^{2}\left[d_{AB},d_{AB}^{eq_j}\right]\vec{\nabla}_{atom_1}\vec{\nabla}_{atom_2}U_{ABx,intracluster}^{nonbonded}\left[\left\{\vec{R}_C\right\}\right]\\
+3\tau_{AB}{}^{2}\left[d_{AB},d_{cutoff}^{nonbonded}\right]\tau_{AB}{}^{2}\left[d_{AB},d_{AB}^{eq_j}\right]\left(\vec{\nabla}_{atom_1}U_{ABx,intracluster}^{nonbonded}\left[\left\{\vec{R}_C\right\}\right]\right)\vec{\nabla}_{atom_2}\tau_{AB}\left[d_{AB},d_{cutoff}^{nonbonded}\right]\\
+3\tau_{AB}{}^{2}\left[d_{AB},d_{cutoff}^{nonbonded}\right]\tau_{AB}{}^{2}\left[d_{AB},d_{AB}^{eq_j}\right]\left(\vec{\nabla}_{atom_1}\tau_{AB}\left[d_{AB},d_{cutoff}^{nonbonded}\right]\right)\vec{\nabla}_{atom_2}U_{ABx,intracluster}^{nonbonded}\left[\left\{\vec{R}_C\right\}\right]\\
+2\tau_{AB}{}^{3}\left[d_{AB},d_{cutoff}^{nonbonded}\right]\tau_{AB}\left[d_{AB},d_{AB}^{eq_j}\right]\left(\vec{\nabla}_{atom_1}U_{ABx,intracluster}^{nonbonded}\left[\left\{\vec{R}_C\right\}\right]\right)\vec{\nabla}_{atom_2}\tau_{AB}\left[d_{AB},d_{AB}^{eq_j}\right]\\
+2\tau_{AB}{}^{3}\left[d_{AB},d_{cutoff}^{nonbonded}\right]\tau_{AB}\left[d_{AB},d_{AB}^{eq_j}\right]\left(\vec{\nabla}_{atom_1}\tau_{AB}\left[d_{AB},d_{AB}^{eq_j}\right]\right)\vec{\nabla}_{atom_2}U_{ABx,intracluster}^{nonbonded}\left[\left\{\vec{R}_C\right\}\right]\\
+6\tau_{AB}{}^{2}\left[d_{AB},d_{cutoff}^{nonbonded}\right]\tau_{AB}\left[d_{AB},d_{AB}^{eq_j}\right]\left(U_{ABx,intracluster}^{nonbonded}\left[\left\{\vec{R}_C\right\}\right]-U_{ABx,intracluster}^{nonbonded}\left[\left\{\vec{R}_C^{eq_j}\right\}\right]\right)\left(\vec{\nabla}_{atom_1}\tau_{AB}\left[d_{AB},d_{AB}^{eq_j}\right]\right)\vec{\nabla}_{atom_2}\tau_{AB}\left[d_{AB},d_{cutoff}^{nonbonded}\right]\\
+6\tau_{AB}{}^{2}\left[d_{AB},d_{cutoff}^{nonbonded}\right]\tau_{AB}\left[d_{AB},d_{AB}^{eq_j}\right]\left(U_{ABx,intracluster}^{nonbonded}\left[\left\{\vec{R}_C\right\}\right]-U_{ABx,intracluster}^{nonbonded}\left[\left\{\vec{R}_C^{eq_j}\right\}\right]\right)\left(\vec{\nabla}_{atom_1}\tau_{AB}\left[d_{AB},d_{cutoff}^{nonbonded}\right]\right)\vec{\nabla}_{atom_2}\tau_{AB}\left[d_{AB},d_{AB}^{eq_j}\right]\\
+6\tau_{AB}\left[d_{AB},d_{cutoff}^{nonbonded}\right]\tau_{AB}{}^{2}\left[d_{AB},d_{AB}^{eq_j}\right]\left(U_{ABx,intracluster}^{nonbonded}\left[\left\{\vec{R}_C\right\}\right]-U_{ABx,intracluster}^{nonbonded}\left[\left\{\vec{R}_C^{eq_j}\right\}\right]\right)\left(\vec{\nabla}_{atom_1}\tau_{AB}\left[d_{AB},d_{cutoff}^{nonbonded}\right]\right)\vec{\nabla}_{atom_2}\tau_{AB}\left[d_{AB},d_{cutoff}^{nonbonded}\right]\\
+2\tau_{AB}{}^{3}\left[d_{AB},d_{cutoff}^{nonbonded}\right]\left(U_{ABx,intracluster}^{nonbonded}\left[\left\{\vec{R}_C\right\}\right]-U_{ABx,intracluster}^{nonbonded}\left[\left\{\vec{R}_C^{eq_j}\right\}\right]\right)\left(\vec{\nabla}_{atom_1}\tau_{AB}\left[d_{AB},d_{AB}^{eq_j}\right]\right)\vec{\nabla}_{atom_2}\tau_{AB}\left[d_{AB},d_{AB}^{eq_j}\right]
\end{array}\right) \quad \text{(S23)}$$

Case # 2: The two atoms A and B are inside the same bonded cluster j and a cutoff distance is not used for their nonbonded interaction. In this case, we express the effective multibody pairwise potentials as follows:

$$\Phi_{ABx}^{intercluster} = \tau_{AB}{}^{2}\left[d_{AB}, d_{AB}^{eq\_j}\right]\left(U_{ABx,intercluster}^{nonbonded}\left[\left\{\vec{R}_C\right\}\right] - U_{ABx,intercluster}^{nonbonded}\left[\left\{\vec{R}_C^{eq-j}\right\}\right]\right) \quad \text{(S24)}$$

$$\Phi_{ABx}^{intracluster} = \tau_{AB}{}^{2}\left[d_{AB}, d_{AB}^{eq\_j}\right]\left(U_{ABx,intracluster}^{nonbonded}\left[\left\{\vec{R}_C\right\}\right] - U_{ABx,intracluster}^{nonbonded}\left[\left\{\vec{R}_C^{eq-j}\right\}\right]\right) \quad \text{(S25)}$$

The first-order and second-order derivatives expand as follows:

$$\vec{\nabla}_{atom\_1}\Phi_{ABx}^{intercluster} = \Theta_H\left[d_{cutoff}^{nonbonded} - d_{AB}\right] \begin{pmatrix} 2\tau_{AB}\left[d_{AB}, d_{AB}^{eq\_j}\right]\left(U_{ABx,intercluster}^{nonbonded}\left[\left\{\vec{R}_C\right\}\right] - U_{ABx,intercluster}^{nonbonded}\left[\left\{\vec{R}_C^{eq-j}\right\}\right]\right)\vec{\nabla}_{atom\_1}\tau_{AB}\left[d_{AB}, d_{AB}^{eq\_j}\right] \\ +\tau_{AB}{}^{2}\left[d_{AB}, d_{AB}^{eq\_j}\right]\vec{\nabla}_{atom\_1}U_{ABx,intercluster}^{nonbonded}\left[\left\{\vec{R}_C\right\}\right] \end{pmatrix} \quad \text{(S26)}$$

$$\vec{\nabla}_{atom\_1}\Phi_{ABx}^{intracluster} = \Theta_H\left[d_{cutoff}^{nonbonded} - d_{AB}\right] \begin{pmatrix} 2\tau_{AB}\left[d_{AB}, d_{AB}^{eq\_j}\right]\left(U_{ABx,intracluster}^{nonbonded}\left[\left\{\vec{R}_C\right\}\right] - U_{ABx,intracluster}^{nonbonded}\left[\left\{\vec{R}_C^{eq-j}\right\}\right]\right)\vec{\nabla}_{atom\_1}\tau_{AB}\left[d_{AB}, d_{AB}^{eq\_j}\right] \\ +\tau_{AB}{}^{2}\left[d_{AB}, d_{AB}^{eq\_j}\right]\vec{\nabla}_{atom\_1}U_{ABx,intracluster}^{nonbonded}\left[\left\{\vec{R}_C\right\}\right] \end{pmatrix} \quad \text{(S27)}$$

$$\vec{\nabla}_{atom\_1}\vec{\nabla}_{atom\_2}\Phi_{ABx}^{intercluster} = \Theta_H\left[d_{cutoff}^{nonbonded} - d_{AB}\right] \begin{pmatrix} 2\tau_{AB}\left[d_{AB}, d_{AB}^{eq\_j}\right]\left(U_{ABx,intercluster}^{nonbonded}\left[\left\{\vec{R}_C\right\}\right] - U_{ABx,intercluster}^{nonbonded}\left[\left\{\vec{R}_C^{eq-j}\right\}\right]\right)\vec{\nabla}_{atom\_1}\vec{\nabla}_{atom\_2}\tau_{AB}\left[d_{AB}, d_{AB}^{eq\_j}\right] \\ +\tau_{AB}{}^{2}\left[d_{AB}, d_{AB}^{eq\_j}\right]\vec{\nabla}_{atom\_1}\vec{\nabla}_{atom\_2}U_{ABx,intercluster}^{nonbonded}\left[\left\{\vec{R}_C\right\}\right] \\ +2\tau_{AB}\left[d_{AB}, d_{AB}^{eq\_j}\right]\left(\vec{\nabla}_{atom\_1}U_{ABx,intercluster}^{nonbonded}\left[\left\{\vec{R}_C\right\}\right]\right)\vec{\nabla}_{atom\_2}\tau_{AB}\left[d_{AB}, d_{AB}^{eq\_j}\right] \\ +2\tau_{AB}\left[d_{AB}, d_{AB}^{eq\_j}\right]\left(\vec{\nabla}_{atom\_1}\tau_{AB}\left[d_{AB}, d_{AB}^{eq\_j}\right]\right)\vec{\nabla}_{atom\_2}U_{ABx,intercluster}^{nonbonded}\left[\left\{\vec{R}_C\right\}\right] \\ +2\left(U_{ABx,intercluster}^{nonbonded}\left[\left\{\vec{R}_C\right\}\right] - U_{ABx,intercluster}^{nonbonded}\left[\left\{\vec{R}_C^{eq-j}\right\}\right]\right)\left(\vec{\nabla}_{atom\_1}\tau_{AB}\left[d_{AB}, d_{AB}^{eq\_j}\right]\right)\vec{\nabla}_{atom\_2}\tau_{AB}\left[d_{AB}, d_{AB}^{eq\_j}\right] \end{pmatrix} \quad \text{(S28)}$$

$$\vec{\nabla}_{atom\_1}\vec{\nabla}_{atom\_2}\Phi_{ABx}^{intracluster} = \Theta_H\left[d_{cutoff}^{nonbonded} - d_{AB}\right] \begin{pmatrix} 2\tau_{AB}\left[d_{AB}, d_{AB}^{eq\_j}\right]\left(U_{ABx,intracluster}^{nonbonded}\left[\left\{\vec{R}_C\right\}\right] - U_{ABx,intracluster}^{nonbonded}\left[\left\{\vec{R}_C^{eq-j}\right\}\right]\right)\vec{\nabla}_{atom\_1}\vec{\nabla}_{atom\_2}\tau_{AB}\left[d_{AB}, d_{AB}^{eq\_j}\right] \\ +\tau_{AB}{}^{2}\left[d_{AB}, d_{AB}^{eq\_j}\right]\vec{\nabla}_{atom\_1}\vec{\nabla}_{atom\_2}U_{ABx,intracluster}^{nonbonded}\left[\left\{\vec{R}_C\right\}\right] \\ +2\tau_{AB}\left[d_{AB}, d_{AB}^{eq\_j}\right]\left(\vec{\nabla}_{atom\_1}U_{ABx,intracluster}^{nonbonded}\left[\left\{\vec{R}_C\right\}\right]\right)\vec{\nabla}_{atom\_2}\tau_{AB}\left[d_{AB}, d_{AB}^{eq\_j}\right] \\ +2\tau_{AB}\left[d_{AB}, d_{AB}^{eq\_j}\right]\left(\vec{\nabla}_{atom\_1}\tau_{AB}\left[d_{AB}, d_{AB}^{eq\_j}\right]\right)\vec{\nabla}_{atom\_2}U_{ABx,intracluster}^{nonbonded}\left[\left\{\vec{R}_C\right\}\right] \\ +2\left(U_{ABx,intracluster}^{nonbonded}\left[\left\{\vec{R}_C\right\}\right] - U_{ABx,intracluster}^{nonbonded}\left[\left\{\vec{R}_C^{eq-j}\right\}\right]\right)\left(\vec{\nabla}_{atom\_1}\tau_{AB}\left[d_{AB}, d_{AB}^{eq\_j}\right]\right)\vec{\nabla}_{atom\_2}\tau_{AB}\left[d_{AB}, d_{AB}^{eq\_j}\right] \end{pmatrix} \quad \text{(S29)}$$

Case # 3: The two atoms A and D are not inside the same bonded cluster and a cutoff distance is used for their nonbonded interaction. In this case, we express the effective multibody pairwise potentials as follows:

$$\Phi_{ADx}^{intercluster} = \Theta_H\left[d_{cutoff}^{nonbonded} - d_{AB}\right]\tau_{AB}{}^{3}\left[d_{AB}, d_{cutoff}^{nonbonded}\right]U_{ABx,intercluster}^{nonbonded}\left[\left\{\vec{R}_C\right\}\right] \quad (S30)$$

$$\Phi_{ADx}^{intracluster} = 0 \quad (S31)$$

The first-order and second-order derivatives expand as follows:

$$\vec{\nabla}_{atom\_1}\Phi_{ABx}^{intercluster} = \Theta_H\left[d_{cutoff}^{nonbonded} - d_{AB}\right] \begin{pmatrix} 3\tau_{AB}{}^{2}\left[d_{AB}, d_{cutoff}^{nonbonded}\right]\left(U_{ABx,intercluster}^{nonbonded}\left[\left\{\vec{R}_C\right\}\right] - U_{ABx,intercluster}^{nonbonded}\left[\left\{\vec{R}_C^{eq\_j}\right\}\right]\right)\vec{\nabla}_{atom\_1}\tau_{AB}\left[d_{AB}, d_{cutoff}^{nonbonded}\right] \\ +\tau_{AB}{}^{3}\left[d_{AB}, d_{cutoff}^{nonbonded}\right]\vec{\nabla}_{atom\_1}U_{ABx,intercluster}^{nonbonded}\left[\left\{\vec{R}_C\right\}\right] \end{pmatrix} \quad (S32)$$

$$\vec{\nabla}_{atom\_1}\Phi_{ABx}^{intracluster} = 0 \quad (S33)$$

$$\vec{\nabla}_{atom\_1}\vec{\nabla}_{atom\_2}\Phi_{ABx}^{intercluster} = \Theta_H\left[d_{cutoff}^{nonbonded} - d_{AB}\right] \begin{pmatrix} 3\tau_{AB}{}^{2}\left[d_{AB}, d_{cutoff}^{nonbonded}\right]\left(U_{ABx,intercluster}^{nonbonded}\left[\left\{\vec{R}_C\right\}\right] - U_{ABx,intercluster}^{nonbonded}\left[\left\{\vec{R}_C^{eq\_j}\right\}\right]\right)\vec{\nabla}_{atom\_1}\vec{\nabla}_{atom\_2}\tau_{AB}\left[d_{AB}, d_{cutoff}^{nonbonded}\right] \\ +\tau_{AB}{}^{3}\left[d_{AB}, d_{cutoff}^{nonbonded}\right]\vec{\nabla}_{atom\_1}\vec{\nabla}_{atom\_2}U_{ABx,intercluster}^{nonbonded}\left[\left\{\vec{R}_C\right\}\right] \\ +3\tau_{AB}{}^{2}\left[d_{AB}, d_{cutoff}^{nonbonded}\right]\left(\vec{\nabla}_{atom\_1}U_{ABx,intercluster}^{nonbonded}\left[\left\{\vec{R}_C\right\}\right]\right)\vec{\nabla}_{atom\_2}\tau_{AB}\left[d_{AB}, d_{cutoff}^{nonbonded}\right] \\ +3\tau_{AB}{}^{2}\left[d_{AB}, d_{cutoff}^{nonbonded}\right]\left(\vec{\nabla}_{atom\_1}\tau_{AB}\left[d_{AB}, d_{cutoff}^{nonbonded}\right]\right)\vec{\nabla}_{atom\_2}U_{ABx,intercluster}^{nonbonded}\left[\left\{\vec{R}_C\right\}\right] \\ +6\tau_{AB}\left[d_{AB}, d_{cutoff}^{nonbonded}\right]\left(U_{ABx,intercluster}^{nonbonded}\left[\left\{\vec{R}_C\right\}\right] - U_{ABx,intercluster}^{nonbonded}\left[\left\{\vec{R}_C^{eq\_j}\right\}\right]\right)\left(\vec{\nabla}_{atom\_1}\tau_{AB}\left[d_{AB}, d_{cutoff}^{nonbonded}\right]\right)\vec{\nabla}_{atom\_2}\tau_{AB}\left[d_{AB}, d_{cutoff}^{nonbonded}\right] \end{pmatrix} \quad (S34)$$

$$\vec{\nabla}_{atom\_1}\vec{\nabla}_{atom\_2}\Phi_{ADx}^{intracluster} = 0 \quad (S35)$$

Case # 4: The two atoms A and D are not inside the same bonded cluster and a cutoff distance is not used for their nonbonded interaction. In this case, we express the effective multibody pairwise potentials as follows:

$$\Phi_{ADx}^{intercluster} = U_{ABx,intercluster}^{nonbonded}\left[\left\{\vec{R}_C\right\}\right] \quad (S36)$$

$$\Phi_{ADx}^{intracluster} = 0 \quad (S37)$$

The first-order and second-order derivatives expand as follows:

$$\vec{\nabla}_{atom\_1}\Phi_{ABx}^{intercluster} = \vec{\nabla}_{atom\_1}U_{ABx,intercluster}^{nonbonded}\left[\left\{\vec{R}_C\right\}\right] \quad (S38)$$

$$\vec{\nabla}_{atom\_1}\Phi_{ADx}^{intracluster} = 0 \quad (S39)$$

$$\vec{\nabla}_{atom\_1}\vec{\nabla}_{atom\_2}\Phi_{ABx}^{intercluster} = \vec{\nabla}_{atom\_1}\vec{\nabla}_{atom\_2}U_{ABx,intercluster}^{nonbonded}\left[\left\{\vec{R}_C\right\}\right] \quad (S40)$$

$$\vec{\nabla}_{atom\_1}\vec{\nabla}_{atom\_2}\Phi_{ADx}^{intracluster} = 0 \quad (S41)$$

S3. Analytic derivatives of the Manz stretch potential

$$U_{AB}^{Manz\_stretch}\left[d_{AB}\right]=\frac{3k_{AB}}{5\mathring{\gamma}_{AB}^{\ 2}}\left(1-\left(\frac{5}{2}\right)\exp\left[-\mathring{\gamma}_{AB}\left(d_{AB}-d_{AB}^{ref}\right)\right]+\left(\frac{3}{2}\right)\exp\left[-\frac{5}{3}\mathring{\gamma}_{AB}\left(d_{AB}-d_{AB}^{ref}\right)\right]\right) \quad (S42)$$

$$\frac{dU_{AB}^{Manz\_stretch}\left[d_{AB}\right]}{dd_{AB}}=\frac{3k_{AB}}{2\mathring{\gamma}_{AB}}\left(\exp\left[-\mathring{\gamma}_{AB}\left(d_{AB}-d_{AB}^{ref}\right)\right]-\exp\left[-\frac{5}{3}\mathring{\gamma}_{AB}\left(d_{AB}-d_{AB}^{ref}\right)\right]\right) \quad (S43)$$

$$\frac{d^2U_{AB}^{Manz\_stretch}\left[d_{AB}\right]}{dd_{AB}^{\ 2}}=k_{AB}\left(-\frac{3}{2}\exp\left[-\mathring{\gamma}_{AB}\left(d_{AB}-d_{AB}^{ref}\right)\right]+\frac{5}{2}\exp\left[-\frac{5}{3}\mathring{\gamma}_{AB}\left(d_{AB}-d_{AB}^{ref}\right)\right]\right) \quad (S44)$$

$$\frac{d^3U_{AB}^{Manz\_stretch}\left[d_{AB}\right]}{dd_{AB}^{\ 3}}=k_{AB}\mathring{\gamma}_{AB}\left(\frac{3}{2}\exp\left[-\mathring{\gamma}_{AB}\left(d_{AB}-d_{AB}^{ref}\right)\right]-\frac{25}{6}\exp\left[-\frac{5}{3}\mathring{\gamma}_{AB}\left(d_{AB}-d_{AB}^{ref}\right)\right]\right) \quad (S45)$$

$$\frac{d^4U_{AB}^{Manz\_stretch}\left[d_{AB}\right]}{dd_{AB}^{\ 4}}=k_{AB}\mathring{\gamma}_{AB}^{\ 2}\left(-\frac{3}{2}\exp\left[-\mathring{\gamma}_{AB}\left(d_{AB}-d_{AB}^{ref}\right)\right]+\frac{125}{18}\exp\left[-\frac{5}{3}\mathring{\gamma}_{AB}\left(d_{AB}-d_{AB}^{ref}\right)\right]\right) \quad (S46)$$

S4. Analytic derivatives of my new angle-bending potential

Consider a bond angle defined by the atoms A, B, and C, where B is the middle atom. Swope and Ferguson[S1] and Dubbeldam et al.[S2] gave universal formulas for first and second derivatives of any angle-bending potential $U_{\measuredangle}[\theta]$ with respect to changes in the Cartesian coordinates of atoms A, B, C. Dubbeldam et al.[S2] also gave universal formulas for first and second derivatives of any angle-bending potential with respect to changes in the unit cell's size and shape for unrelaxed homogeneous strain. Their formulas require the following inputs:

$$f_1=\frac{dU_{\measuredangle}[\theta]}{d\cos[\theta]} \quad (S47)$$

$$f_2=\frac{d^2U_{\measuredangle}[\theta]}{\left(d\cos[\theta]\right)^2} \quad (S48)$$

For conciseness, we first define

$$U_{new}[\theta]=k\frac{2\left(\cos\theta-\cos\theta_{eq}\right)^2}{\sin^2\theta+3\sin^2\theta_{eq}\left(\frac{\tanh\left[2\sin[\theta/2]\right]}{\tanh\left[2\sin\left[\theta_{eq}/2\right]\right]}\right)}=k\frac{2\left(\cos\theta-\cos\theta_{eq}\right)^2}{w[\theta]} \quad (S49)$$

$$p[\theta]=\sqrt{2(1-\cos\theta)}=2\sin[\theta/2] \quad (S50)$$

$$tp[\theta]=\tanh\left[p[\theta]\right] \quad (S51)$$

$$w[\theta]=1-\cos^2\theta+3\left(1-\cos^2\theta_{eq}\right)\left(\frac{tp[\theta]}{tp\left[\theta_{eq}\right]}\right) \quad (S52)$$

Its derivatives are

$$\mu[\theta]=-3\left(\frac{1-\cos^2\theta_{eq}}{p[\theta]}\right)\left(\frac{1-\left(tp[\theta]\right)^2}{tp\left[\theta_{eq}\right]}\right) \quad (S53)$$

$$\frac{dw[\theta]}{d\cos[\theta]} = -2\cos\theta + \mu[\theta] \tag{S54}$$

$$\frac{d^2w[\theta]}{(d\cos[\theta])^2} = -2 + \frac{\mu[\theta]}{p[\theta]}\left(2tp[\theta] + \frac{1}{(p[\theta])^2}\right) \tag{S55}$$

The derivatives of my new angle-bending function are:

$$\frac{dU_{new}[\theta]}{d\cos[\theta]} = \frac{1}{w[\theta,\theta_{eq}]}\left(4k(\cos\theta - \cos\theta_{eq}) - U_{new}[\theta]\frac{dw[\theta]}{d\cos[\theta]}\right) \tag{S56}$$

$$\frac{d^2U_{new}[\theta]}{(d\cos[\theta])^2} = \frac{1}{w[\theta,\theta_{eq}]}\left(4k - 2\frac{dU_{new}[\theta]}{d\cos[\theta]}\frac{dw[\theta]}{d\cos[\theta]} - U_{new}[\theta]\frac{d^2w[\theta]}{(d\cos[\theta])^2}\right) \tag{S57}$$

Eqn (S54)–(S57) were derived by substituting t = cos[θ] into eqn (S49) or (S52) and then differentiating with respect to t. Eqn (S56) and (S57) can be used to compute the potential's derivatives with respect to bond angle changes:

$$\frac{dU_{new}[\theta]}{d\theta} = -\sin[\theta]\frac{dU_{new}[\theta]}{d\cos[\theta]} \tag{S58}$$

$$\frac{d^2U_{new}[\theta]}{d\theta^2} = -\cos[\theta]\frac{dU_{new}[\theta]}{d\cos[\theta]} + \sin^2[\theta]\frac{d^2U_{new}[\theta]}{(d\cos[\theta])^2} \tag{S59}$$

If $\theta = \theta_{eq} = \pi$, then the following formulas should be used to avoid division by zero:

$$U_{new}[\theta = \theta_{eq} = \pi] = 0 \tag{S60}$$

$$\left.\frac{dU_{new}}{d\cos[\theta]}\right|_{\theta=\theta_{eq}=\pi} = k \tag{S61}$$

$$\left.\frac{d^2U_{new}}{(d\cos[\theta])^2}\right|_{\theta=\theta_{eq}=\pi} = 0 \tag{S62}$$

$$\left.\frac{dU_{new}}{d\theta}\right|_{\theta=\theta_{eq}=\pi} = -k\sin[\theta] \tag{S63}$$

$$\left.\frac{d^2U_{new}}{d\theta^2}\right|_{\theta=\theta_{eq}=\pi} = -k\cos[\theta] \tag{S64}$$

References

S1. W. C. Swope and D. M. Ferguson, Alternative expressions for energies and forces due to angle bending and torsional energy, *J. Comput. Chem.*, 1992, **13**, 585-594, DOI: 10.1002/jcc.540130508.

S2. D. Dubbeldam, K. S. Walton, T. J. H. Vlugt and S. Calero, Design, parameterization, and implementation of atomic force fields for adsorption in nanoporous materials, *Adv. Theory Simul.*, 2019, **2**, 1900135, DOI: 10.1002/adts.201900135.
